# Triply Responsive Control of Ion Transport with an Artificial Channel Creates a Switchable AND to OR Logic Gate

**DOI:** 10.1002/anie.202517444

**Published:** 2025-11-17

**Authors:** Javid Ahmad Malla, Kharina J. Fenton, Adinarayana Bellamkonda, Samuel I. Fidler, Mark I. Wallace, Charlie T. McTernan

**Affiliations:** ^1^ Artificial Molecular Machinery Laboratory The Francis Crick Institute 1 Midland Road London NW1 1AT UK; ^2^ Department of Chemistry Kings College London Britannia House 7 Trinity Street London SE1 1DB UK

**Keywords:** Azobenzene, Ion transport, Photoswitchable, Pillar arene, Self‐assembly, Supramolecular chemistry, Synthetic biology

## Abstract

Ion channels are ubiquitous in Nature, performing complex and essential tasks in our bodies. Synthetic chemists have begun to understand how to form artificial channels, which hold great promise as components in artificial cells, and in synthetic biology more widely. Future generations of these systems will be critical in the treatment of channelopathies. Despite advances in synthetic ion transporters, the current generation cannot approach the selectivity and controllability of the biological ion channels they seek to emulate, and multimodal control over activity remains hard to achieve. Herein, we present a synthetic ion channel whose activity can be controlled by three orthogonal stimuli (light, pH, guest/ligand), based on a pillar[5]arene functionalized with photoswitchable *ortho*‐tetrafluoroazobenzene moieties. We demonstrate excellent control over ten photoswitches (*E*‐to‐*Z* 82%, *Z*‐to‐*E* quant.). We show that the most active *Z*‐isomer forms dimeric ion channels in membranes. Single molecule planar bilayer conductance studies show high and low conductance states dependent on irradiation wavelength. We demonstrate that this activity can be modulated over 170‐fold by controlling pH, irradiation, and guest addition. We use these three stimuli to design a pH switchable AND to OR logic gate system, creating a powerful addition to the canon of synthetic ion channels.

## Graphical abstract

We report a triply responsive artificial ion channel, showing tuneable activity, and responsive to three distinct stimuli ‐ light, pH, and ligand – which we use to formulate an AND to OR switchable molecular logic gate.

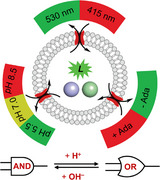

## Introduction

The complexity and functionality of biological systems emerges from the interactions of a web of complex molecular machinery.^[^ ^1^ ^]^ Membrane proteins have specialized functions; transferring signals, ions and molecules across membranes, to ensure homeostasis is maintained.^[^ ^2^, ^3^, ^4^ ^]^ Transmembrane ion channels constitute a unique protein class due to their importance in cell survival and the genesis of disease (e.g., cystic fibrosis, Dravet syndrome).^[^ ^5^ ^]^ When operative, they selectively transport biologically important ions such as Na^+^, K^+^, Ca^+^, Cl^−^, and HCO_3_^−^ across cellular and sub‐cellular membranes.^[^ ^6^ ^]^ Specific localization and activation of these channels allows cells to maintain a delicate balance between diverse ionic species. Gating of these channels by various signals and messengers allows cells to turn transport “on” or “off” as required.^[^ ^7^ ^]^ The transfer of information and cargo between the exterior and interior of a bilayer membrane is a key requirement in synthetic biology.^[^ ^8^ ^]^ An array of approaches has been proposed ‐ from using DNA origami,^[^ ^9^, ^10^, ^11^ ^]^ to modifying existing protein channels,^[^ ^12^, ^13^ ^]^ and the creation of de novo ion channels.^[^ ^14^ ^]^ However, the control of ion flow using multiple stimuli remains a key goal of the field.^[^ ^15^, ^16^ ^]^ Recent reports have explored using tethered photoswitches on transmembrane proteins to enable photocontrol of biological systems, with a focus on neurobiology applications.^[^ ^16^, ^17^ ^]^ However, proteins are extremely large, challenging to synthesize at scale, and unstable to enzymatic degradation. It is also difficult to tune biological channels away from their original targets. This limits the future application of such semisynthetic protein channels in synthetic biology. Fully synthetic transmembrane channels and transporters with stimulus control provide an alternate way to control these processes.^[^ ^18^, ^19^, ^20^, ^21^, ^22^, ^23^, ^24^, ^25^, ^26^ ^]^ There have been significant advances in the past decade with the development of fully synthetic ion channels and transporters which can respond to light,^[^ ^27^, ^28^, ^29^ ^]^ enzymes,^[^ ^30^ ^]^ pH,^[^ ^31^, ^32^ ^]^ glutathione,^[^ ^33^ ^]^ and dual‐stimuli.^[^ ^34^, ^35^, ^36^, ^37^ ^]^ However, multiple gating with three or more stimuli in a single discrete channel has yet to be explored. Furthermore, for synthetic systems to operate in biological settings, high levels of spatiotemporal control will be required; along with ways to tie activity to incorporation of information from multiple sources in a complex biological setting. Ion channels that act as logic gates are perfectly suited to this task, combining inputs to produce varied outputs;^[^ ^38^, ^39^, ^40^ ^]^ here we design a single channel that can be switched between operating as an “AND” or “OR” logic gate.

Pillar[n]arenes are a class of macrocycles that have been used to design ion^[^ ^41^, ^42^, ^43^ ^]^ and water transporting channels^[^ ^44^, ^45^ ^]^ in lipid membranes. These channels can be stable and are often functionalized with side chains to enhance membrane activity or provide selectivity. We anticipated that the installation of photoresponsive units in these scaffolds might enable increased control of activity, unlocking novel behaviour.^[^ ^46^ ^]^

**Figure 1:**
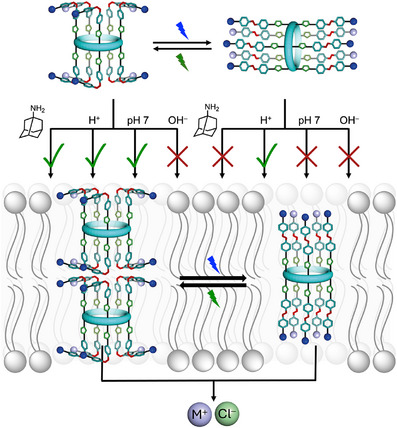
Schematic representation of channel in membranes, illustrating the three handles (light, pH, guest) used to control ion transport activity. Hill plot analysis suggests active species under 530 nm irradiation is a dimer.

Herein, we report a fully synthetic ion channel based on pillar[5]arene conjugated to an *o‐*tetrafluoroazobenzene^[^ ^47^ ^]^ via triazole linkers. We installed and identified three key motifs to enable multimodal control (Figure 1). First, we use fluorinated azobenzenes for high fidelity photoswitching, hypothesising that changing the length of the channel, and so its ability to span the membrane, would alter transport efficiency. Second, these azobenzene moieties are terminated with ten free carboxylates, enabling control of protonation state,^[^ ^48^ ^]^ and so charge, over an accessible range in liposomal systems. Finally, the cavities of pillar[n]arenes have well established host‐guest binding abilities,^[^ ^49^ ^]^ which we use to selectively block transport in one photoisomer of the channel. This allows us to demonstrate light, pH, and guest, controlled M^+^/Cl^−^ cotransport.

## Results and Discussion

**Scheme 1:**
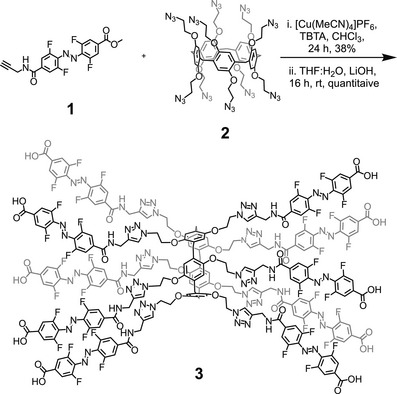
Synthesis of compound **3** from precursors **1** and **2**. Synthesis: i. **1** (12 eq.), **2** (1 eq.), [Cu(MeCN)_4_]PF_6_ (3 eq.), TBTA (3 eq.), CHCl_3_, 45 °C, 24 h, 38%. ii. LiOH (30 eq.), THF:H_2_O (2:1), rt, 16 h, quant.

We based the design of our channel on a pillar[5]arene core, as this motif has been shown to form effective, membrane active, ion and water channels by others.^[^ ^50^ ^]^ We next designed a photoswitchable unit, whose change in length on *E*‐*Z* isomerization we hypothesized would alter its ability to span the membrane. We chose to use *o*‐tetrafluoroazobenzene based switches, as their photophysical behaviour is well characterized, and their *E‐Z* photoswitching occurs with high fidelity; an essential parameter in a system with ten photoswitches operating in concert. Finally, we installed carboxylic acid groups at the termini of the arms of the channel, to enable water solubility and pH responsive behaviour.^[^ ^51^ ^]^ We also hoped that these groups would ensure the channel inserted in the membrane with the cavity parallel to the phospholipid tails, enabling ion flux by imposing a penalty for deleterious internalization of the negatively charged carboxylates in the hydrophobic lipid bilayer. Channel **3** was synthesized by the copper catalyzed azide‐alkyne cycloaddition of our alkyne functionalized photoswitch **1** and azide appended pillar[5]arene **2**,^[^ ^52^ ^]^ forming an intermediate methyl ester in 38% yield over ten simultaneous functionalizations. This methyl ester was immediately hydrolyzed with LiOH to generate the final channel **3** in quantitative yield,^[^ ^53^ ^]^ predominantly in the *E*‐state by ^1^H NMR spectroscopy (Schemes 1 and S1).

### Photoisomerisation Studies

**Figure 2:**
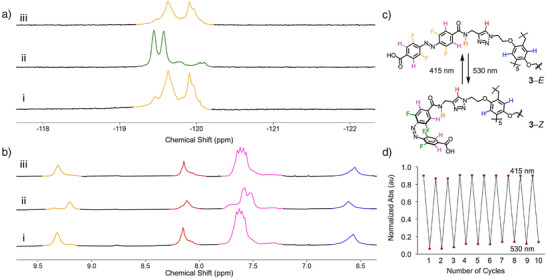
Characterization of isomerization of the *E*‐state to the *Z*‐state and *Z*‐state to the *E*‐state. a) ^19^F NMR of **3** in DMSO‐*d*_6_ (298 K, 376 MHz) and b) ^1^H NMR of **3** in DMSO‐*d*_6_ (298 K, 600 MHz) (i) as synthesized, (ii) after 5 min 530 nm irradiation and (ii) after 5 min 415 nm irradiation. c) Isomerisation of the *E*‐state to the *Z*‐state. d) Switching cycles for **3** (25 µM) monitored at 323 nm under alternating irradiation with 415 nm (*E*) and 530 nm (*Z*) light.

Having synthesized **3**, we first investigated its solution phase photoswitching behaviour, to confirm that we could efficiently isomerize the ten tetrafluoroazobenzene units. A solution of compound **3** (1 mM) in DMSO was irradiated with 530 nm LEDs (see Supporting Information, Section 3 for details) for 5 min and monitored by ^19^F NMR, ^1^H NMR, Diffusion Ordered ^1^H NMR Spectroscopy (DOSY) and UV–vis absorbance measurements (Figures 2 and S26). By ^19^F NMR, we observe shifts in the aromatic fluorine signals from 119.82 to 119.52 ppm, along with changes in structure and line shape (Figure 2a, green), confirming isomerization of the tetrafluoroazobenzenes to the *Z*‐state. Similarly, ^1^H NMR shifts (Figure 2b) showed isomerization of the tetrafluoroazobenzenes to the *Z*‐state (82% conversion by integration of ^1^H and ^19^F spectra). Furthermore, we saw a shift in UV–vis absorbance (25 µM) maximum from 420 to 470 nm, corroborating isomerization to the *Z*‐state (Figure 2d).^[^ ^37^ ^]^ Finally, we confirmed effective isomerization DOSY NMR, where we see an increase in hydrodynamic radius from 1.35 to 1.42 nm when we irradiated with 530 nm LEDs (Figure S27).

We next explored the conversion of the *Z*‐state to the *E*‐state with 415 nm LED irradiation for 5 min, in 1 mM DMSO solution. The same methods were used to monitor isomerization. Upon 415 nm irradiation the ^19^F NMR signals showed reemergence of peaks at 119.82 (Figure 2a, orange) with full reversion to the *E*‐state, and loss of signals for the *Z*‐state. Likewise, ^1^H NMR showed the reappearance of signals corresponding to the *E*‐state (Figure 2b, quant. conversion). UV–vis absorbance (25 µM) studies showed the migration of the absorbance maxima to 420 nm, corresponding to the *E*‐state. Collectively, these experiments confirmed that the *E*‐state can be isomerized to the *Z*‐state with 87% conversion using a 530 nm LED, and the *Z*‐state can be isomerized back to the *E*‐state using a 415 nm LED with quantitative conversion. The photoswitches of **3** showed high resistance to fatigue (Figures 2d and S28), with no significant loss of efficiency in 10 cycles.

### Ion Transport Studies

**Figure 3:**
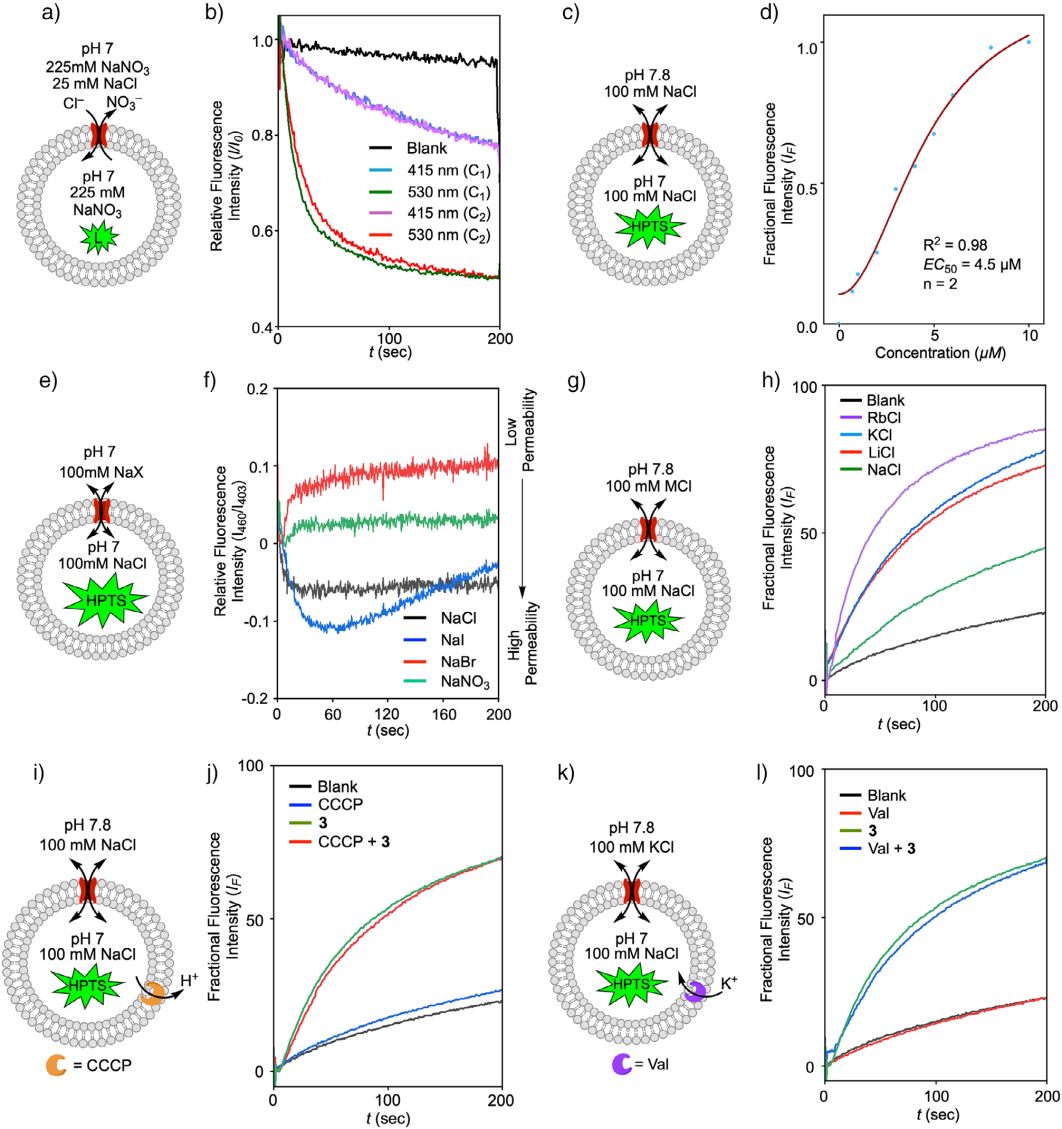
Ion Transport Activity of 3. a) Schematic representation of ion transport facilitated by **3** across POPC‐LUVs⊃lucigenin and b) ion transport facilitated by **3** (1 *µ*M) across POPC‐LUVs⊃lucigenin after irradiating with 415 or 530 nm irradiation (C_1_ and C_2_ indicate cycle 1 and cycle 2 respectively). c). Schematic representation of ion transport facilitated by **3** across POPC‐LUVs⊃HPTS and d) Hill plot of compound **3** irradiated with 530 nm (**3**_530_) at *t* = 170 s showing *n* = 2 and so formation of dimeric channels in the membrane. e) Schematic representation of anion gradient assay using POPC‐LUVs⊃HPTS and f) Anion preference evaluated by anion gradient assay. g) Schematic representation of cation selectivity of **3** across POPC‐LUVs⊃HPTS and h) Cation selectivity evaluated by pH gradient assay of **3**_530_. i) Schematic representation and j) ion transport facilitated by **3**_530_ across POPC‐LUVs⊃HPTS in the presence and absence of CCCP (0.5 *µ*M). k) Schematic representation and l) ion transport facilitated by **3**_530_ across POPC‐LUVs⊃HPTS in the presence and absence of valinomycin (2 *p*M). i)–k) support co‐transport by **3** under these experimental conditions.

Having demonstrated efficient photoswitching with **3** in solution, we next looked to evaluate the transmembrane ion transport activity of **3** in both the *E*‐ and the *Z*‐states, to understand whether we could use this to control transmembrane communication. We assessed the ion transport ability of **3** across large unilamellar vesicles (LUVs) composed of 1‐palmitoyl‐2‐oleoyl‐sn‐glycero‐3‐phosphocholine (POPC) encapsulating lucigenin dye, POPC‐LUVs⊃lucigenin, enabling us to monitor chloride ion transport facilitated by **3**, using a well‐established assay based on chloride mediated quenching of lucigenin.^[^ ^54^, ^55^, ^56^, ^57^, ^58^, ^59^ ^]^ The initial rate of transport calculated from the fluorescence measurements showed that the *Z*‐state is 12‐fold more active than the *E*‐state (Figure 3a,b and Table S1). Similar transport rates were found over two distinct subsequent photoswitching cycles (C_1_ and C_2_). To further understand the behaviour of our transport system, we moved to using POPC LUVs encapsulating 8‐hydroxypyrene‐1,3,6‐trisulfonic acid (HPTS) dye, POPC‐LUVs⊃HPTS at pH 7 (Figure S30) and first performed a dose‐dependent ion transport study of both the *E‐* and the *Z*‐states using a pH gradient.^[^ ^60^, ^61^ ^]^ The dose‐dependence study of the *E*‐state could not be completed due to precipitation of the compound at higher concentrations (above 5 *µ*M). However, the dose‐dependence of the *Z*‐state showed clear concentration dependent transport activity with saturation at 9 *µ*M (Figure S31). Hill analysis confirmed the half maximal effective concentration (*EC*_50_) was 4.5 *µ*M and the Hill coefficient (cooperativity) was two, which suggests that the major route to ion transport in the *Z*‐state involves dimerisation in the membrane to form active transporters (Figure 3c,d).^[^ ^62^ ^]^ Having demonstrated that the *Z*‐state of **3** formed an effective transport system, we next looked to identify the ion transport mechanism of the *Z*‐state. To do so, we first evaluated the anion transport ability of **3** across POPC‐LUVs⊃HPTS by using anion gradient assay recently reported by Gale's group.^[^ ^63^ ^]^ In this assay, POPC‐LUVs⊃HPTS with NaCl_in_/NaX_out_ (where X indicates a different anion) were used without any pH gradient. The anion gradient provides the driving force for Cl^−^/X^−^ exchange, which in turn results in either H^+^/X^− ^influx or H^+^/Cl^− ^efflux, depending on transporter selectivity. This changes intravesicular pH, leading to change in florescence intensity of the pH responsive HPTS dye. The assay showed a significant change in the HPTS fluorescence intensity dependent on the identity of the external anions, confirming the chloride and iodide were preferred over nitrate and bromide ions (Figures 3e,f and S32). Next, we evaluated the involvement of cations in the transport mechanism of **3** across POPC‐LUVs⊃HPTS, by varying the extravesicular MCl salts (M^+^ = Li^+^, Na^+^, K^+^, and Rb^+^). Differential rates of transport with different cations (Figure 3g,h) were observed, suggesting that cations are involved in the transport process. Collectively, these results demonstrate that compound **3** forms a M^+^/Cl^−^ cotransport system in liposomal bilayer membranes, most likely favouring electroneutral transport under these conditions.

These studies prompted us to further confirm the presence of a cotransport mechanism. To do so, we used carbonyl cyanide *m*‐chlorophenyl hydrazone (CCCP) and valinomycin coupled assays.^[^ ^60^, ^64^, ^65^ ^]^ Under an applied pH gradient, CCCP enables the efflux of H^+^ from liposomes. For a Cl^−^/X^−^ electrogenic mechanisms, the presence of CCCP (at a concentration too low to generate a significant signal alone) would enhance the rate of transport due to coupling of two complementary transporters (the test transporter and CCCP). However, there was no significant difference in the rate of transport of **3** in the presence and absence of CCCP (Figure 3i,j), indicating no coupling and so the primary mechanism in operation is not electrogenic.^[^ ^66^ ^]^

We then evaluated the transport activity of **3** in the presence and absence of valinomycin, a selective potassium transporter,^[^ ^60^, ^67^ ^]^ which again showed no significant difference in the rate of transport of **3** (Figure 3k,l). Collectively, these results support our assignment that the primary mode of operation of **3** is a cotransport mechanism and hence electroneutral tranport.^[^ ^68^ ^]^ To differentiate between symport and antiport processes, we carried out a modified lucigenin assay using POPC vesicles entrapping NaCl (225 mM). The outer buffer was changed to isoosmolar Na_2_SO_4_ and Cl^−^ efflux was monitored over time.^[^ ^55^ ^]^ This experiment was also performed with extra‐vesicular isoosmolar NaNO_3_ buffer. The similar transport rate in both conditions (Figure S35), supports that a symport mechanism is dominant under these assay conditions.^[^ ^69^ ^]^

Next, we looked to confirm whether our transporter operated by a carrier or channel mechanism. As such, we first explored the *E‐* and *Z*‐states of **3** in 1,2‐dipalmitoyl‐glycero‐3‐phosphocholine (DPPC) liposomes encapsulating with HPTS; DPPC‐LUVs⊃HPTS. DPPC undergoes a phase transition from an ordered (gel) phase to a liquid disordered (fluid) phase at 41 °C.^[^ ^70^ ^]^ Channel activity should be minimally affected by moving from the gel to the fluid state, whilst carrier activity should be significantly inhibited in the gel state, as it requires mobility through the membrane, and so experiences a higher penalty in the fluid‐gel transition. Channel activity can also be moderated by this transition, particularly when changes in lateral compression in the membrane are important. Compound **3** was incubated in DPPC‐LUVs⊃HPTS then irradiated with 415 or 530 nm LEDs, and ion transport was measured at both 25 °C and 45 °C. When **3** was irradiated with 415 nm, to form the *E*‐state, there was minimal activity at 25 °C but significant activity at 45 °C (Figure S37). However, when **3** was irradiated with 530 nm changes were seen between 25 °C and 45 °C (Figure S37A,B). We carried out in situ photoswitching with **3** preincorporated in DPPC liposomes at 25 °C monitor the changes in the transport rate (Figure S37C,D), which shows efficient switching for two consecutive cycles, suggesting that **3** is not ejected from the membrane during photoswitching. These results suggest that the *Z*‐state provides a stable pathway for ion flux even at 25 °C, and so that it operates as an ion channel, whilst the activity of the *E*‐state decreases in the gel‐state of the bilayer membrane at 25 °C. This could either be due to the *E‐*state acting as a transporter, or the gel‐state favouring a collapsed channel. Transport experiments with POPC‐LUVs⊃HPTS and potassium gluconate (Figure S38) revealed that the transport activity of compound **3** is unaffected by fatty acid impurities.^[^ ^71^ ^]^ Finally, we confirmed that our transporters do not cause significant membrane lysis using a carboxyfluorescein self‐quenching assay (Figure S39) where we see low levels of lysis (<15%) for both isomers of transporter.^[^ ^72^ ^]^

**Figure 4:**
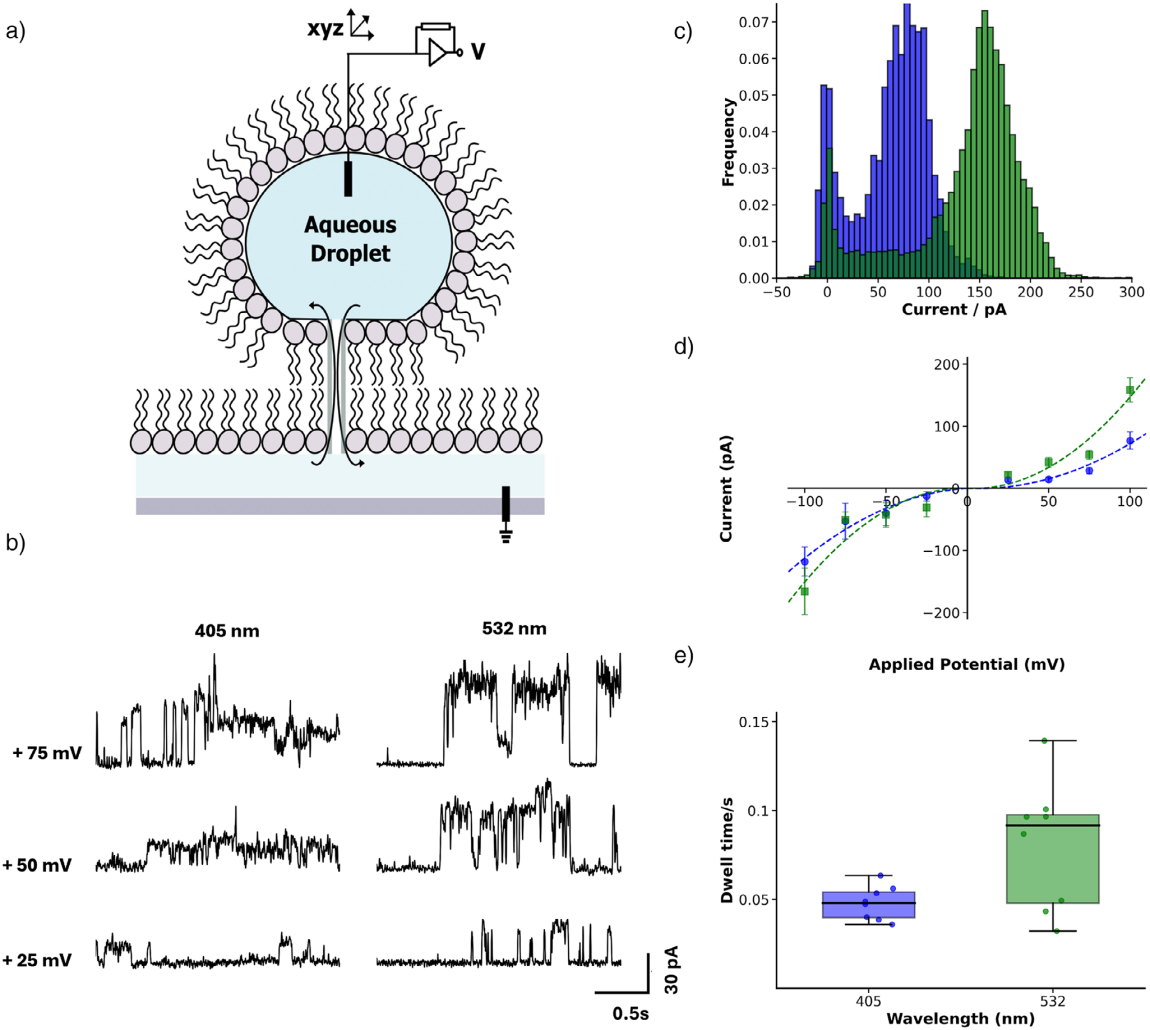
Planar Bilayer conductance studies of 3: a) Schematic representation of the droplet interface lipid bilayers used here. b) Representative segments of current recorded from DPhPC bilayers containing compound **3** pre‐exposed to both 405 and 532 nm light at various applied potentials. c) All‐point histogram of currents measured at + 100 mV (405 nm, blue, and 532 nm, green). d) *I‐V* Relationship: Mean currents derived from histograms. Dashed lines represent quadratic fits applied to the data, revealing a level of rectification. e) Measured Tau values (opening probabilities) of the *E*‐(405 nm, blue) and *Z*‐(532 nm, green) states at + 100 mV, with the *Z*‐state showing longer opening durations.

To unambiguously assign the mode of action of our transporters as that of ion channels, we carried out single channel recording of both the *E*‐ and *Z*‐states using droplet interface bilayers (DIBs).^[^ ^73^ ^]^ Electrical recordings were performed after bilayer formation of lipid bilayers incorporating **3** (1 nM) in 1.5 M KCl HEPES solution, pH 7, in the nanolitre aqueous droplet. We observed distinct current traces consistent with single channel gating events for both isomers, suggesting electrogenic transport is also operating. The *Z*‐state showed higher current amplitudes. This is consistent with our liposomal data (Figure 3a), suggesting *Z* is the more active transporter (Figure 4b,c). We see multiple subconductance states in our single‐channel recordings, particularly at higher voltages. This likely reflects the complex conformational landscape, and dynamic pore geometries, in these systems, with multiple stable confirmations in the membrane with distinct conductance levels (Supporting Information, Section 6). However, our data confirm clear distinctions between the two photoisomers, confirming that irradiation effectively induces a structural switch resulting in a measurable change in the channel's functional properties. The *I–V* plot for each isomer (Figure 4d) provides an estimate of molar conductance of the *E*‐state of 10.3 pS at positive voltages and 14.0 pS at negative voltages, whereas the *Z*‐state showed increased conductance of 20.4 and 20.7 pS at positive and negative voltages, respectively. Kinetic analysis of channel gating showed that the “open” probability of the *Z*‐state was larger than that of the *E*‐state, providing further justification of the higher conductance of the *Z*‐state (Figures 4e and S69). To understand the behaviour of the *E*‐ and the *Z*‐states in the membrane, we modelled optimised geometries in a GFN2‐xTB forcefield, in a solvent field of octanol (Supporting Information, Section 7). We found that the *E*‐state could efficiently intramolecularly stack *o*‐tetrafluoroazobenzenes, so closing the channel to ion transport, whilst in the *Z*‐state these stacking interactions were disfavoured, leading to a more open channel. This provides a potential explanation of the difference in activity between the two isomers that we observe. We further modelled a dimerised *Z*‐state channel, showing that this alignment is feasible (Figure S71).

Having shown that we had formed an effective transmembrane ion channel, able to perform selective M^+^/Cl^−^ symport, and whose activity could be controlled by light, we looked to expand our control over ion transport, leveraging the intrinsic properties of our channel. We identified two handles which might allow us to further control and direct transport activity – blocking the cavity of the central pillar[5]arene, and tuning the protonation state of the carboxylic acid termini of the “arms” of our channel.

**Figure 5:**
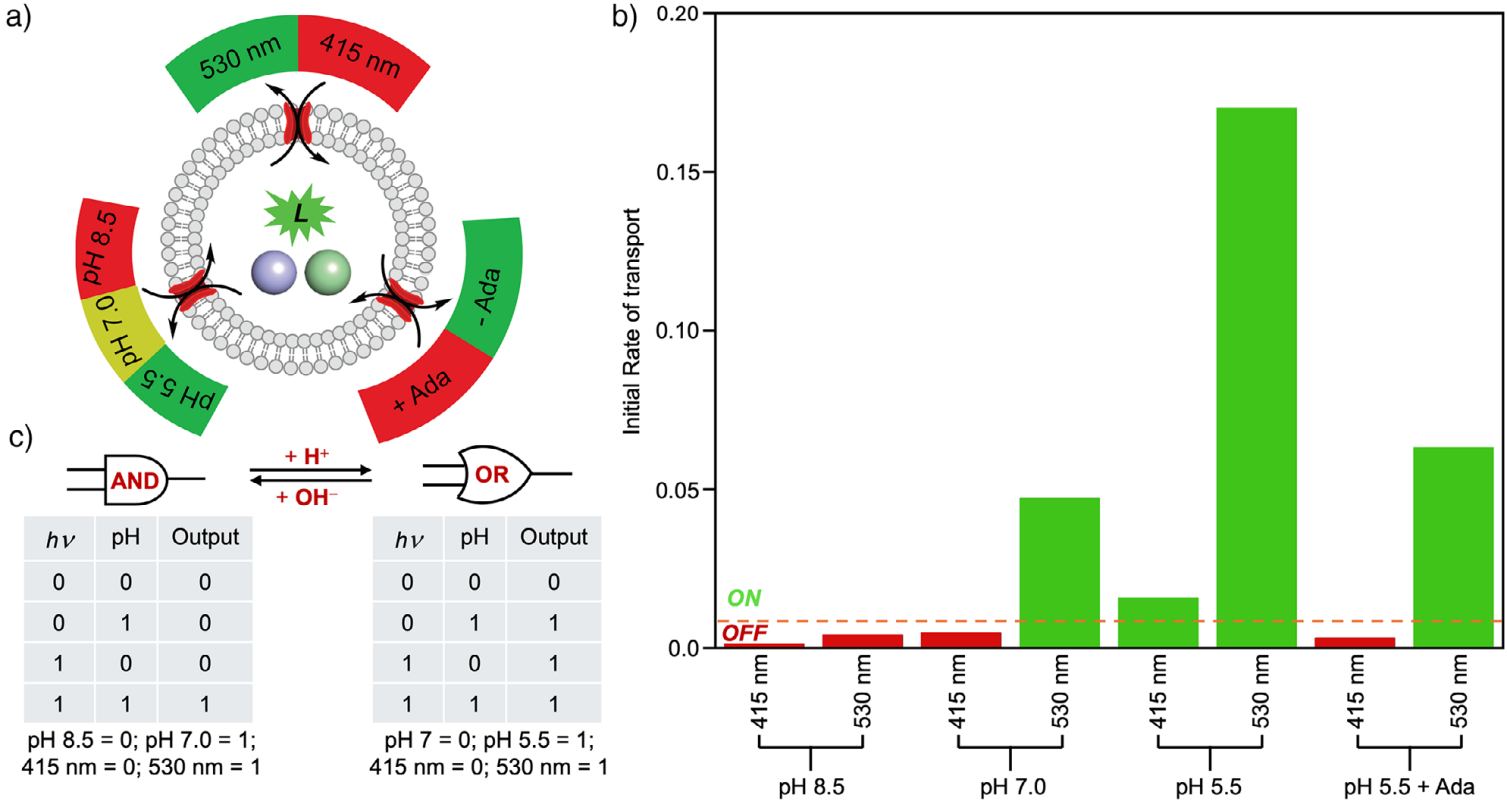
Stimulus responsive ion transport of 3 in POPC‐LUVs⊃Lucigenin. a). Schematic representation of different stimuli controlling ion transport of **3**. b). Initial rate of transport facilitated by **3** (in both *E*‐ and Z‐states) with different stimuli (red shows low activity states, green shows high activity states). c) pH‐controlled logic gate switching using initial rates of transport as a readout signal, and a threshold of 5% maximal activity.

To probe this, we first evaluated the transport activity of **3** (in both the *E*‐and the *Z*‐states) under different pH conditions using POPC‐LUVs⊃lucigenin. At pH 7, the *Z*‐state is more active than the *E*‐state as we had previously observed. The transport rate decreases for both the *E*‐ and the *Z*‐states at pH 8.5, suggesting that excessive negative charge interferes with membrane insertion and/or dimerization along with repulsion of negatively charged anions. We found that at pH 5.5, the rate of transport increases for both the *E*‐ and the *Z*‐states (Figure 5b and Table S1). We find a 42‐fold increase in activity for the *Z*‐isomer between pH 8.5 and pH 5.5. These results suggest that the terminal carboxyl groups play an important role in the penetration of **3** (in both the *E*‐and *Z*‐states) into liposomal membranes, and to the dimerization process. They also confirm that tuning the protonation state, and so charge state of the termini, acts as an effective handle to modulate transport activity in our system, along with photoisomerization.

We next explored whether an external ligand could be used to block the cavity of the pillar[5]arene, and so provide an additional modulatory handle via host‐guest chemistry. To do so, we used the same assay at pH 5.5 and added 1‐aminoadamantane hydrochloride (**S4**) as an external guest. We found that addition of 1‐aminoadamantane effectively inhibited the *E‐*state, whereas addition to the *Z‐*state had significantly less effect on overall activity (Figures 5b and S41). Whilst we had initially expected both isomers to be inhibited by blocking their cavities, we assign this unanticipated selectivity to differential binding to the dimerised *Z*‐state versus the monomeric *E*‐state in the membrane environment (Figures S61–S66). We calculated the initial rates of transport in each case (Table S1), which show significant differences in the rate of transport between the *E‐* and the *Z*‐state, which is 4‐fold at basic pH (pH 8.5), 12‐fold at neutral pH (pH 7.0), 11‐fold at acidic pH (pH 5.5), and 21‐fold at acidic pH (pH 5.5) in the presence of 1‐aminoadamantane. We were able to demonstrate a 170‐fold increase in initial rate between least active (*E‐*state, pH 8.5) and most active (*Z‐*state, pH 5.5) conditions, providing a high level of control for potential future applications in synthetic biology. Collectively, these results demonstrate that the transport activity of **3** can be controlled over a broad range,^[^ ^74^, ^75^ ^]^ by at least three different, independent, handles, making **3** uniquely responsive to three orthogonal stimuli. The differential rates of ion transport under different conditions can be formulated as molecular logic gates,^[^ ^16^, ^35^, ^38^, ^39^, ^40^, ^76^, ^77^, ^78^ ^]^ with an ON/OFF threshold of c. 5% of maximum initial rate. When considering changes in pH (8.5 versus 7.0) and light irradiation (415 nm versus 530 nm), we find that the rates of initial transport provide an AND logic gate, where both pH and light must be in the preferred (“1”) state to trigger transport. By moving to a more acidic pH (7.0 versus 5.5), the same system now formulates an OR logic gate, transporting when either signal is present (Figure 5c). As such, our triply responsive channel can be used as a pH switchable logic gate.

## Conclusion

We have synthesized a triply responsive artificial ion channel showing tuneable activity, which is responsive to three distinct stimuli (light, pH, guest). We have shown that these act as highly effective channels both in liposomes, and in single molecule planar lipid bilayer experiments. Our design establishes the power of addressing each component of a complex supramolecular system in turn – here, light enables controlled photoisomerization of highly efficient *o*‐tetrafluorobenzene switches; pH modulates the protonation state of peripheral carboxylic acids and so dimer formation and membrane insertion; addition of a guest which binds in the central cavity of the pillar[5]arene inhibits the flow of ions. As such, **3** represents a versatile platform to explore potential applications as a controllable, artificial ion channel in artificial cells, and in the design of targeted treatments for channelopathies. Furthermore, we also show how **3** can be used as a molecular logic cage, decoding of complex inputs and providing clear outputs for information processing. Future work will focus interfacing these switchable channels with biological systems

## Author Contributions

J.A.M. conducted the synthesis with the assistance of AB. J.A.M. designed and carried out isomerization experiments and transport assays and analysed the results with CTM. S.F. performed molecular dynamics and modelling studies. J.A.M. and C.T.M. conceived and designed project and drafted the paper. K.F. and M.M.W. carried out the electrophysiology studies and analysed the data. C.T.M. supervised the project and obtained funding. All authors edited the final manuscript.

## Conflict of Interests

The authors declare no conflict of interest.

## Supporting information



Supporting Information

## Data Availability

The data that support the findings of this study are available in the Supporting Information of this article.
