# Supplementary material for: Triply Responsive Control of Ion Transport with an Artificial Channel Creates a Switchable AND to OR Logic Gate
Source: Angew Chem Int Ed Engl. 2025 Nov 17;65(2):e17444. doi: 10.1002/anie.202517444 (PMC12790355; doi:10.1002/anie.202517444)
Supplement: Supplementary file 1 — Supporting Information [file ANIE-65-e17444-s001.docx]

**Triply Responsive Control of Ion Transport with an Artificial Channel Creates a Switchable AND to OR Logic Gate**

# Javid Ahmad Malla,^1,2^ Kharina J. Fenton,^1,2^ Adinarayana Bellamkonda,^1,2^ Samuel I. Fidler,^1,2^ Mark I. Wallace,^2,^* Charlie T. McTernan^1,2,^*

*^1^Artificial Molecular Machinery Laboratory, The Francis Crick Institute, 1 Midland Road, London, NW1 1AT, UK. ^2^ Department of Chemistry, Britannia House, 7 Trinity Street, King’s College London, SE1 1DB, UK.*

***Corresponding author

**Table of Contents**

**1. General Information S3**

**2. Synthetic Experimental Procedures S4**

*Synthesis of Alkyne-Carboxylic Acid Photoswitch* **S3 S5**

*Synthesis of Alkyne-Ester Photoswitch* **1 S11**

*Synthesis of Photoswitch* **3 S17**

**3. Photo-Isomerization Experiments S29**

*Photofatigue Experiments* **S30**

**4. Ion Transport Assays S31**

*Chloride Transport Across POPC‒LUVs*⊃*Lucigenin Vesicles* **S31**

*Ion Transport Activity Studies Across POPC‒LUVs⊃HPTS* **S33**

*Dose-response Activity in POPC‒LUVs*⊃*HPTS* **S35**

*Ion Selectivity Studies*  **S39**

*Mechanism of Ion Transport*  **S41**

*Ion Transport Across DPPC‒LUVs*⊃*HPTS*  **S42**

*Effect of Fatty Acids on Rate of Transport* **S44**

*Vesicle leakage studies by* *Carboxyfluorescein assay* **S44**

*Ion Transport Across POPC‒LUVs⊃Lucigenin Vesicles* **S45**

**5. NMR Titrations - Host-Guest Association** **S59**

**6. Planar Bilayer Conductance Studies S63**

**7. Computational Calculations S67**

**8. References S70**

# General Experimental

***Synthesis****:* Unless otherwise stated, all reagents, including anhydrous solvents, were purchased from commercial sources and used without further purification. Reactions were carried out under ambient conditions unless otherwise stated. An SP Scientific Bench Top Pro Lyophilizer was used for drying the samples when required. Chromatographic separation was performed using a Biotage Isolera equipped with Biotage Sfar Duo column cartridges of appropriate phase and size. Unless otherwise stated, crude material was purified by dissolution in a minimum volume of solvent and loaded directly onto equilibrated cartridges; size exclusion Chromatography was performed using SX-1 beads pre-swollen in CH_2_Cl_2_.

***Analysis****:* NMR spectra were acquired on a Bruker Ascend 400 equipped with a BBO Smart Probe, an Avance III 600 equipped with a ^1^H/^13^C/^15^N triple-resonance PFG cryoprobe at a constant temperature of 298 K unless stated otherwise. Chemical shifts are reported in parts per million from low field to high field and referenced to residual solvent or internal standard. Coupling constants (*J*) are reported in Hertz (Hz), and standard abbreviations indicating multiplicity were used as follows: m = multiplet, sext. = sextet, quint. = quintet, q = quartet, t= triplet, d = doublet, s = singlet, app. = apparent and br. = broad. Signal assignment was carried out using 2D NMR methods (HSQC, COSY) where necessary. Low resolution mass spectrometry and UPLC analysis was performed on a Waters Acquity H-class UPLC coupled with a Waters SQD2 mass spectrometer. Chromatographic separation was performed on an ACQUITY UPLC BEH C_18_ column (130 Å, 1.7 µm, 2.1 x 50 mm) with the following conditions: Solvent A: H_2_O:MeCN (95:5) + 2 mM ammonium acetate; Solvent B: MeCN; gradient of 0 – 100% B over 2 min. The instrument was operated at a flow rate of 0.8 mL min^-1^ with a column temperature of 45 °C and an injection volume of 5 μL. HRMS High resolution mass spectrometry was performed using an Orbitrap Exploris 120 operating at 0.2 mL min^-1^ using Optima MeOH as eluent without chromatographic separation. High resolution mass spectrometry data was analyzed using FreeStyle software. UV-Vis experiments were carried out on a Shimadzu 2600i Spectrophotometer.

Florescence measurements were performed using FS-5 fluorometer (Edinburgh Instruments) connected to a temperature controller using Hellma quartz cuvettes. Lipids were purchased from Avanti Polar Lipids. HEPES buffer, HPTS, Lucigenin, Triton X‒100, NaOH, and inorganic salts were purchased of molecular biology grade from Sigma. Large unilamellar vesicles (LUVs) were prepared by using a mini extruder, equipped with a polycarbonate membrane of 100 nm or 200 nm pore size, purchased from Avanti Polar Lipids. Sephadex G-50 was used for Size exclusion Chromatography to isolate liposomes from dyes in their respective buffers.

Synthesis of **S1** was carried out following a reported protocol.^S1^ Synthesis of decaazidopillar[5]arene **2** was carried out using a modification to a reported protocol.^S2^

# Synthetic Experimental Procedures

# Scheme S1: Synthetic scheme for synthesis of 3.

**Synthesis of Alkyne-Carboxylic Acid Photoswitch** **S3**.

# In a 50 mL round bottomed flask, compound S1 (500 mg, 1.46mmol, 1 eq.,) was suspended in anhydrous THF (15 mL). To this, HATU (556 mg, 1.46 mmol, 1.0 eq.) and DIPEA (330 µL, 1.90 mmol, 1.3 eq.) was added, and reaction was stirred for 15 minutes. Propargylamine, S2, (110 µL, 1.75 mmol, 1.2 eq.) was added, and the solution was stirred for 3 h at RT. THF was removed under reduced pressure and the crude mixture dissolved in ethyl acetate (70 mL), followed by extraction against NaCl_(sat)_ (30 mL) and H_2_O (30 mL). The organic phase contained product S3 and was purified by reverse phase column chromatography using a C18 column and MeCN/water system and a 5-95% gradient containing 0.1% formic acid. The product was isolated as a red solid (225 mg, 593 µmol, 40%). ^1^H NMR (400 MHz, DMSO) δ: 9.31 (t, *J* = 5.5 Hz, 1H_c_), 7.81 (dd, *J* = 16.0, 9.6 Hz, 4H_d_), 4.11 (dd, *J* = 5.5, 2.6 Hz, 2H_b_), 3.20 (t, *J* = 2.5 Hz, 1H_a_). ^13^C NMR (151 MHz, DMSO) δ: 163.14, 155.67 (d, *J* = 19.9 Hz), 153.92 (d, *J* = 16.0 Hz), 150.11, 138.25, 133.33, 132.61, 73.95, 29.27. ﻿^19^F NMR (376 MHz, DMSO) δ -119.98 (d, J = 8.7 Hz), -120.30 (d, J = 10.0 Hz). HRMS: m/z C_17_H_8_F_4_N_3_O_3_^–^, observed 378.0461 (calc. 378.0507). *∆* = −12 ppm.

**
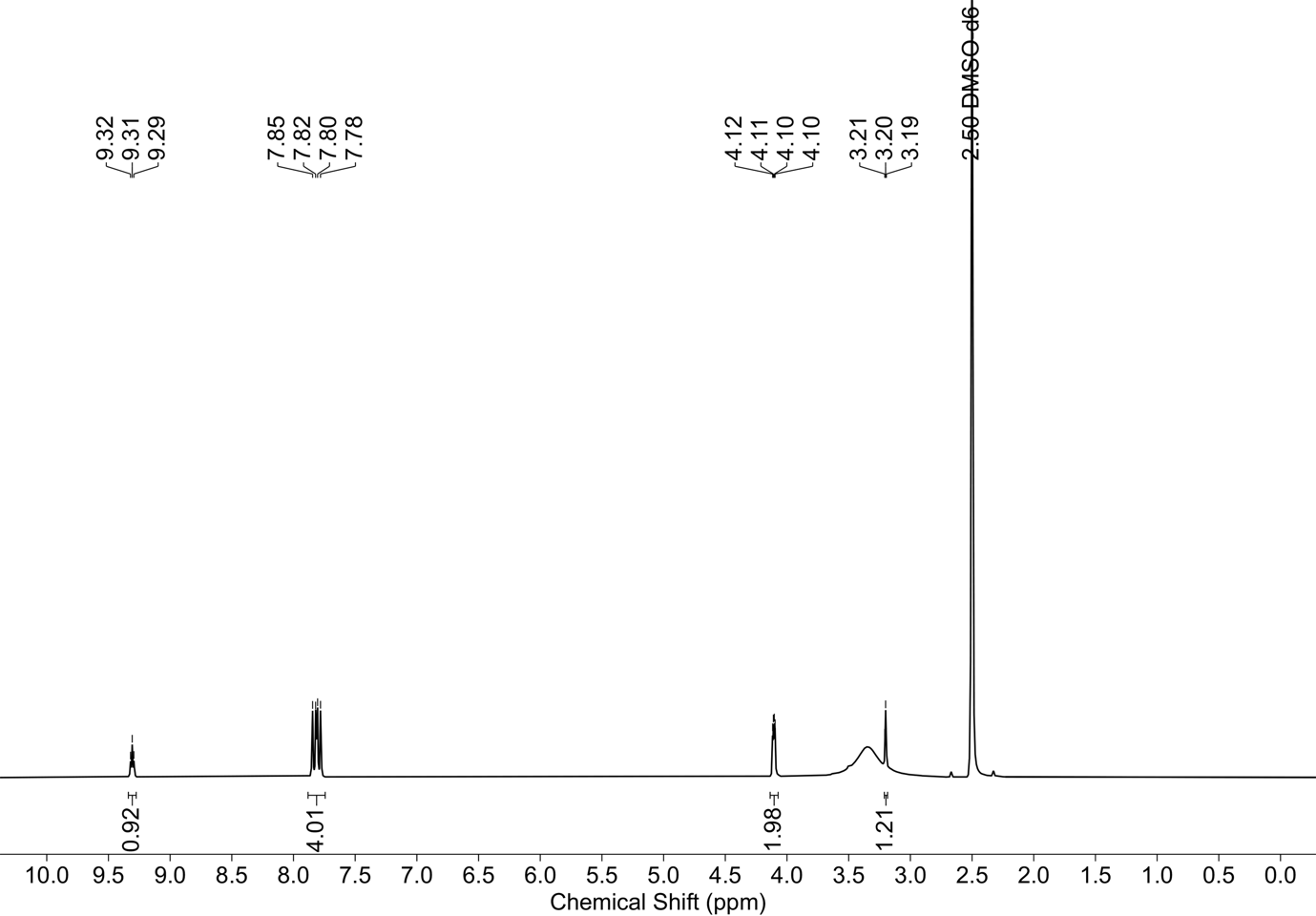
**

**Figure S1. ^1^H NMR (DMSO-*d*_6_, 400 MHz, 298 K) of S3.**

**
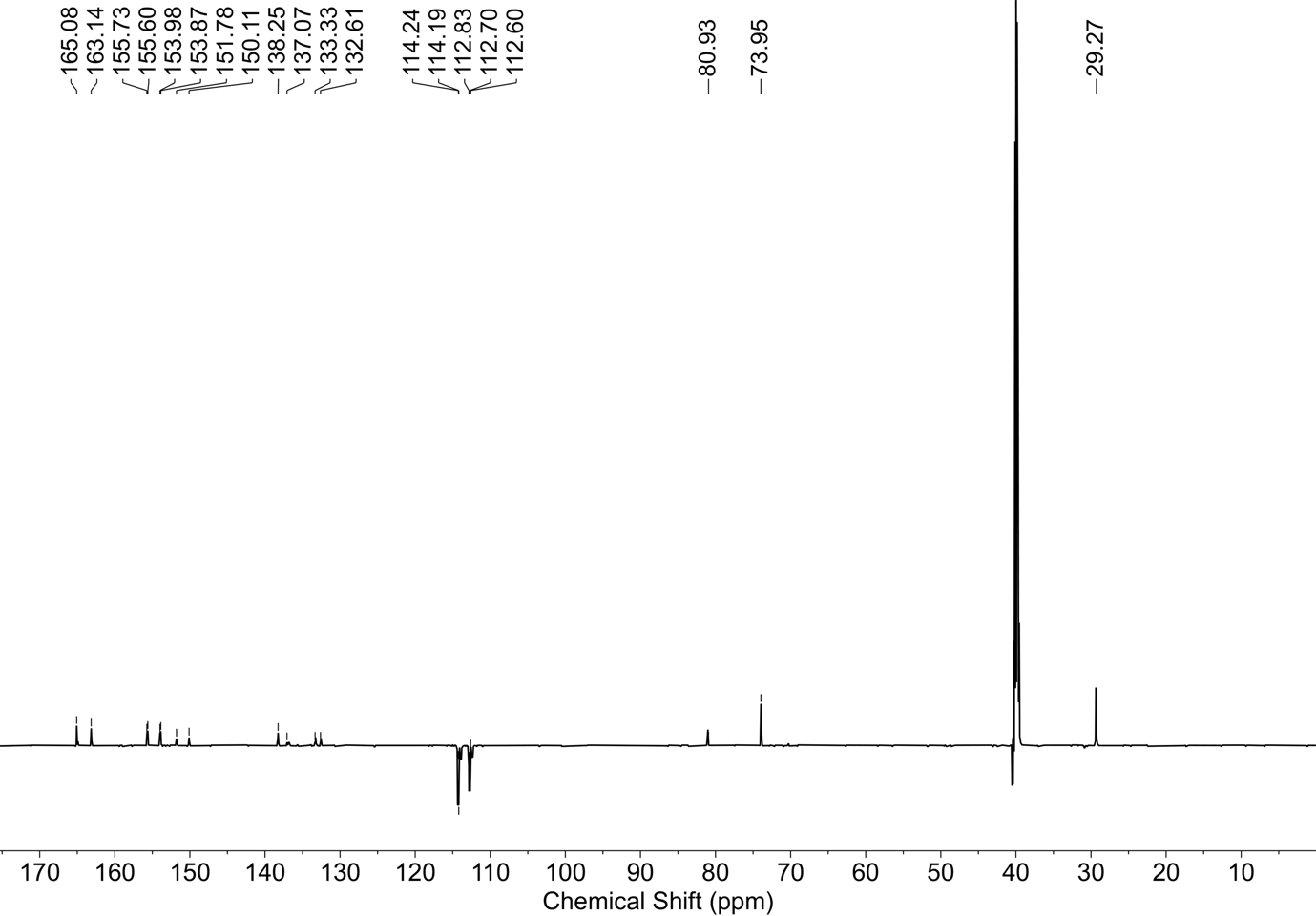
**

**Figure S2. J MOD NMR (DMSO-*d*_6_, 600 MHz, 298 K) of S3.**

**
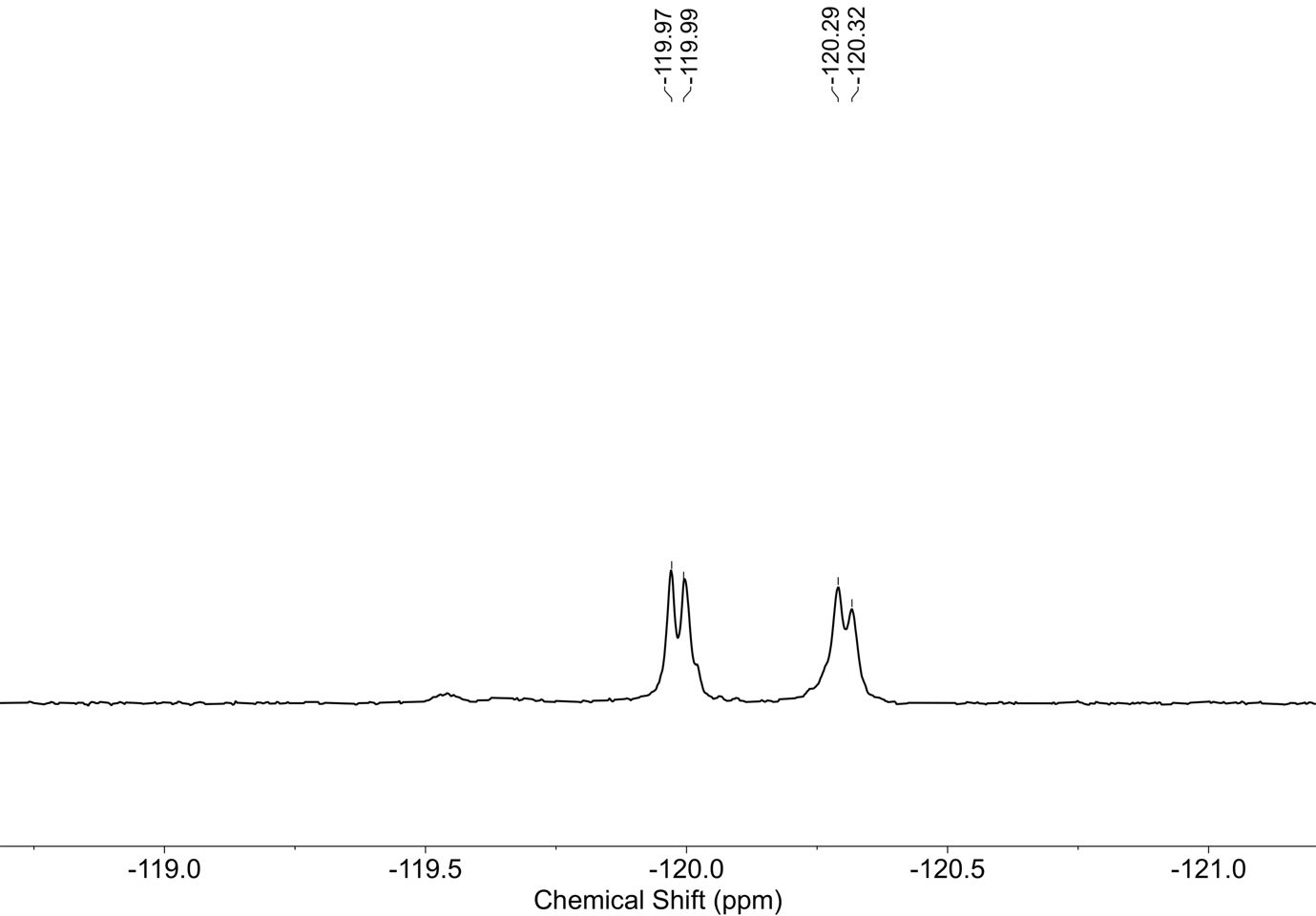
**

**Figure S3. ^19^F NMR (DMSO-*d*_6_, 376 MHz, 298 K) of S3.**


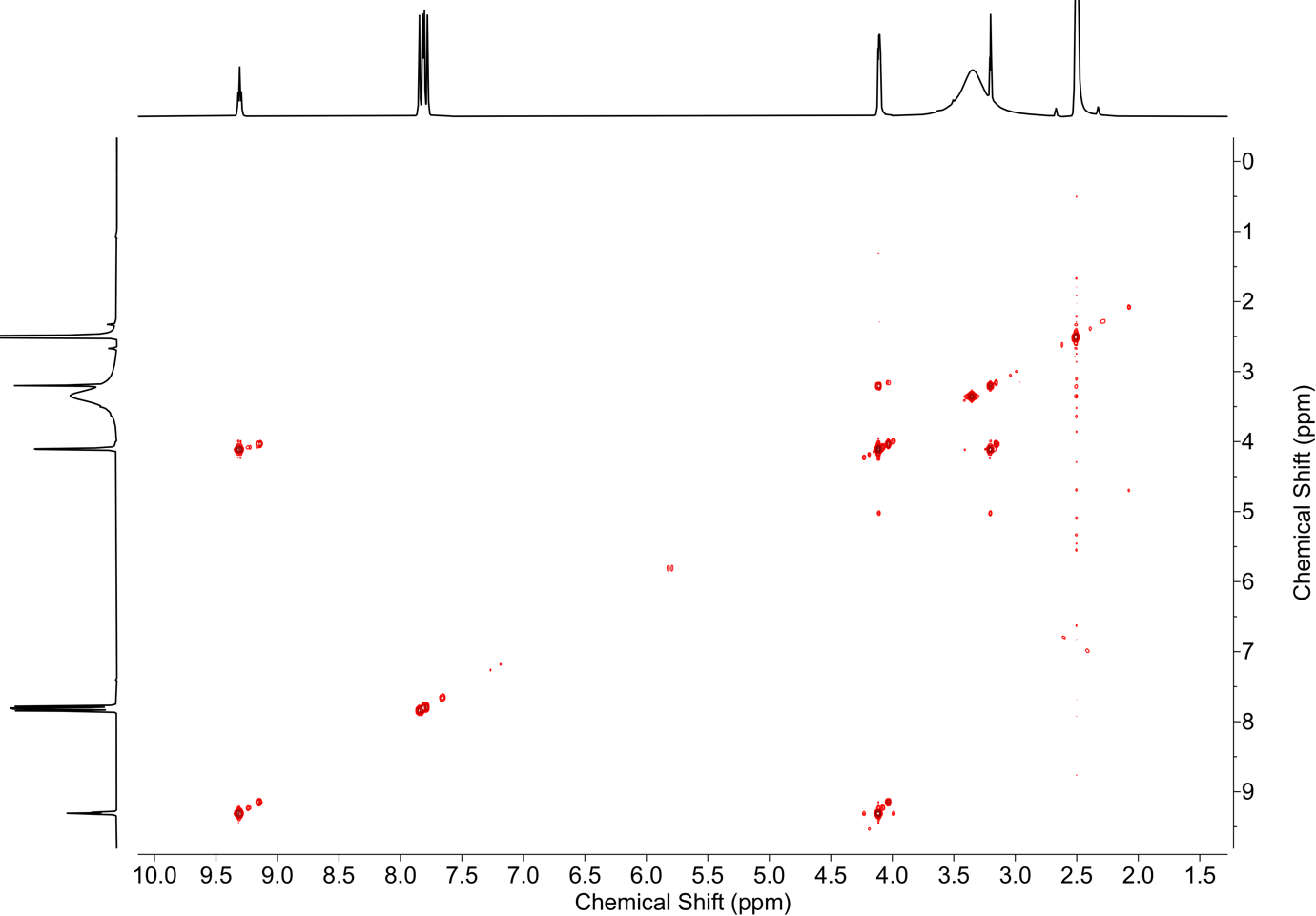
**Figure S4. COSY NMR (DMSO-*d*_6_, 600 MHz, 298 K) of S3.**


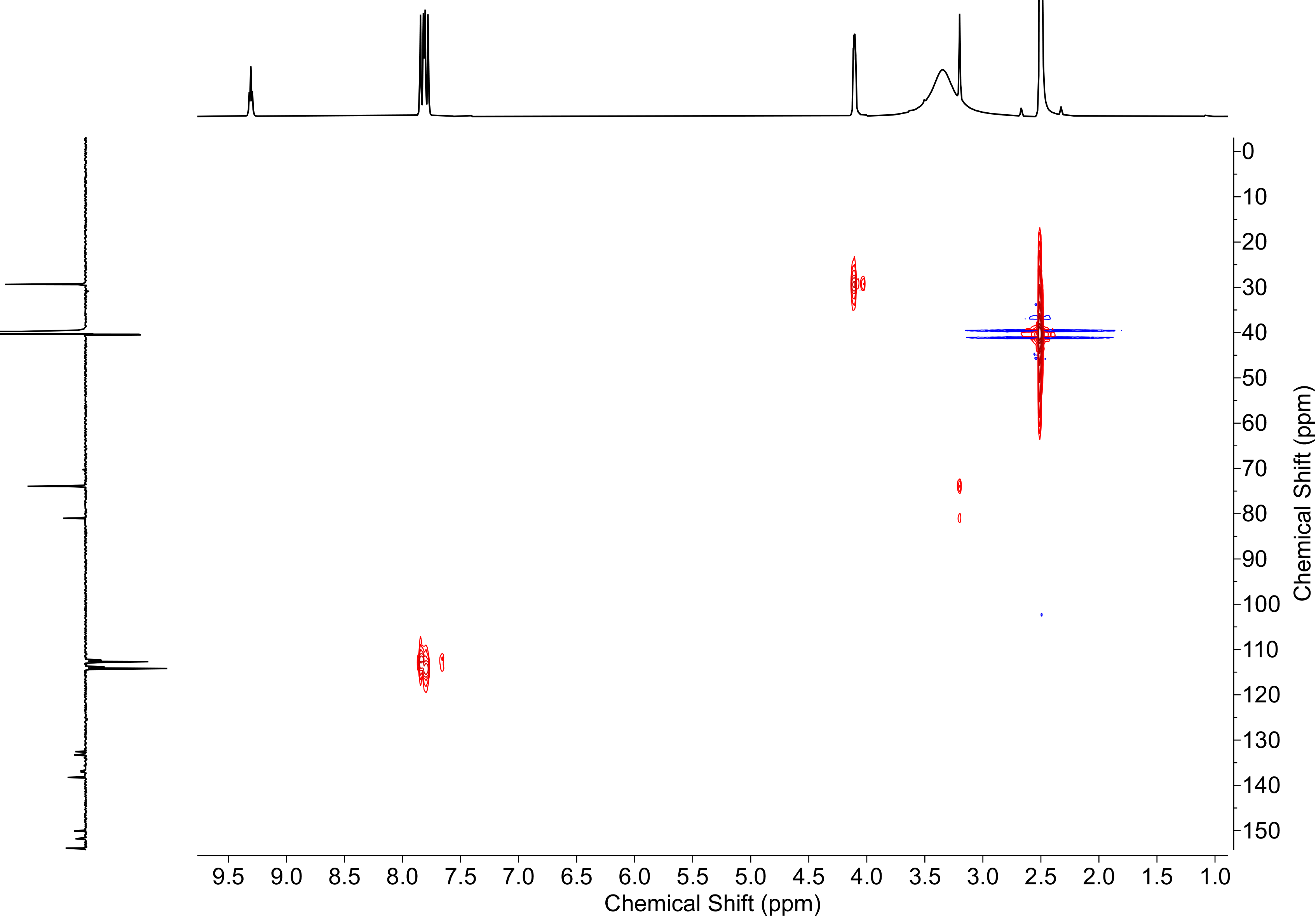


**Figure S5. HSQC NMR (DMSO-*d*_6_, 600 MHz, 298 K) of S3.**

**Synthesis of Alkyne-Ester Photoswitch** **1**.

In a 50 mL round bottomed flask, **S3** (200 mg, 527 µmol) was added followed by addition of MeOH as solvent and 2-3 drops of conc. H_2_SO_4_ (cat.). The reaction mixture was refluxed for 12 h then cooled, and the MeOH was removed *in vacuo* followed by extraction in EtOAc against NaCl_(sat)_ and H_2_O. The organic layer was dried on Na_2_SO_4_ followed by evaporation under reduced pressure to give a brown solid as product (195 mg, 496 µmol, 94%). The product was used without further purification. ^1^H NMR (400 MHz, DMSO) δ: 9.31 (t, *J* = 5.5 Hz, 1H_c_), 7.91 – 7.80 (m, 4H_d_), 4.11 (dd, *J* = 5.5, 2.6 Hz, 2Hb), 3.93 (s, 3H_e_), 3.21 (t, *J* = 2.5 Hz, 1H_a_). ^13^C NMR (151 MHz, DMSO) δ: 163.62, 162.63, 155.22 (d, *J* = 24.9 Hz), 153.50 (d, *J* = 24.3 Hz), 138.01, 131.99, 112.38, 80.52, 73.51, 53.12. ﻿^19^F NMR (376 MHz, DMSO) δ -119.76 (d, J = 10.0 Hz), -119.95 (d, J = 9.1 Hz). **HRMS**: m/z C_18_H_11_F_4_N_3_O_3_, observed 393.0749 (calc. 393.0737). *∆* = + 3 ppm.

**
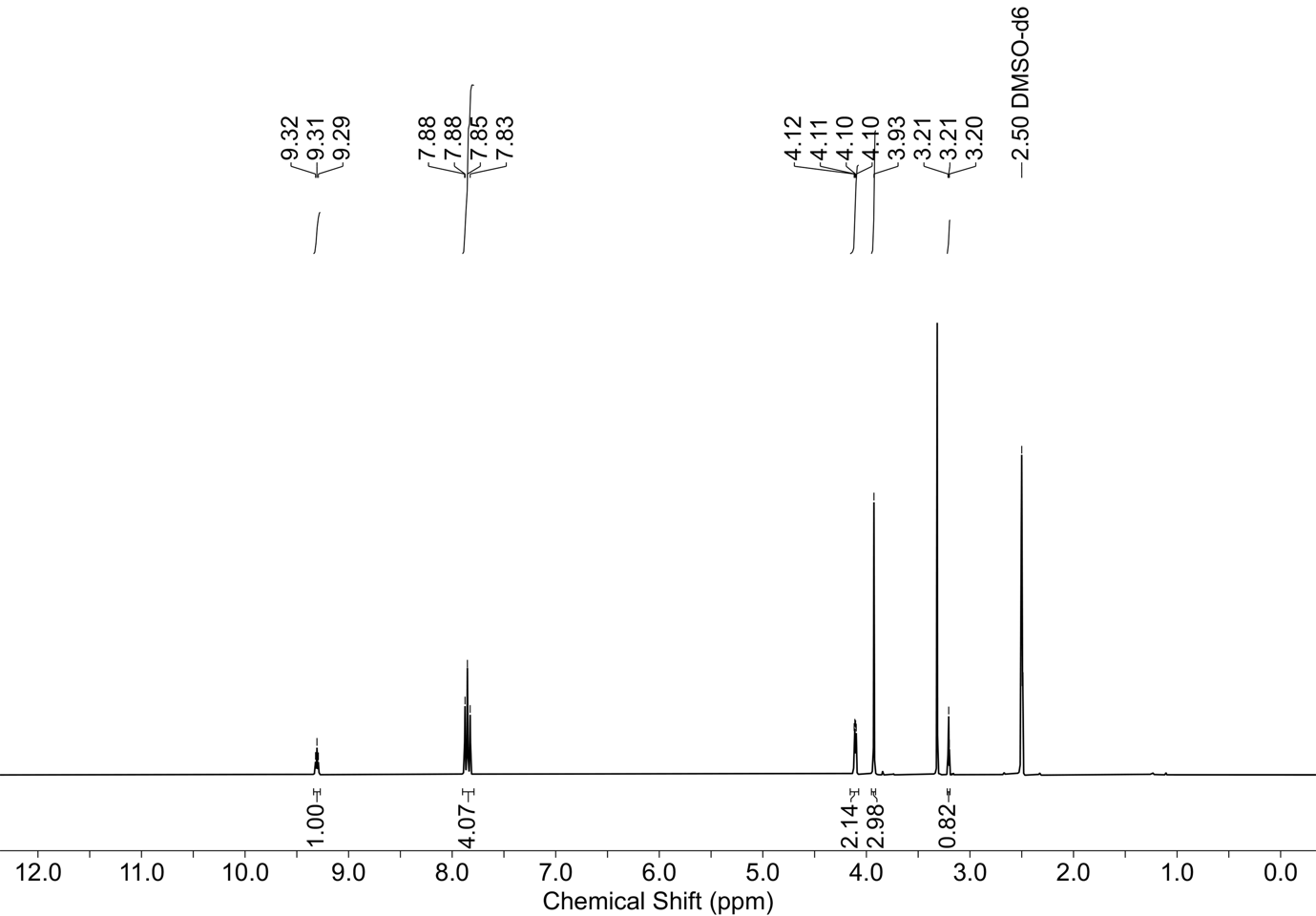
Figure S6. ^1^H NMR (DMSO-*d*_6_, 400 MHz, 298 K) of 1.**


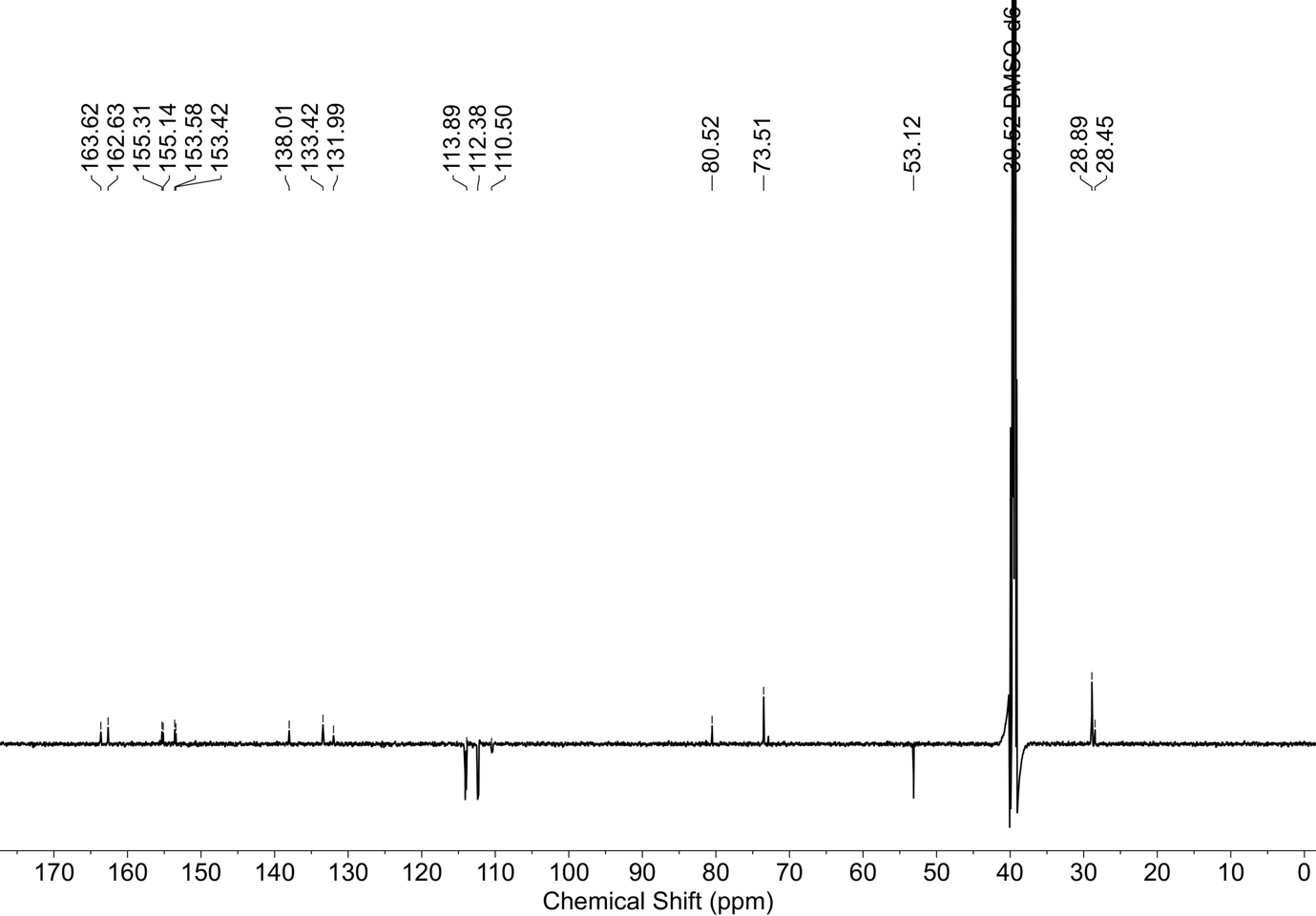


**Figure S7. J MOD NMR (DMSO-*d*_6_, 600 MHz, 298 K) of 1.**

**Figure S8. ^19^F NMR (DMSO-*d*_6_, 376 MHz, 298 K) of
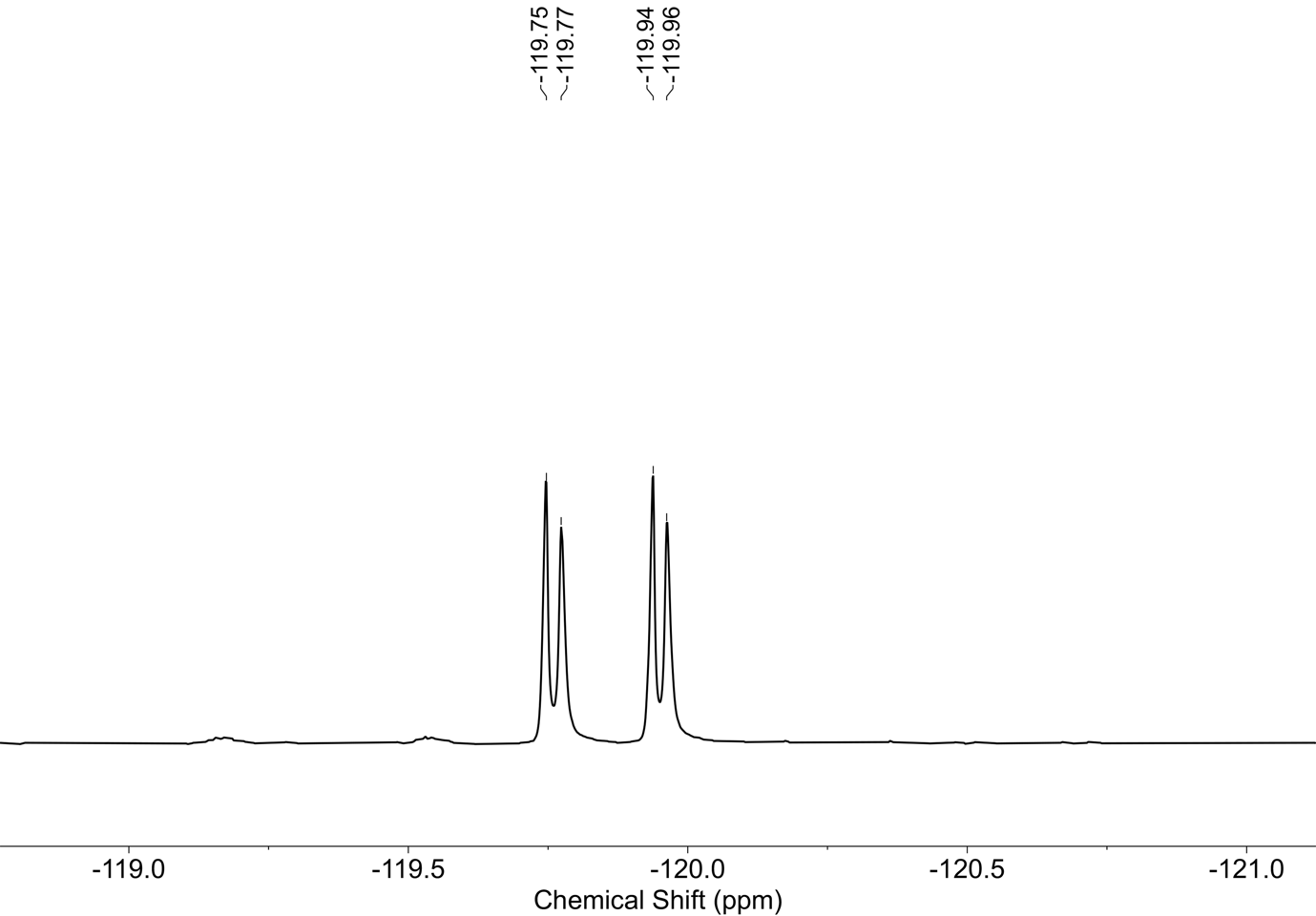
1.**

**
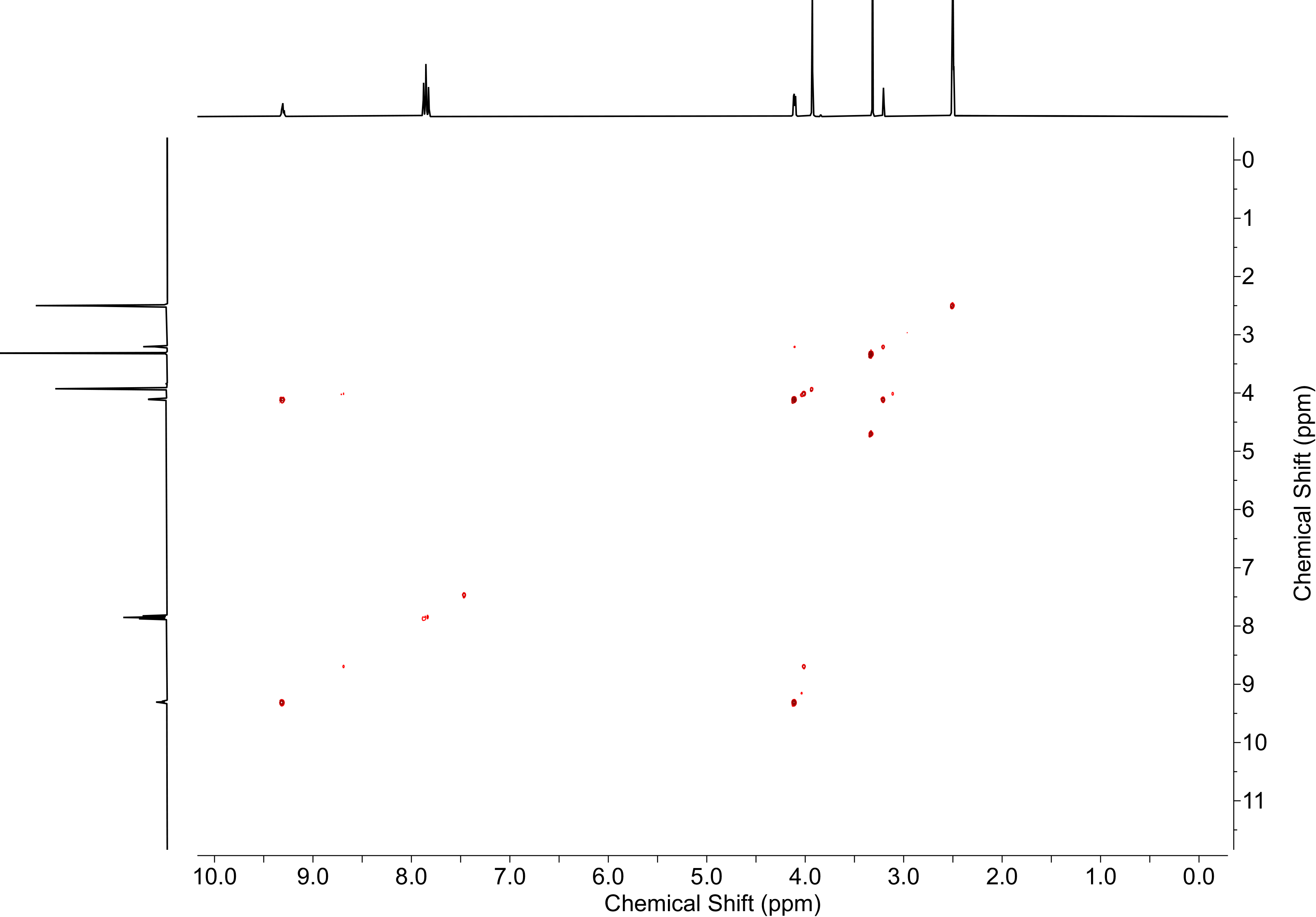
**

**Figure S9. COSY NMR (DMSO-*d*_6_, 600 MHz, 298 K) of 1.**

**
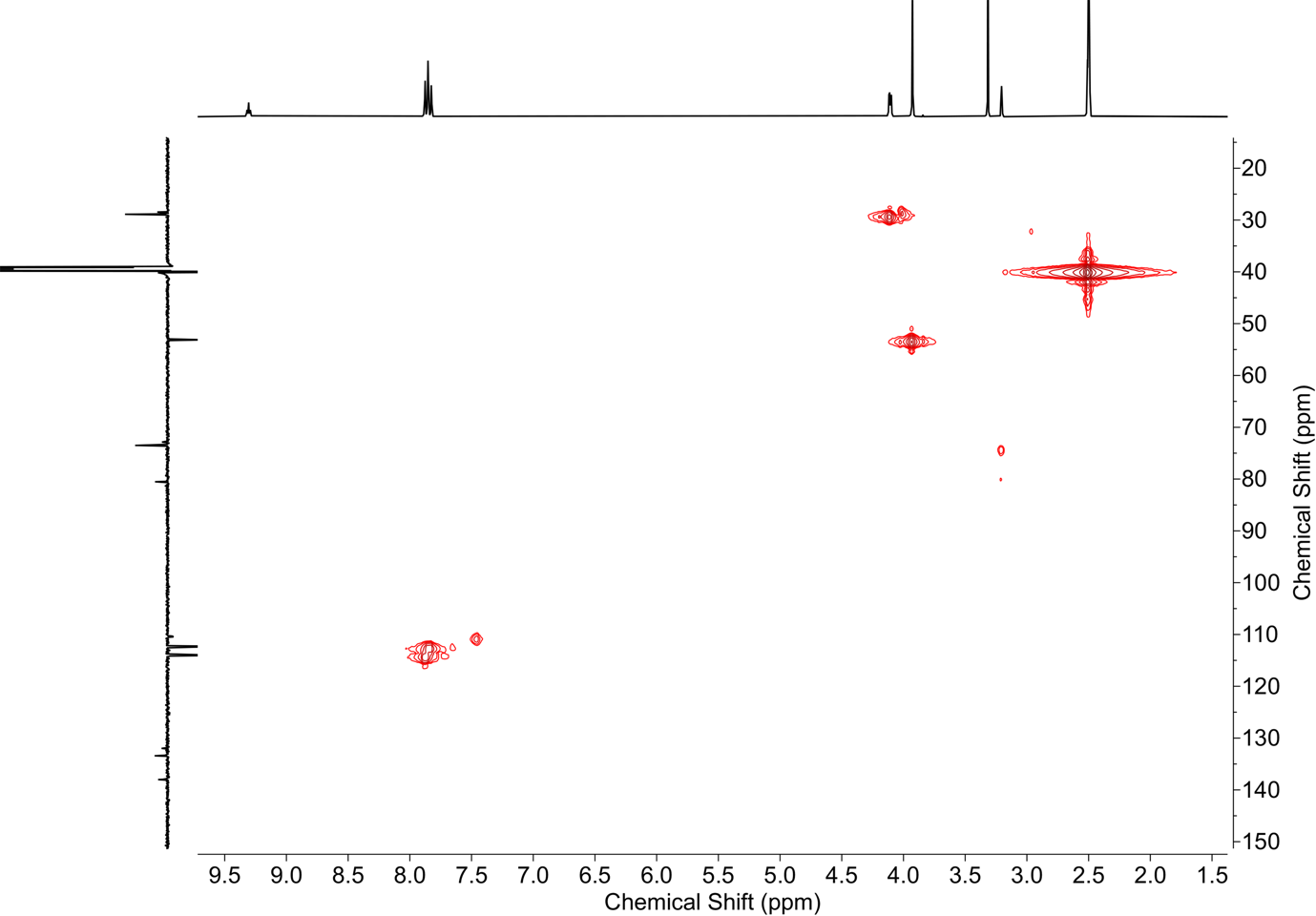
**

**Figure S10. HSQC NMR (DMSO-*d*_6_, 600 MHz, 298 K) of 1.**

**Synthesis of Pillar[5]arene appended tetrafluoroazobenzene Photoswitch** **3**.

In a 5 mL microwave vial under nitrogen atmosphere was added **1** (14 mg, 11 µmol, 1 eq.) and **2** (51 mg, 129 µmol, 12 eq.) in anhydrous CHCl_3_. This was followed by addition of tetrakis(acetonitrile)copper(I) tetrafluoroborate (7.0 mg, 32 µmol, 3 eq.) and a stoichiometric amount of TBTA (28 mg, 32 µmol, 3 eq.). Finally, DIPEA (18 µL, 108 µmol, 10 eq.) was added, and reaction flask was sealed and allowed to stir at 45 ºC for 24 hours. A 0.1 M EDTA solution (made in 9:1 H_2_O:NH_3_) (3 mL) was then added and the reaction mixture was stirred vigorously for 15 minutes, followed by extraction with CH_2_Cl_2_ (10 mL). The organic layer was dried over anhydrous Na_2_SO_4_ and concentrated followed by size exclusion chromatography using pre-swollen SX1 beads with CH_2_Cl_2_ as eluent. The eluted compound was dried under reduced pressure to give a red solid powder as the crude product. This crude ester product was used without further purification, and after drying was added to a 25 mL flask product followed by addition of THF (2.0 mL) and H_2_O (1.0 mL). To this solution was added lithium hydroxide monohydrate (2.8 mg, 3.8 µmol, 30 eq.). The mixture was stirred at 25 ºC for 12 h and then poured into water (50 mL). After acidifying with diluted aqueous HCl solution (3 M, 5-6 drops), the precipitate was collected by filtration and washed with H_2_O to give compound as red solid powder (18 mg, 3.8 µmol, 34% overall). The product was washed with pentane and dried under high vacuum for 12 h. ^1^H NMR (400 MHz, DMSO) δ: δ: 9.34 (br, 10 H_f_), 8.17 (br, 10H, H_d_), 7.65 (br, 40H, H_g/g’_), 6.61 (s, 10H, H_a_), 4.79 (s, 20 H, H_e_), 4.52 – 4.08 (m, 40H, H_c_), 3.08 (app s, 10H, H_b_).^13^C NMR (151 MHz, DMSO) δ: 164.90, 163.11, 153.81, 149.04, 145.19, 138.21, 135.21, 133.31, 132.08, 128.75, 114.13, 112.30, 67.60, 50.08, 35.52. ^19^F NMR (376 MHz, DMSO) δ: -119.67, -119.95, -120.11.

**3-*Z*** isomer: ﻿^1^H NMR (400 MHz, DMSO) δ: 9.36 – 8.78 (m, 10H, H_f_), 8.13 (app s, 10H, H_d_), 7.60 (app d, J = 33.1 Hz, likely isomeric peak corresponding to H_g/g’_ of *E*-state), 7.61 – 7.35 (m, 40H, H_g_), 6.65 (br, 10H, H_a_), 4.78 (s, 20H, H_e_), 4.53 – 4.08 (m, 40H, H_c_), 3.07 (app s, 10H, H_b_). ^13^C NMR (151 MHz, DMSO) δ: 164.81, 163.11, 155.60, 153.87, 151.72, 145.07, 137.31, 134.23, 132.87, 123.81, 113.99 (d, *J* = 24.3 Hz), 112.21, 110.76, 67.61, 50.10, 35.49. ^19^F NMR (376 MHz, DMSO) δ: -119.45, -119.56 (d, *J* = 29.5 Hz), -119.81, -120.06.

**
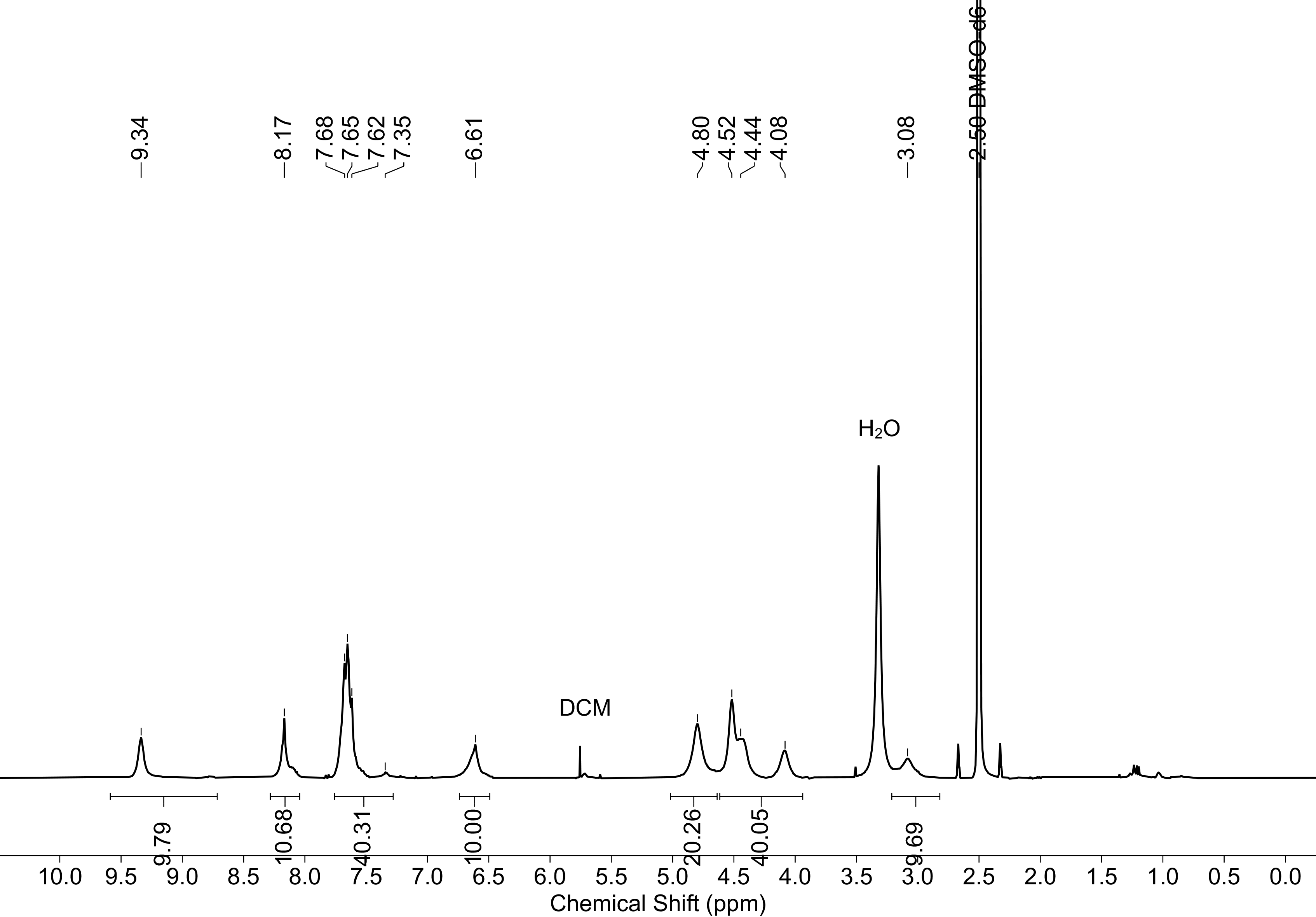
Figure S11. ^1^H NMR (DMSO-*d*_6_, 600 MHz, 298 K) of 3-*E*.**

**
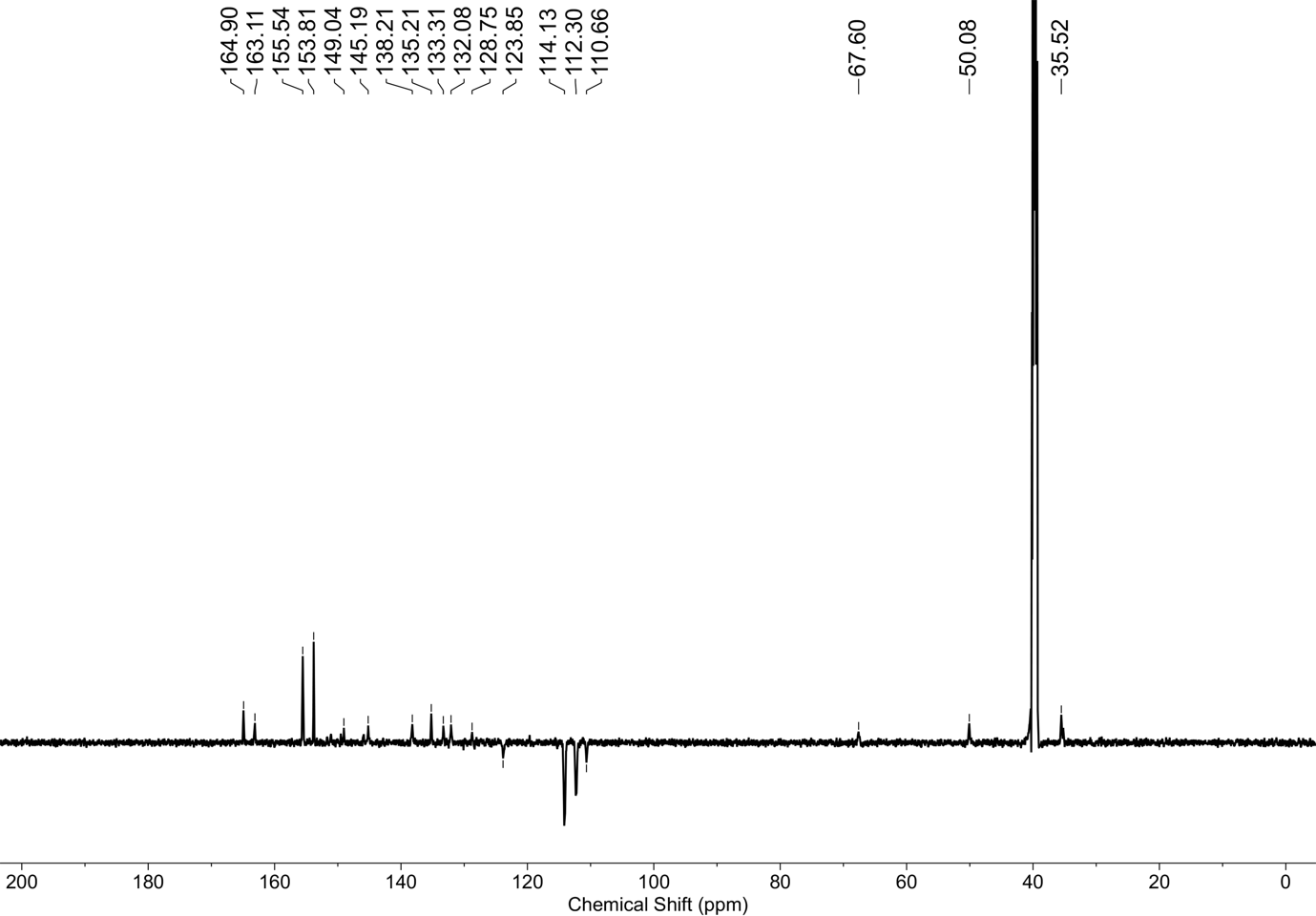
Figure S12. J MOD NMR (DMSO-*d*_6_, 600MHz, 298 K) of 3-*E.***


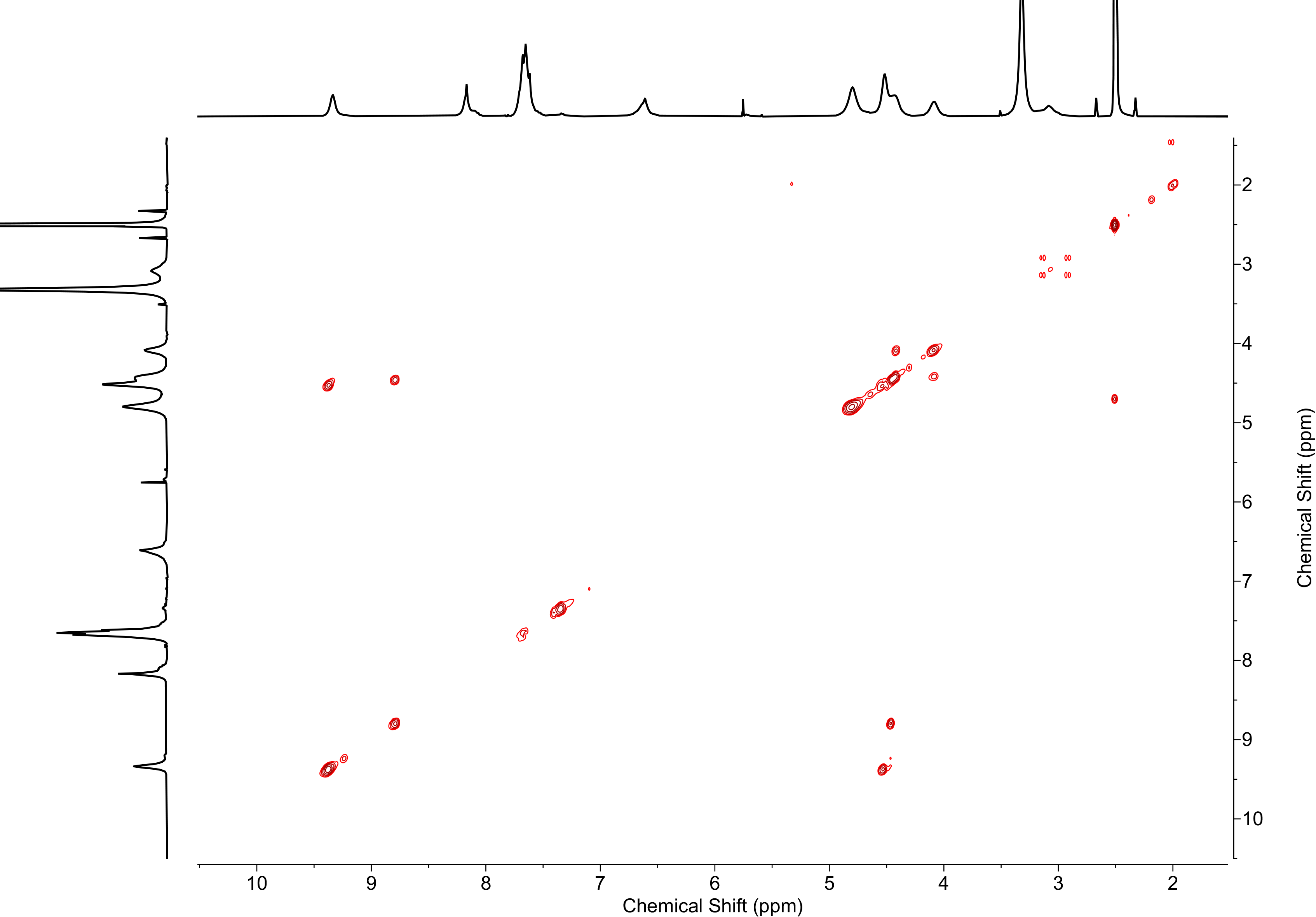


**Figure S13. COSY NMR (DMSO-*d*_6_, 600 MHz, 298 K) of 3-*E.***

-
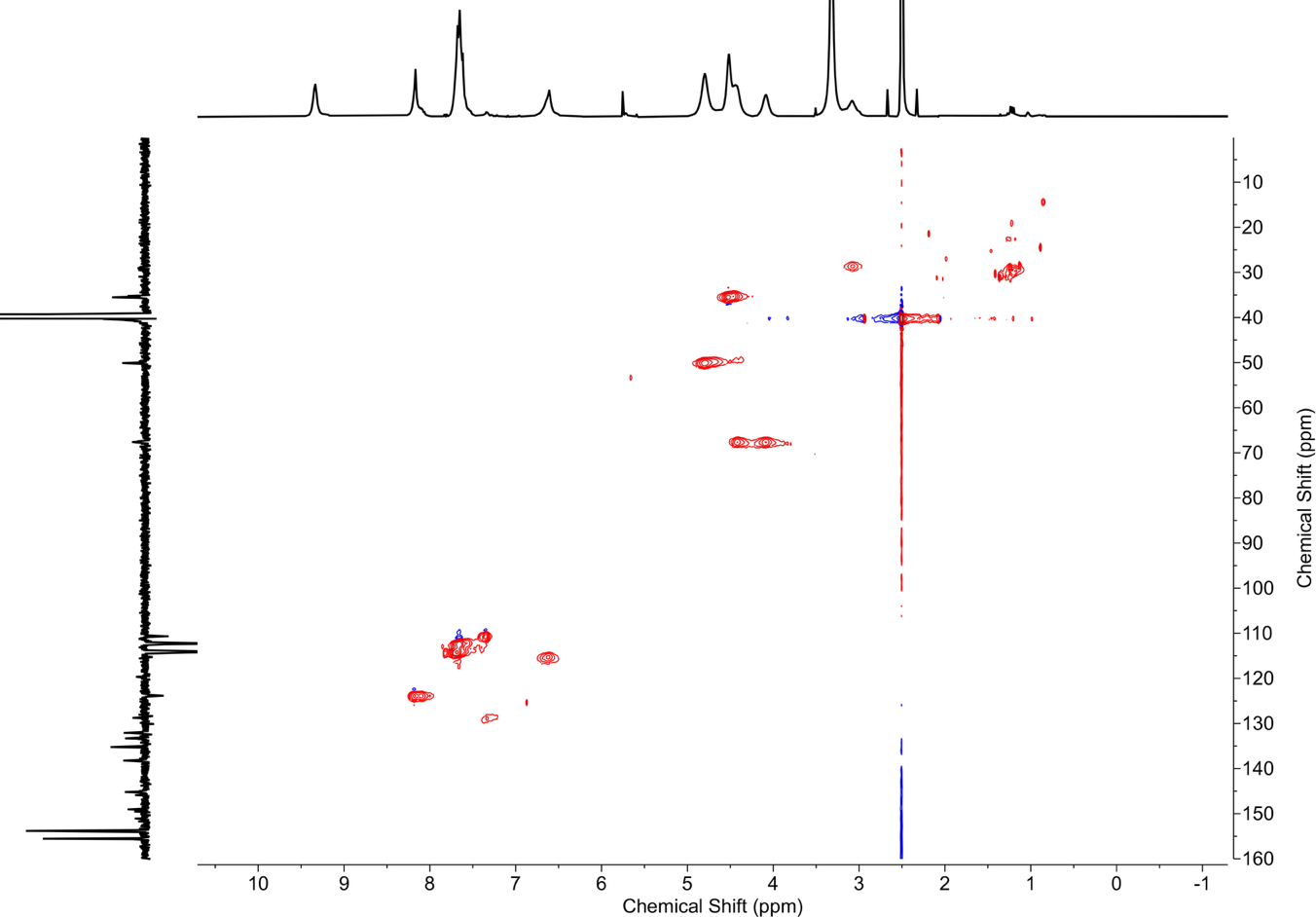


**Figure S14. HSQC NMR (DMSO-*d*_6_, 600 MHz, 298 K) of 3-*E.***

**
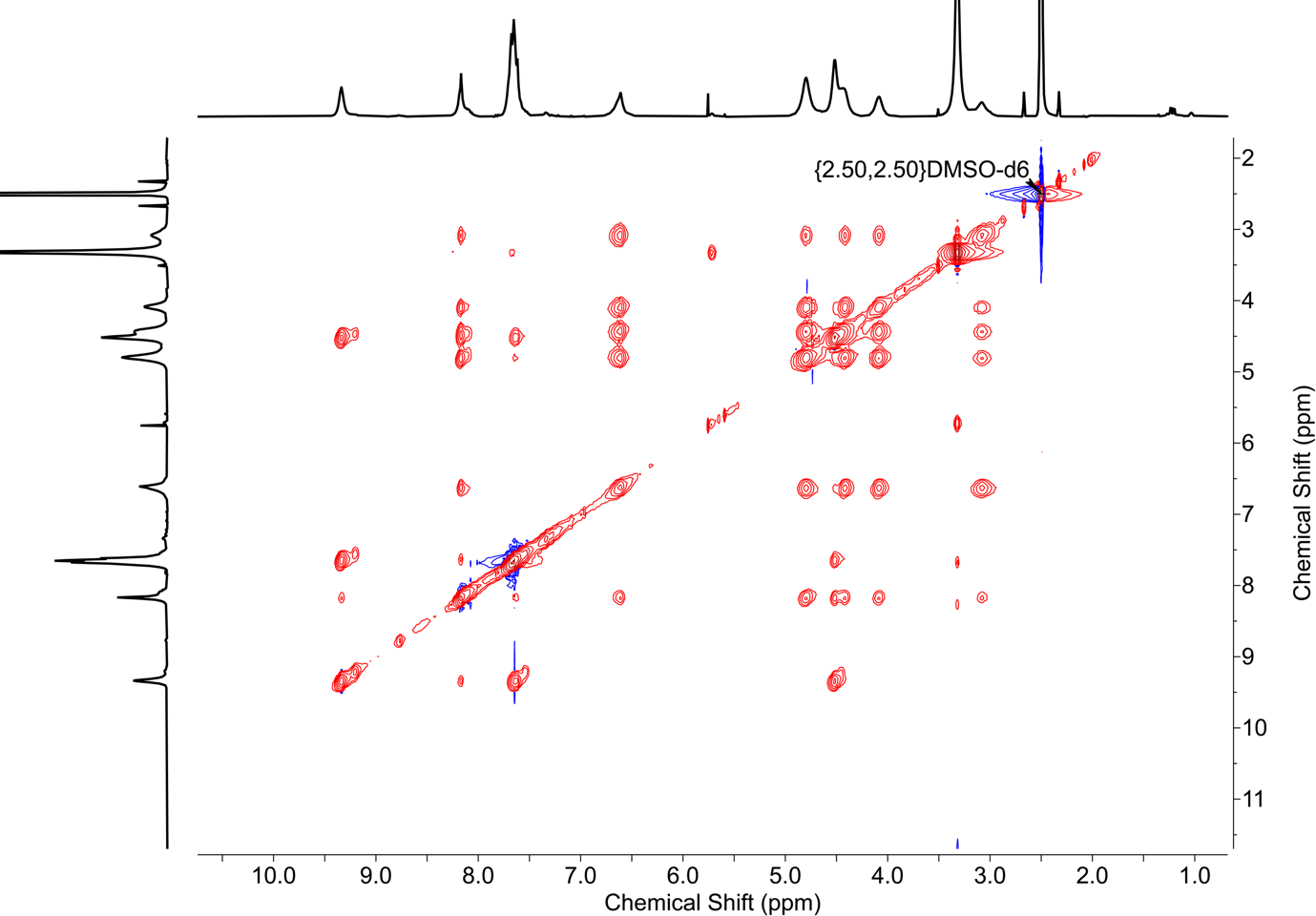
**

**Figure S15. NOESY NMR (DMSO-*d*_6_, 600 MHz, 298 K) of 3-*E.***

**
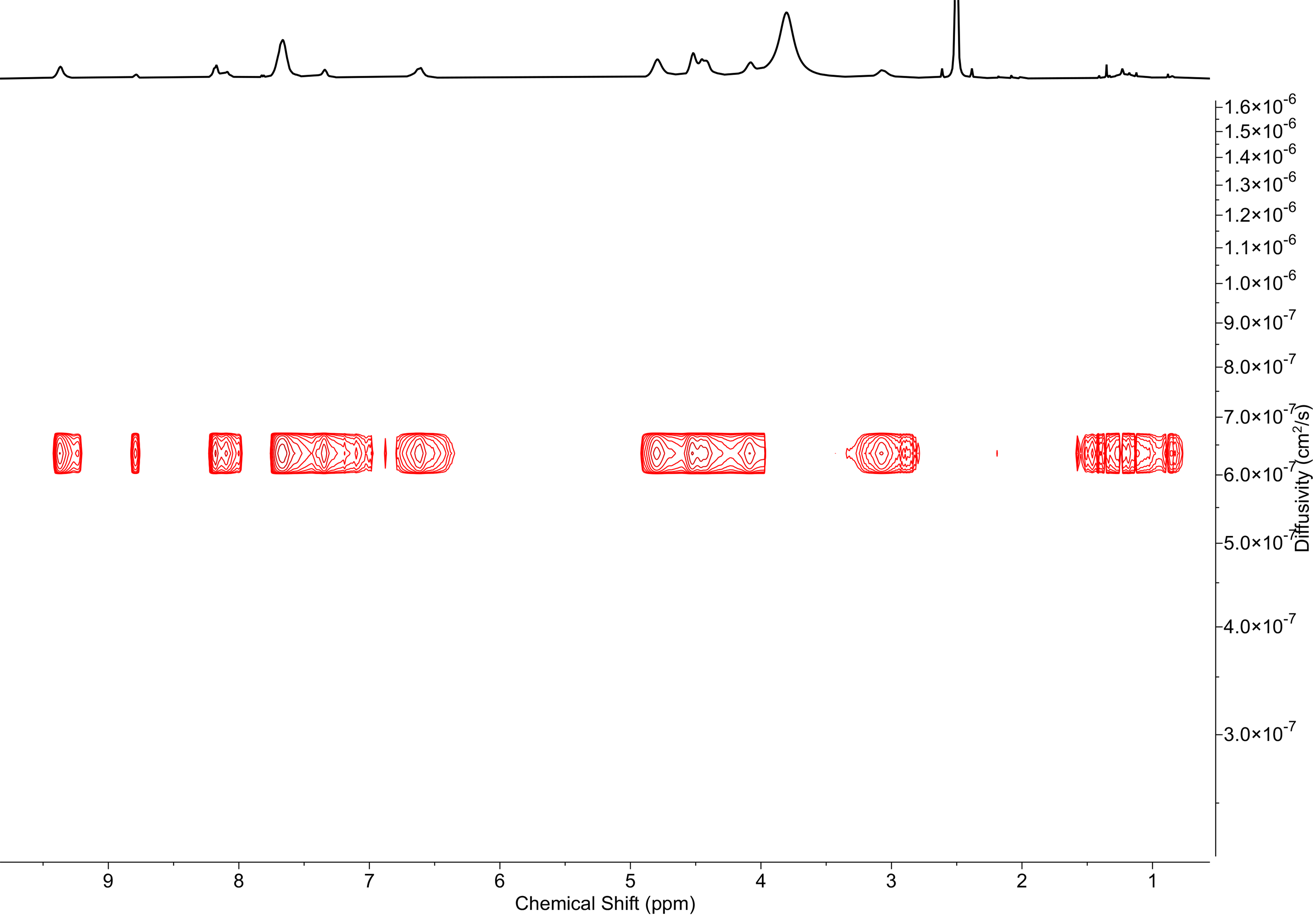
**

**Figure S16. DOSY NMR (DMSO-*d*_6_, 600 MHz, 298 K) of 3-*E*** **corresponding to the** hydrodynamic radius of 1.35 nm, and diffusion coefficient of 6.158 x 10^-7^ cm^2^ s^-1^.

**
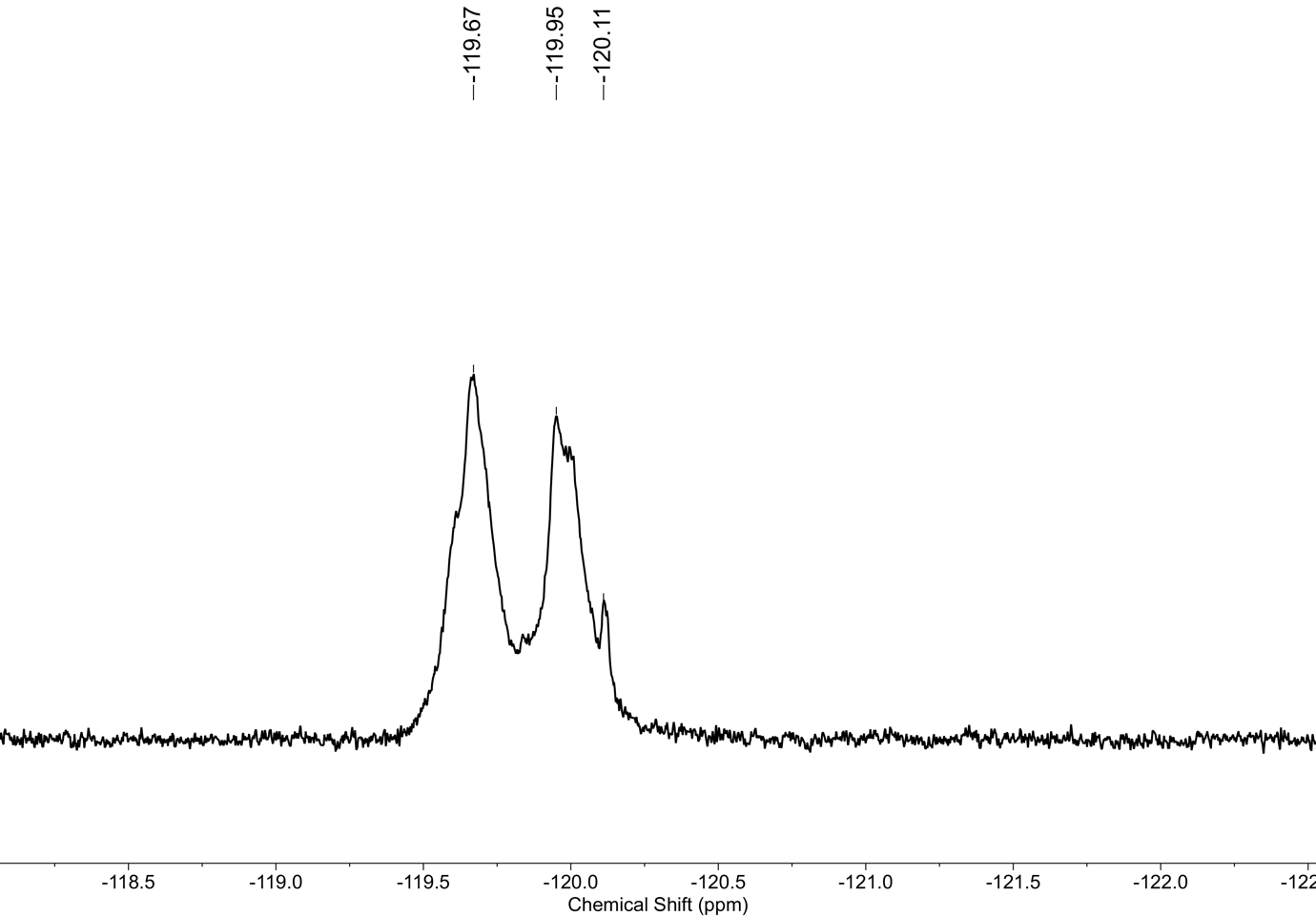
Figure S17. ^19^F NMR (DMSO-*d*_6_, 376 MHz, 298 K) of 3-*E.***

**

**

﻿﻿**Figure S18. ^1^H NMR (DMSO-*d*_6_, 600 MHz, 298 K) of 3-*Z.***

**
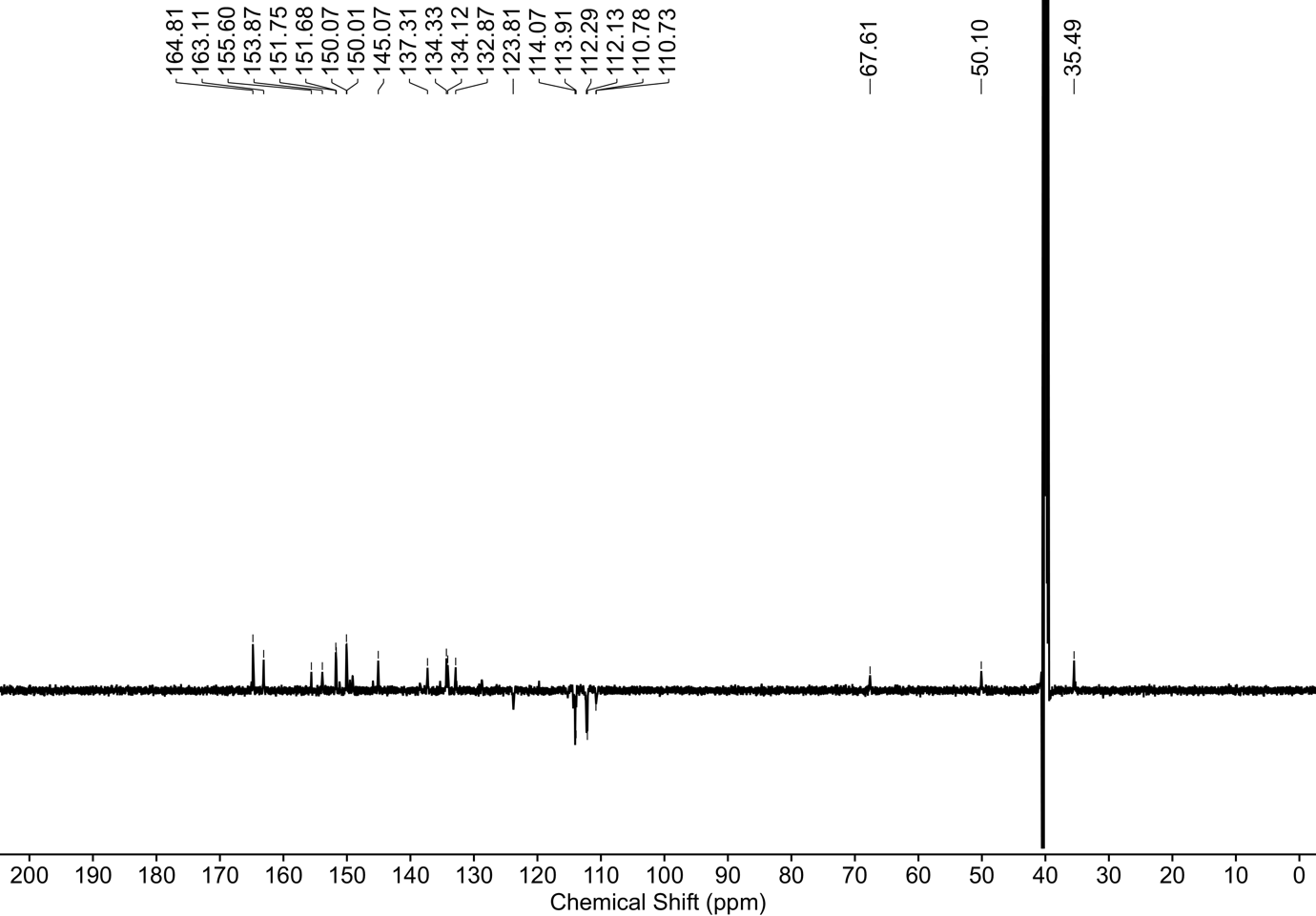
Figure S19. J MOD NMR (DMSO-*d*_6_, 600MHz, 298 K) of 3-*Z.***

**
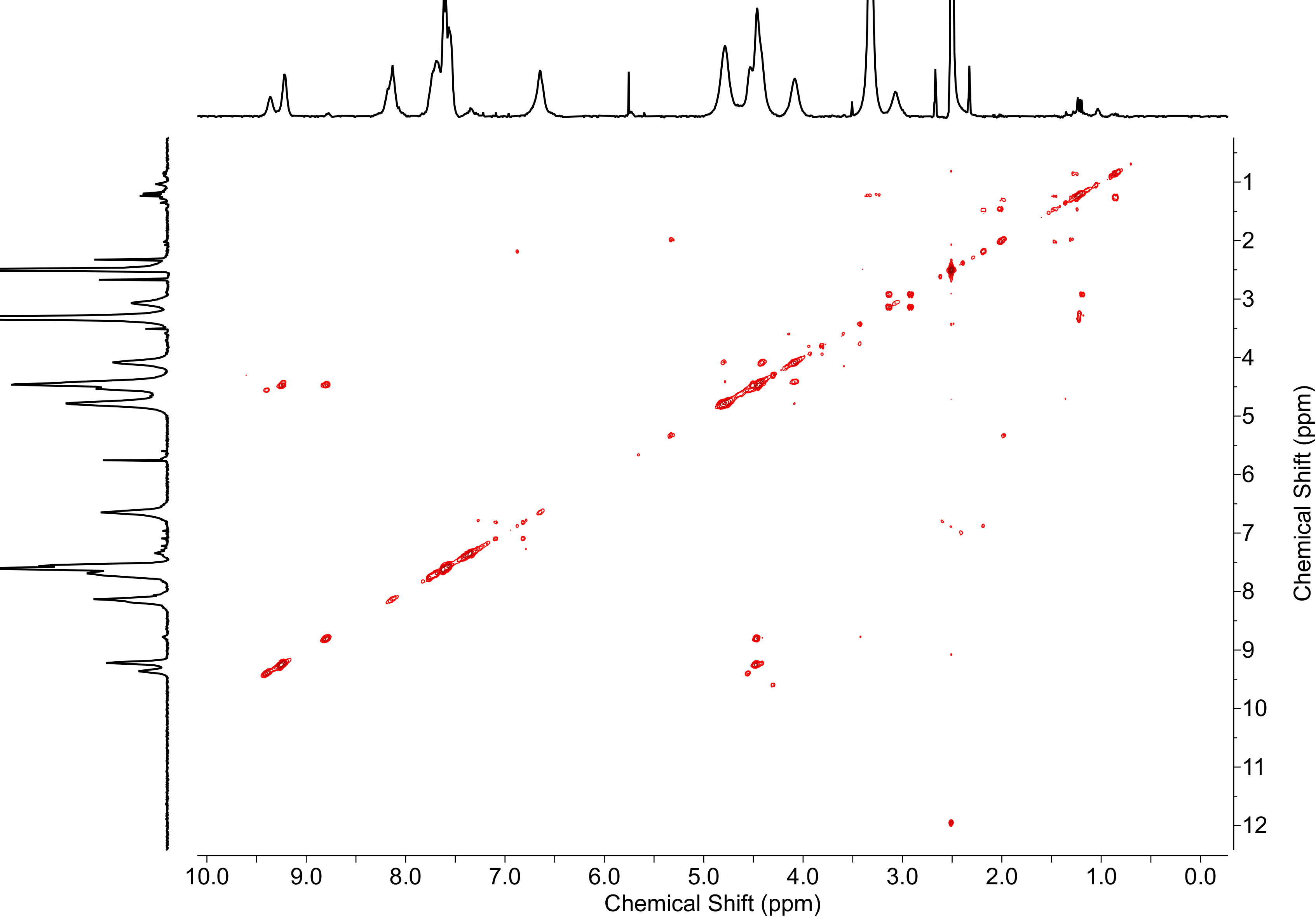
**

**Figure S20. COSY NMR (DMSO-*d*_6_, 600 MHz, 298 K) of 3-*Z.***

**
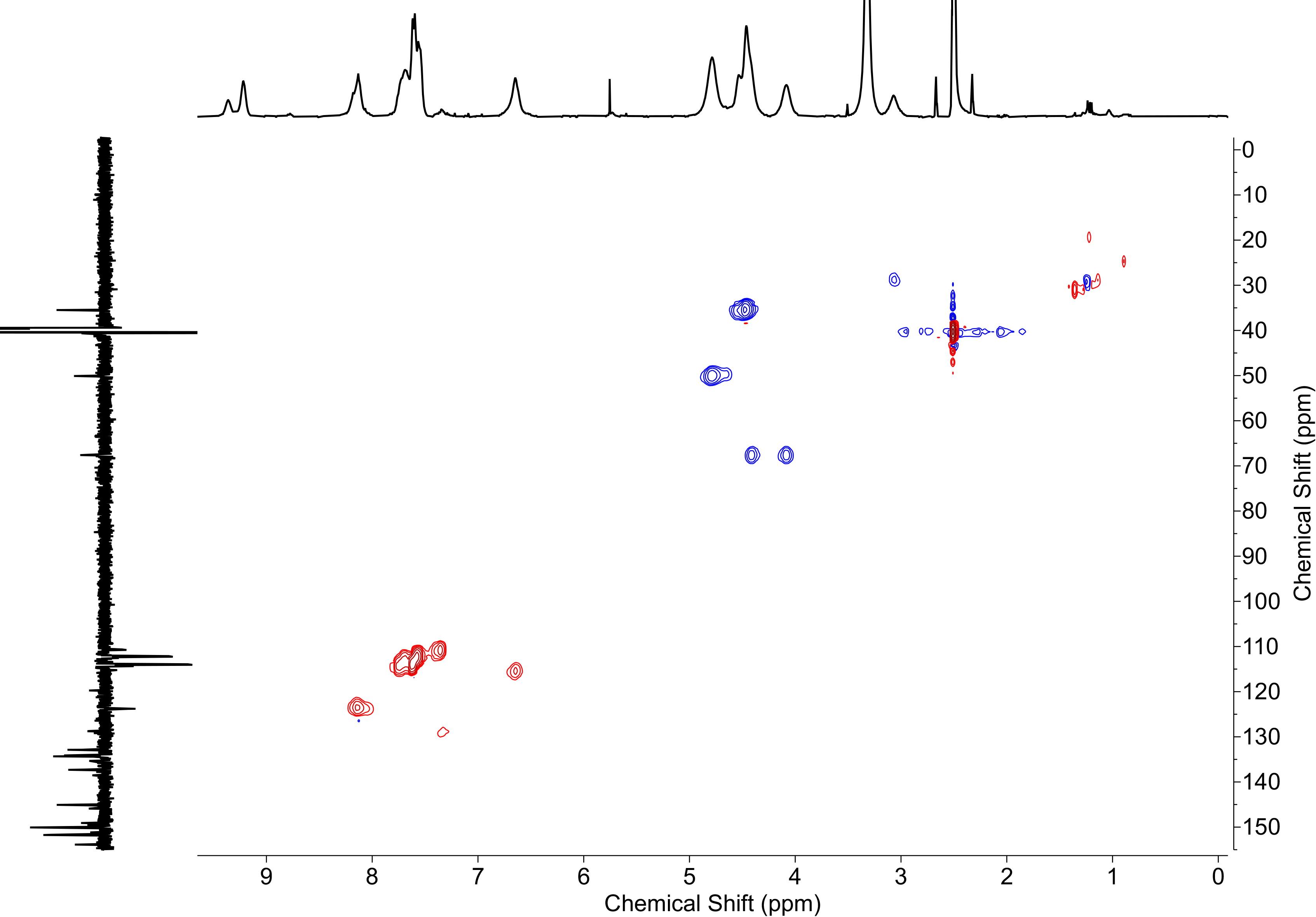
**

**Figure S21. HSQC NMR (DMSO-*d*_6_, 600 MHz, 298 K) of 3-*Z.***

***

***

**Figure S22. NOESY NMR (DMSO-*d*_6_, 600 MHz, 298 K) of 3-*Z.***


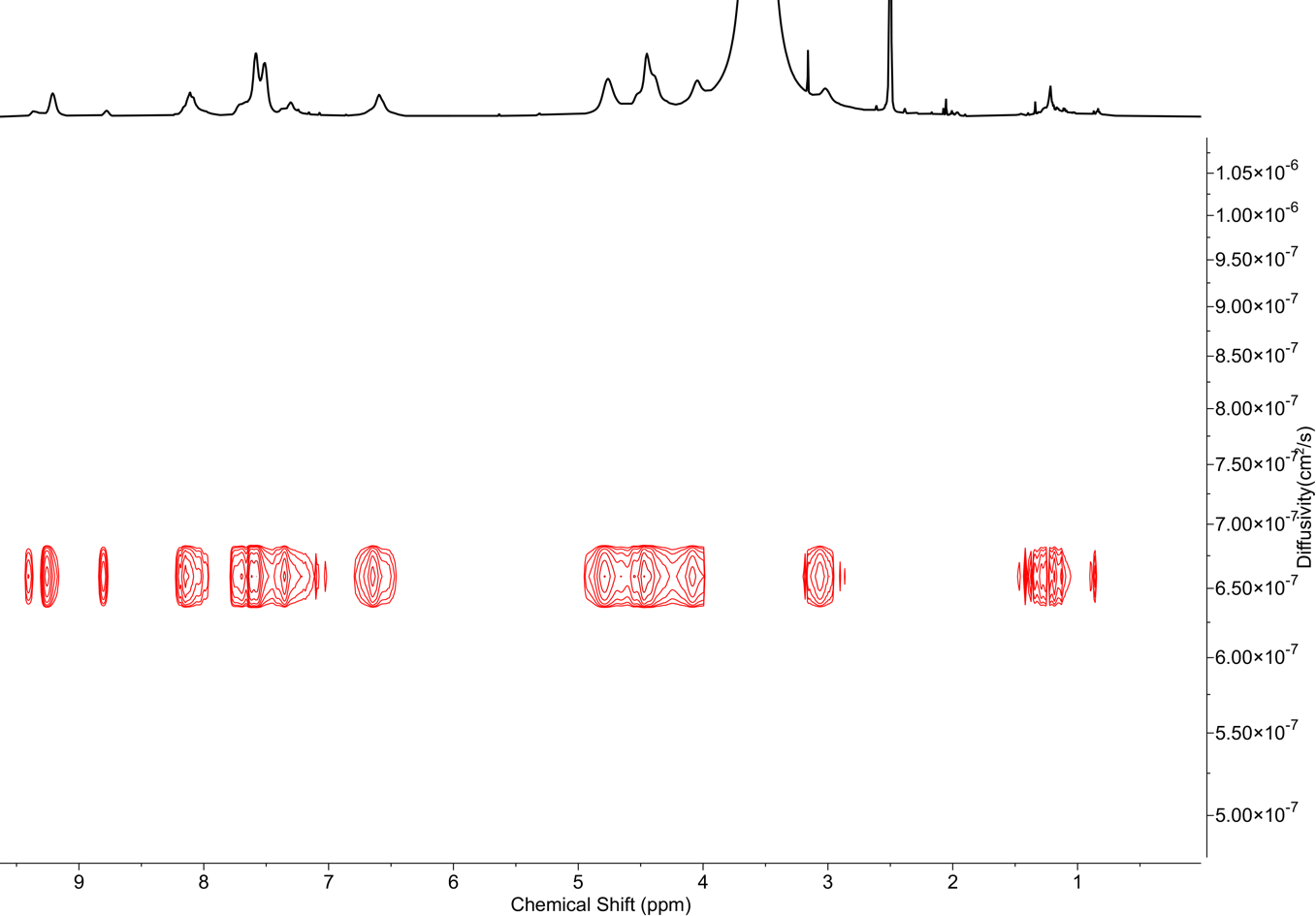
**Figure S23. DOSY NMR (DMSO-*d*_6_, 600 MHz, 298 K) of 3-*Z* corresponding to the** hydrodynamic radii of 1.41 nm, and diffusion coefficient 6.649 x 10^-7^ cm^2^ s^-1^.

**
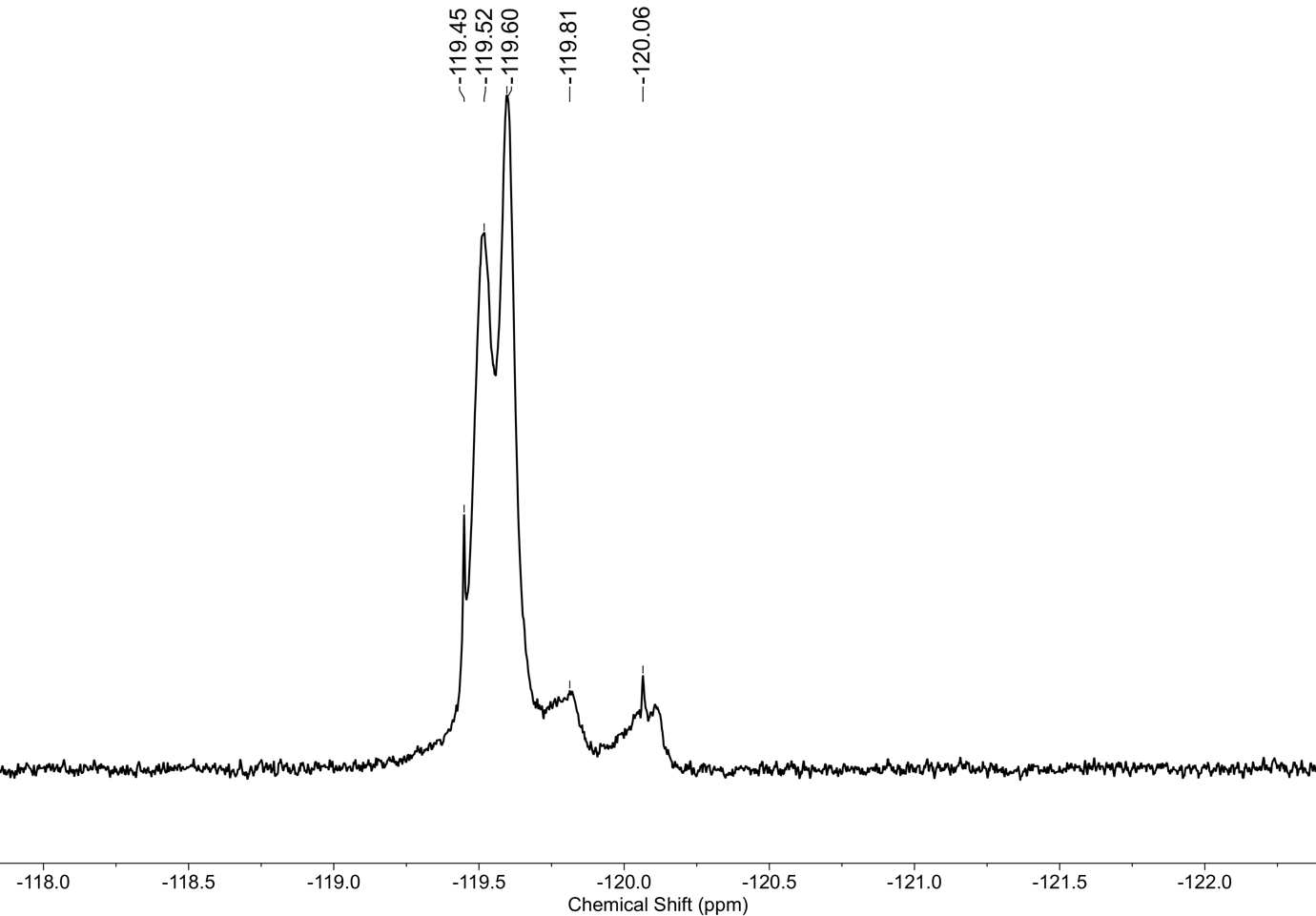
**

**Figure S24. ^19^F NMR (DMSO- *d*_6_, 376 MHz, 298 K) of 3-*Z.***

**
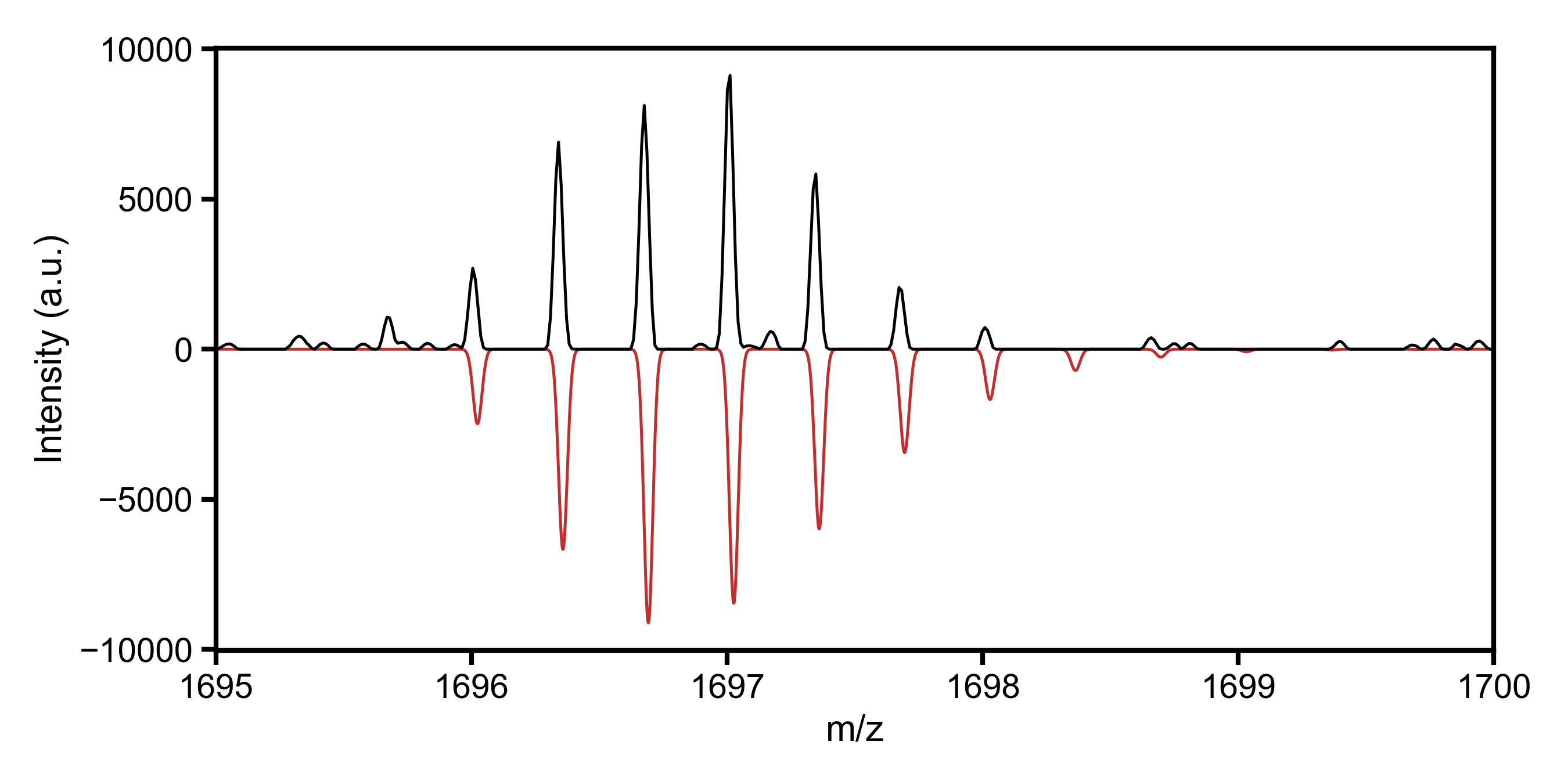
**

**Figure S25. HRMS (top) and calculated (bottom) isotope patterns of [3]^3−^ with molecular formula** C_225_H_147_F_40_N_60_O_40_^3-^.

# Photoisomerization Experiments^S1^

**
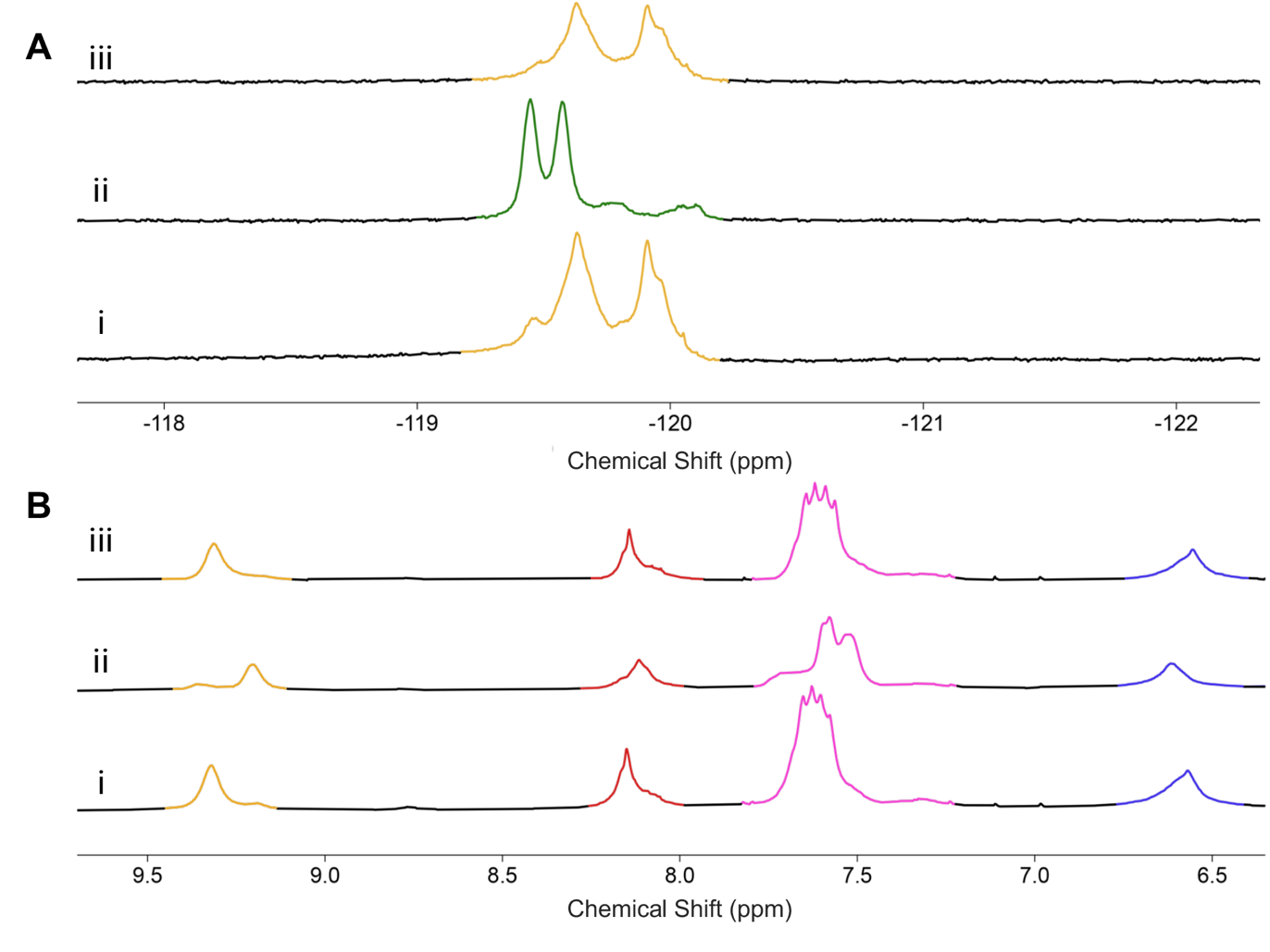
**Irradiation of liquid samples was carried out using Thorlabs high-power mounted LEDs (models M530L4 - 530 nm, 370 mW (Min) Mounted LED, 1000 mA and M415L4 - 415 nm, 1310 mW (Min) Mounted LED, 1500 mA) using an in-house custom-built set-up with optical components supplied by Thorlabs. When irradiating small vials, NMR tubes and cuvettes, a Thorlabs cuvette holder (CVH100/M) equipped with the mounted LEDs was used. Samples were irradiated until the photo-stationary state was reached, as confirmed by ^1^H NMR, ^19^F NMR, DOSY NMR and UV-vis experiments.

**Figure S26.** A) ^19^F NMR of **3** in DMSO-***d*_6_** (298 K, 376 MHz) and (B) ^1^H NMR of **3** in DMSO-***d*_6_** (298 K, 600 MHz) (i) as synthesized, (ii) after 5 minutes of 530 nm irradiation and (ii) after 5 minutes of 415 nm irradiation. (C) UV-Vis absorbance spectra of **3** (25 µM) after synthesis, 530 nm irradiation and 415 nm irradiation. (D) Zoomed in view of 400 nm - 600 nm region.

The assignment of the detailed structure of the ^1^9F NMR is challenging. We observe two different sets of signals, both by shift and spectral appearance, upon switching which act as fingerprints for the two isomeric states. We note that the spectra of **3**-*Z* is simpler than **3**-*E* – we see two major peaks, which we attribute to the two ^19^F environments present. We observe two minor peaks assigned to residual *E* isomer; integral ratios are consistent with ^1^H integrations of residual *E*. The spectra of **3**-*E* is more complex, with two main peaks, but significant broadening. We attribute this to the partial stacking of the azobenzene units, consistent with our computational modelling, which generate a range of similar, but distinct, environments for the ^19^F. This effect is absent in our models, and spectra, of **3**-*Z*.


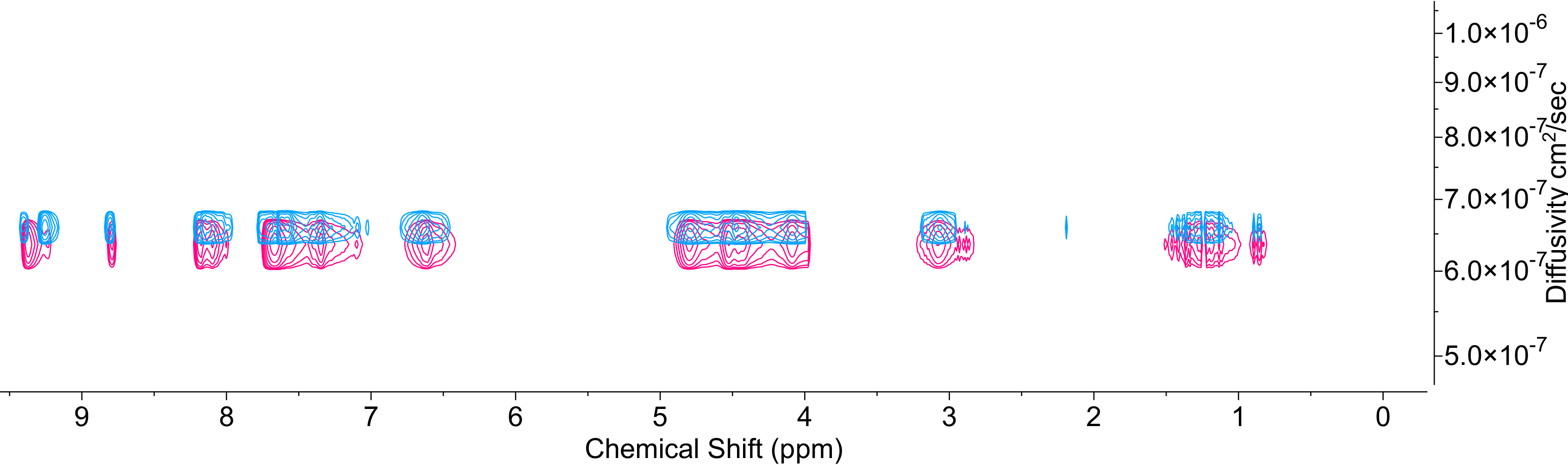


**Figure S27.** DOSY NMR of **3** in DMSO***-d*_6_**, recorded after irradiation with 415 nm (blue) or 530 nm (pink) corresponding to hydrodynamic radii of 1.35 nm and 1.42 nm respectively, suggesting an efficient isomerization.

**Photofatigue Studies:** The photo fatigue studies were carried out by switching **3** from *E* to Z and vice-versa for 10 repetitive cycles and measuring the absorbance at 323 nm. The data shows minimal fatigue suggesting that **3** behaves as a robust photoswitch.

**Figure S28. (A)** UV-Vis spectra of **3** (25 µM) in DMSO, measured after irradiation at 415 nm and 530 nm. (B) Switching cycles for **3** (25 μM) under alternating irradiation with 415 nm and 530 nm light.

# Ion Transport Assays

1. **Chloride transport activity across POPC‒LUVs**⊃**lucigenin vesicles**:^S3-S5^

**Buffer and stock solution preparation**: Phosphate buffer was prepared by dissolving an appropriate amount of solid Na_2_PO_4_ and NaHPO_4_ and a NaNO_3_ salt in milliQ water to get 10 mM phosphate buffer and 225 mM NaNO_3_ salt, respectively. Subsequently, the pH was adjusted to 7.0 by adding 0.5 M NaOH solution. The stock solution of **3** was prepared in HPLC grade DMF solution for the studies.

**Preparation of POPC‒LUVs**⊃**lucigenin**: In a clean and dry small (10 mL) round bottom flask, 1 mL of 1-palmitoyl-2-oleoyl-sn-glycero-3-phosphocholine (POPC, 25 mg/mL stock in CHCl_3_) was added. The solution was dried by a flow of nitrogen with continuous rotation to form a thin transparent film of POPC. The transparent film was kept in a high vacuum at RT for 4 h to remove all traces of CHCl_3_. The resulting film was hydrated with 1 mL buffer solution (1 mM lucigenin, 10 mM phosphate buffer, and 225 mM NaNO_3_, pH = 7.0), and the resulting suspension was vortexed at 10 min intervals for 1 h. This hydrated suspension was subjected to 21 cycles of freeze-thaw (liquid N_2_, 55 °C) followed by extrusion through 200 nm pore size containing polycarbonate membrane 21 times (this must be an odd number), to form a uniform distribution of vesicles with an average 200 nm diameter. Extravesicular dye was removed by gel filtration (using Sephadex G-50) with buffer solution (10 mM phosphate buffer and 225mM NaNO_3_, pH = 7.0), and diluted to 4 mL to get POPC‒LUVs⊃lucigenin. Final conditions: ~ 5 mM POPC; Inside: 1 mM lucigenin, 10 mM phosphate buffer, 225 mM NaNO_3_, pH = 7.0; Outside: 10 mM phosphate buffer, 225 mM NaNO_3_, pH = 7.0.

**Comparison of ion transport activity in POPC‒LUVs⊃lucigenin**: In a clean and dry fluorescence cuvette, 1950 µL of buffer solution (10 mM phosphate buffer, 225 mM NaNO_3_, pH = 7.0), 50 µL POPC‒LUVs**⊃**lucigenin was added. This suspension was placed with slow stirring in a Fluorometer equipped with a magnetic stirrer (at *t* = 0 s). The fluorescence intensity of lucigenin was monitored at λ_em_ = 535 nm (λ_ex_ = 450 nm) over time. A chloride gradient between the intra- and extravesicular compartments was created by the addition of 2.0 M NaCl (33.3 µL) at *t* = 20 s, followed by the addition of channel-forming molecule **3** (after isomerization in solution for respective isomer) at *t* = 100 s. Finally, vesicles were lyzed by adding 10% Triton X‒100 (25 μL) at *t* = 300 s to eliminate the chloride gradient. The collected data was then normalized to the percentage change in fluorescence intensity using Equation S2.

**Figure S29.** (A) Schematic representation of ion transport activity across POPC‒LUVs**⊃**lucigenin vesicles, and (B) normalization window for same fluorescence kinetics experiment of ion transport.

The time axis underwent a synchronisation operation according to Equation S1:

*t* = *t_0_* ‒ 100 Equation S1

where, in synchronised data *t* = 0 s is the time of compound addition during the experiment, and *t* = 200 s is time of Triton X‒100 addition.

The time-dependent data were normalized to get relative fluorescence intensity using Equation S2

*Relative Fluorescence Intensity* = *F/F_0_*  Equation S2

where *F_0_* = Maximum fluorescence intensity, normally the first datapoint after the addition of channel forming molecule (at 0 s), *F* = fluorescence intensity at time *t*.

1. **Ion transporting activity studies across POPC‒LUVs**⊃**HPTS**^S6^

**Preparation of HEPES buffer and stock solutions**: The HEPES buffer (pH = 7.0) was prepared by dissolving an appropriate amount of solid HEPES (10 mM) and NaCl (100 mM) in milli-Q water. The pH was adjusted to 7.0 by the addition of aliquots from a NaOH solution (0.5 M). HPLC grade DMSO was used to prepare the stock solution for all the derivatives.

**Preparation of POPC‒LUVs⊃HPTS with NaCl**: In a dry and clean round bottom flux (10 mL), 1 mL of 1-palmitoyl-2-oleoyl-sn-glycero-3-phosphocholine (POPC, 25 mg/mL stock in CHCl_3_ was dried by a flow of nitrogen gas with continuous rotation to make a thin transparent film of POPC, followed by drying on high vacuum at RT for 4 h to remove traces of CHCl_3_. The dried thin film was then hydrated with 1 mL HEPES buffer (1 mM HPTS, 10 mM HEPES, 100 mM NaCl, pH = 7.0), and the resulting suspension was vortexed at regular intervals to form a uniform suspension. This hydrated suspension was subjected to 21 cycles of freeze and thaw (liquid N_2_ and 55 °C hot water bath) followed by extrusion through 200 nm polycarbonate membrane 25 times (this must be an odd number), to achieve uniform distribution of LUVs of an average 200 nm diameter. Finally, size exclusion chromatography using gel filtration Sephadex G-50 was carried out to remove the unentrapped extravesicular HPTS dye with HEPES buffer (10 mM HEPES, 100 mM NaCl, pH = 7.0). Collected vesicles were diluted to 6 mL to get POPC‒LUVs**⊃**HPTS.

**Final conditions**: ~ 5.0 mM POPC, Inside: 1 mM HPTS, 10 mM HEPES, 100 mM NaCl, pH = 7.0, Outside: 10 mM HEPES, 100 mM NaCl, pH = 7.0.

**Ion transport activity by HPTS assay:** In clean and well-dried fluorescence cuvette, 1975 µL of HEPES buffer (10 mM HEPES, 100 mM NaCl, pH =7.0) and 25 µL of POPC‒LUVs**⊃**HPTS vesicle were added. The cuvette was placed with slow stirring using a magnetic stirrer equipped Fluorometer (*t* = 0 s). The time-dependent HPTS emission intensity was monitored at λ_em_ = 510 nm (λ_ex_ = 450 nm). A pH gradient (𝛥 pH ~ 0.8) was formed between the intra- and extravesicular compartments by the addition of 20 µL NaOH (0.5 M) at *t* = 20 s. Varying concentrations of channel-forming molecule in DMSO were added at *t* = 100 s. Finally, the vesicles were lyzed by the addition of 10% Triton X-100 solution (25 µL) at *t* = 300 s to remove the pH gradient (Figure S11).

**Figure S30.** Schematic representation of ion transport activity across POPC‒LUVs**⊃**HPTS vesicle (A), and (B) normalization window for same fluorescence ion transport kinetics experiment.

The time-dependent data were normalized to fractional fluorescence intensity (in percentage) using Equation S2

*I_F_* = [(*I_t_* − *I_0_*) / (*I_∞_* − *I_0_*)] × 100 Equation S3

where, *I_0_* = Fluorescence intensity just before the channel forming molecule addition (at 0 s), *I_∞_* = Final fluorescence intensity after addition of Triton X‒100, *I_t_* = Fluorescence intensity at time *t*.

**Dose-response activity in POPC‒LUVs⊃HPTS**: The fluorescence kinetics of **3** at different concentrations was studied over time. The concentration profile data were evaluated at *t* = 180 s to get effective concentration, EC*_50_* (i.e. the concentration of transporter needed to achieve 50% ion efflux activity) using the Hill equation (Equation S4):

*Y* = *Y_∞_* + (*Y*_0_ – *Y_∞_*) / [1 + (c/*EC_50_*)^n^] Equation S4

where, *Y*_0_ = Fluorescence intensity just before the addition of channel forming molecule (at *t* = 0 s), *Y_∞_* = Fluorescence intensity with excess compound concentration, c = concentration of channel forming molecule, and *n* = Hill coefficient (i.e. indicative for the number of monomers needed to form an active channel structure).

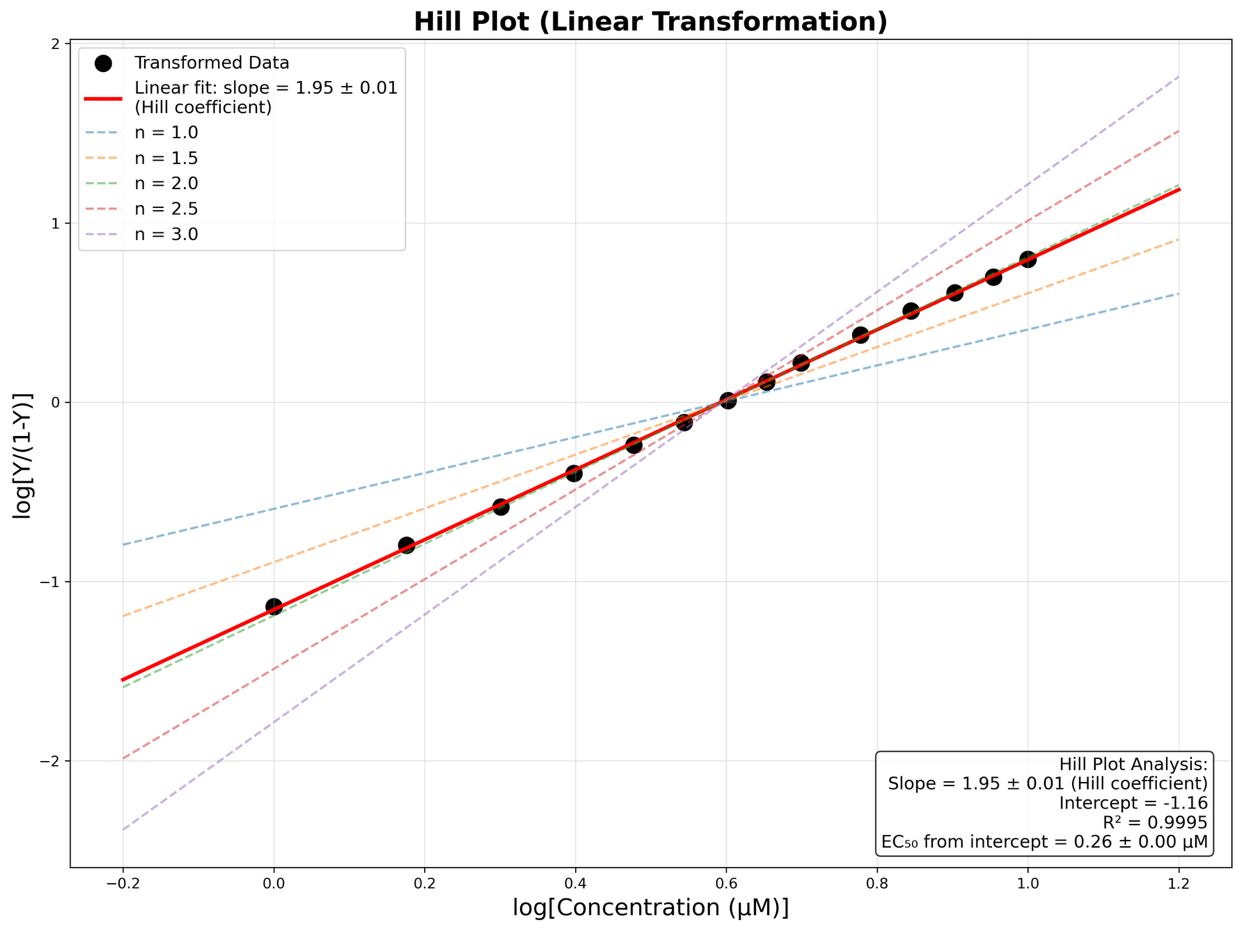


C

**Figure S31.** (A) Concentration-dependent ion transport activity of compound **3**-*Z* (0‒11 µM) with NaCl salt across POPC‒LUVs**⊃**HPTS. (B) Hill plot of compound **3**-*Z* at *t* = 180 s. (C) Log-Log Hill plot of compound **3**-*Z*, to visualise fit to Hill Coefficient = 2 over other options.

**Ion selectivity studies across POPC‒LUVs⊃HPTS**:

**Buffer and stock solution preparation:** HEPES buffer was prepared by dissolving an appropriate amount of solid HEPES and a salt (NaCl, NaBr, NaI, NaNO_3_, LiCl, KCl, RbCl, and CsCl) in MilliQ water to get 10 mM HEPES and 100 mM salt respectively. Subsequently, the pH was adjusted to 7.0 by the addition of 0.5 M NaOH solution. The stock solution of **3**-*Z* was prepared in HPLC grade DMSO solution for the studies.

**Anion selectivity assay by anion gradient assay:**^S7^ In a clean fluorescence cuvette, 1975 μL of HEPES buffer (10 mM HEPES, 100 mM NaX, at pH = 7.0; where, X^–^ = Cl^–^, Br^–^, I^–^, NO_3_^–^) was added, followed by addition of 25 μL of 100 mM NaCl entrapped POPC-LUVs⊃HPTS vesicles with slow stirring by magnetic stirrer in Cary Eclipse Fluorometer Agilent at *t* = 0 s. The time-dependent HPTS emission intensity was monitored at λ_em_ = 510 nm (λ_ex_ = 460/403 nm) after the addition of channel forming molecule **3**-*Z* at *t* = 100 s. Change in the HPTS fluorescence activity was monitored up to *t* = 300 s. For data analysis and comparison, time (X-axis) was synchronised between the point of addition of channel forming molecule (i.e. *t* = 100 s was synchronised to *t* = 0 s) and the endpoint of the experiment (i.e. *t* = 300 s was synchronised to *t* = 200 s). Fluorescence intensities were normalized to fractional emission intensity as F_t_ / F_0_. where F_t_ = relative fluorescence intensity at time t, and F_0_ = relative fluorescence intensity just before the addition of channel forming molecule (at *t* = 0 s). We attribute the unusual curve of the NaI experiments to the interplay of the two transport processes at play - H^+^/I^-^ influx and H^+^/Cl^-^ efflux – as observed in similar systems in reference S7.

**Figure S32**. (A) Schematic representation of the anion gradient assay across POPC‒LUVs**⊃**HPTS. (B) Anion selectivity of **3**-*Z* (1 μM) with intravesicular NaCl salt and extravesicular NaX salts across POPC‒LUVs**⊃**HPTS (without NaOH pulse).

**Cation selectivity assay**: In a clean fluorescence cuvette, 1975 μL of HEPES buffer (10 mM HEPES, 100 mM NaCl, at pH = 7.0; where, M^+^ = Li^+^, Na^+^, K^+^, and Rb^+^) was added, followed by addition of 25 μL of 100 mM NaCl entrapped POPC‒LUVs**⊃**HPTS vesicle with slow stirring by magnetic stirrer in a Fluorometer (at *t* = 0 s). The time-dependent HPTS emission intensity was monitored at λ_em_ = 510 nm (λ_ex_ = 450 nm). A pH gradient (𝛥 pH ~ 0.8) was formed between the intra- and extra-vesicular compartments by the addition of 20 µL NaOH (0.5 M) at *t* = 20 s. The compound **3**-*Z* was added at *t* = 100 s, and at *t* = 300 s, 25 μL of 10% Triton X‒100 was added to lyze all vesicles for the complete dissipation of the pH gradient. For data analysis and comparison, time (X-axis) was synchronised between the point of addition of channel forming molecule (i.e. *t* = 100 s was synchronised to *t* = 0 s) and the endpoint of the experiment (i.e. *t* = 300 s was synchronised to *t* = 200 s) using Eq. S1. Fluorescence intensities (*I_t_*) were normalized to fractional emission intensity *I_F_* using Equation S3.

**Mechanistic study of ion transport across POPC‒LUVs⊃HPTS using CCCP and valinomycin coupled assays:** ^S8^

**Preparation of buffer and stock solution for CCCP and valinomycin assay:** The buffer solution was prepared using MilliQ water, 100 mM of NaCl, and 10 mM of HEPES. The pH of the solution was adjusted 7.0 by the addition of a required amount of 0.5 M NaOH solution. Then 1 mM of HPTS solution was prepared in the same buffer solution. Similarly, the buffer solution for valinomycin assay was prepared using MilliQ water, 100 mM of KCl, and 10 mM of HEPES. The stock solutions of **3**-*Z*, FCCP, and valinomycin for the HPTS assay were prepared using HPLC grade DMSO.

**Preparation of POPC‒LUVs⊃HPTS vesicles for FCCP and Valinomycin assay:** The vesicles for the FCCP assay were prepared following the same procedure as previously.

**Ion transport activity in the presence of CCCP**: In a clean and dry fluorescence cuvette 1975 μL of HEPES buffer (10 mM HEPES, 100 mM NaCl, pH = 7.0) was taken followed by the addition of 25 μL of POPC−LUVs⊃HPTS with slow stirring by a magnetic stirrer in a Fluorometer (at *t* = 0 s). HPTS fluorescence emission intensity was monitored with time and *F_t_* was observed at λ_em_ = 510 nm (λ_ex_ = 450 nm). At *t* = 20 s, 20 μL of 0.5 M NaOH was added to the cuvette to establish a pH gradient between the intra- and extra-vesicular compartments. CCCP (0.5 μM) was added at *t* = 50 s and channel molecule **3**-*Z* was added at *t* = 100 s, and finally at *t* = 300 s, 25 μL of 10% Triton X-100 was added to that cuvette resulting dissipation of the pH gradient. Fluorescence intensities (*F_t_*) were normalized to fractional emission intensity *I_F_* using Equation S2 and the time scale was synchronised according to Equation S1.

**Ion transport activity in the presence of valinomycin**: In a clean and dry fluorescence cuvette, 1975 μL of HEPES buffer (10 mM HEPES, 100 mM NaCl, pH = 7.0) was added followed by the addition of 25 μL of POPC−LUVs⊃HPTS with slow stirring by a magnetic stirrer in a Fluorometer (at *t* = 0 s). The time course of HPTS fluorescence emission intensity, *F_t_*, was observed at λ_em_ = 510 nm (λ_ex_ = 450 nm). A pH gradient (𝛥 pH ~ 0.8) was formed between the intra- and extravesicular compartments by the addition of 20 µL NaOH (0.5 M) at *t* = 20 s. Valinomycin (2 pM) was added at *t* = 50 s and channel molecule **3**-*Z* was added at *t* = 100 s and finally at *t* = 300 s 25 μL of 10% Triton X-100 was added to lyze the vesicles dissipating the pH gradient. Fluorescence intensities (F_t_) were normalized to fractional emission intensity *I_F_* using Equation S1.

**Figure S33**. (A) Representations of fluorescence-based CCCP and (B) Valinomycin assays across POPC−LUVs⊃HPTS.

CCCP, a selective H^+^ carrier, was first investigated. Under the conditions of the HPTS assay, CCCP is expected to facilitate intravesicular H^+^ efflux to the extravesicular medium. When the compound **6** was coupled with CCCP, the transport rate showed no significant enhancement indicating that **6** does not show a cooperative effect with CCCP. This study suggested that a symport mechanism is operating during ion transport. Subsequently, Valinomycin, a K^+^ selective carrier, was coupled with **3**-*Z* in the HPTS assay to compare the preferential selectivity of compound **3**-*Z*. Under iso-osmolar KCl (extravesicular) vs NaCl (intravesicular) and application of NaOH gradient in the extravesicular buffer, compound **3**-*Z* did not show any significant difference in the transport rate in the absence and presence of valinomycin, again supporting a symport mechanism rather than antiport.

**Preparation of POPC-LUVs⊃Lucigenin with intravesicular Cl^‒^**: In a clean and dry small (10 mL) round bottom flask, 0.5 mL of 1-palmitoyl-2-oleoyl-sn-glycero-3-phosphocholine-cholesterol in the ratio 7:3 (POPC, 25 mg/mL stock in CHCl_3_) was added. The solution was dried by a flow of nitrogen with continuous rotation to form a thin transparent film of POPC. The transparent film was kept in a high vacuum at RT for 4 h to remove all traces of CHCl_3_. The resulting film was hydrated with 0.5 mL buffer solution (1 mM lucigenin, 10 mM phosphate buffer, and 225 mM NaCl, pH = 7.0), and the resulting suspension was vortexed at 10 min intervals for 1 h. This hydrated suspension was subjected to 21 cycles of freeze-thaw (liquid N_2_, 55 °C) followed by extrusion through 200 nm pore size containing polycarbonate membrane 29 times (this must be an odd number), to form a uniform distribution of vesicles with an average 200 nm diameter. Extravesicular dye was removed by gel filtration (using Sephadex G-50) with buffer solution (10 mM phosphate buffer and 225mM NaCl, pH = 7.0), and diluted to 2 mL to get POPC‒LUVs⊃lucigenin. Final conditions: ~ 5 mM POPC; Inside: 1 mM lucigenin, 10 mM phosphate buffer, 225 mM NaCl, pH = 7.0; Outside: 10 mM phosphate buffer, 225 mM NaCl, pH = 7.0.

**Figure S34**. (A) Schematic representation of chloride efflux assay using POPC-Cholesterol LUVs⊃Lucigenin and (B) normalization window for same fluorescence ion transport kinetics experiment.

**Effect of extravesicular NO_3_^‒^ and SO_4_^2‒^ on the chloride efflux from POPC-LUVs⊃Lucigenin:** In a clean and dry fluorescence cuvette, 50 μL of above lipid solution and 1950 μL of an isoosmolar solution of different NaX (X^‒^ = NO_3_^‒^ and SO_4_^2‒^) salts were taken and slowly stirred in a Fluorometer (at t = 0 s). The time course of lucigenin fluorescence emission intensity, F_t_ was monitored at λ_em_ = 535 nm (λ_ex_ = 450 nm). Compound **3** was added at t = 100 s and finally at t = 300 s, 25 μL of 10% Triton X100 was added to lyse all vesicles for 100% chloride efflux. For data analysis and comparison, time (X-axis) was normalized according to Equation S1 (i.e. the point of channel addition was normalized to t = 0 s). Relative fluorescence intensities (F/F_0_) were calculated using Equation S2.

**Figure S35**. (A) Cl^‒^ efflux across POPC-Cholesterol LUVs ⊃ Lucigenin by **3** (3 µm) in the presence of intravesicular Cl^‒^ and either SO_4_^2‒^ or NO_3_^‒^ as isoosmolar extravesicular anion.

1. **Ion Transport using DPPC‒LUVS**⊃**HPTS:**^S10^

Compound **3** (1 µM) was added to a solution of 1,2-dipalmitoyl-sn-glycero-3-phosphocholine (DPPC, 350 µL, 28.3 mM in deacidified CHCl_3_). The receptor/lipid mixture was evaporated under a gentle stream of N_2_ and dried under high vacuum for 16 h at RT. The resulting film was hydrated with 500 µL of an aqueous solution of HPTS dye (1 mM) in NaCl (100 mM) and was then sonicated for 30 seconds followed by stirring for 1 h at 50 °C to give heterogenous LUVs. These heterogenous LUVs were disrupted by 12 freeze-thaw cycles and the solution was carefully extruded (29 times) through a polycarbonate membrane (200 nm pore size) at 50°C to give a uniform distribution of LUVs with an average 200 nm diameter. The external HPTS was removed by passing the solution through a size exclusion column (Sephadex 50-G, eluted in 100 mM NaCl) and the collected vesicles were made up to a total volume of 2 mL (0.4 mM in lipid) with NaCl solution (100 mM).

A cuvette containing 2 mL of the vesicle solution (in buffer) was placed in a fluorescence spectrometer and held at the temperature of interest (either 25 ^o^C or 45 ^o^C). The liposomal solution was irradiated with either 415 nm or 530 nm LEDs for 3 min, after which recording was started, and addition of NaOH (50 µL, 0.5 M) at *t* = 50 s. Fluorescence was monitored over time. Finally, Triton X-100 was added to lyze the vesicles dissipating the pH gradient to get a 100% fluorescence readout. The fluorescence intensities (*F_t_*) were normalized to fractional emission intensity *I_F_* using Equation S3:

**Figure S36**. (A) Schematic representation of DPPC‒LUVS⊃HPTS. (B) Normalization window for same fluorescence kinetics experiment of ion transport.


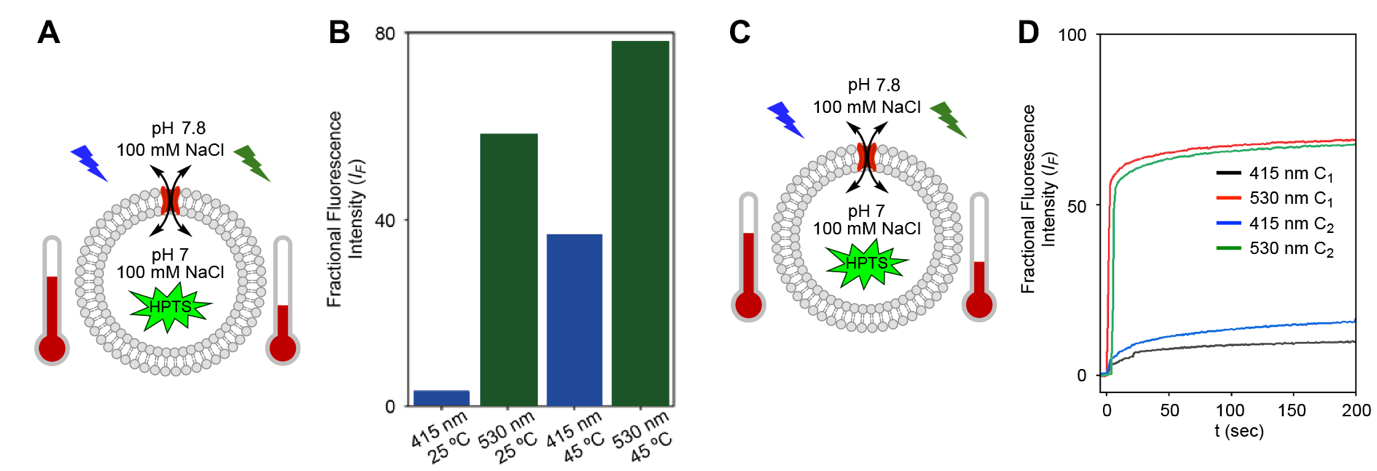


**Figure S37.** (A) Schematic representation and (B) ion transport data facilitated by **3** across DPPC-LUVs⊃HPTS at 45 °C and 25 °C, after 415 nm (*E*) and 530 nm (*Z*) irradiation in liposomes for 20s. (C). Schematic representation and Ion transport facilitated by **3** across DPPC-LUVs⊃HPTS at 25 °C (*C*_1_ and *C*_2_ represent cycle 1 and cycle 2 respectively of *in situ* photoswitching prior to addition of base pulse).

1. **Effect of Fatty Acids on Rate of Transport:**^S11^

**Preparation of POPC‒LUVs**⊃**HPTS with NaCl:** A thin lipid film was prepared by evaporating a solution of chloroform solution of POPC in a round-bottom flask and the lipid film formed was dried under vacuum for 12 h. Then, the lipid film was hydrated by vortexing with an internal solution containing HPTS (1 mM) and potassium gluconate (100 mM) buffered at pH 7.0 with 10 mM HEPES. The lipid suspension was subjected to nine freeze/thaw cycles and then extruded 19 times through a 200 nm polycarbonate membrane. The unentrapped HPTS was removed by size exclusion chromatography on a Sephadex G-50 column using an external solution of potassium gluconate (100 mM) buffered at pH 7.0 with 10 mM HEPES, to obtain a vesicle stock suspension (with lipid concentration ~ 10 mM).

When fatty acid removal by Bovine Serum Albumin (BSA) was required, fatty acid-free BSA was dissolved in the vesicle stock suspension to a final BSA concentration of 1 mol% (with respect to lipid). The BSA-containing vesicle stock suspension was stirred for 20 min before being used for membrane transport studies. For each test, the vesicle stock suspension was diluted using the external solution to obtain a 2 mL sample containing 0.1 mM of lipid. The sample was stirred at 298 K and the fluorescence ratio of HPTS (λ_ex_ = 450 nm, λ_em_ = 510 nm) was recorded over time.

**Figure S38**: (A) Schematic representation of the electrogenic H^+^/OH^−^ transport assay based on following the rate of pH gradient dissipation across POPC-LUVs ⊃HPTS with a mean diameter of ∼200 nm. (B) H^+^/OH^−^ transport induced by **3** (2 µM) in presence and absence of Oleic Acid (OA) as Fatty Acid (FA), and/or Bovine Serum Albumin (BSA) measured by the assay shown in (A) under various conditions. Valinomycin (2 pM) was used in all cases. The vesicles were treated with monensin (1uM) at 200 s to collapse the pH gradient for calibration of HPTS fluorescence.

1. **Vesicle leakage studies by Carboxyfluorescein assay:**

**Preparation of EYPC-LUVs⊃CF**:^S12^ A thin lipid film was prepared by evaporating a solution of 12.5 mg POPC in 0.5 ml CHCl_3_ in vacuo for 4 h. After that lipid film was hydrated with 0.5 mL buffer (10 mM HEPES, 10 mM NaCl, 50 mM CF, pH 7.0) for 1 h with occasional vortexing of 4-5 times and then subjected to freeze-thaw cycle (≥ 20 times). The vesicle solution was extruded through a polycarbonate membrane with 200 nm pores 19 times (has to be an odd number). The extracellular dye was removed size exclusion chromatography (Sephadex G-50) with 10 mM HEPES buffer (100 mM NaCl, pH 7.0) Final concentration: ~2.5 mM EYPC lipid; intravesicular solution: 10 mM HEPES, 10 mM NaCl, 50 mM CF, pH 7.0; extravesicular solution: 10 mM HEPES, 100 mM NaCl, pH 7.0.

**CF leakage assay**: In a clean and dry fluorescence cuvette 25 µL of above lipid solution and 1975 µL of 10 mM HEPES buffer (100 mM NaCl, pH 7.0) was taken and kept in slowly stirring condition by a magnetic stirrer equipped with the fluorescence instrument (at t = 0 s). The time course of CF fluorescence emission intensity, Ft was observed at λ_em_ = 517 nm (λ_ex_ = 492 nm). Compound **3-*E* or 3-*Z*** was added at t = 100 s and finally at t = 300 s, 25 μL of 10% Triton X-100 was added to lyze those vesicles. Fluorescence intensities were normalized to fractional emission intensity *I_F_* according to Equation S3. This study confirmed that neither the bilayer membranes are defected nor large transmembrane pores are formed by **3**.

**Figure S39:** (A) Schematic illustration of CF leakage assay using POPC-LUVs⊃HPTS and CF. (B) Normalization window for same fluorescence kinetics experiment of ion transport. (C)Carboxyfluorescein efflux assay for compound **3-*E*** and **3-*Z*** (2 µM).

1. **Chloride transport activity across POPC‒LUVs**⊃**lucigenin vesicles under different conditions**:

**Buffer and stock solution preparation**: Same procedure as above with only pH adjustment as needed.

**Preparation of POPC‒LUVs**⊃**lucigenin**: Same procedure as above with only pH adjustment as needed.

**Ion transport activity in POPC‒LUVs⊃lucigenin at different pH values:** Same procedure as above with only using appropriate buffers for respective liposomes with a specific pH.

**Figure S40.** Chloride anion transport of **3** (2.5 μM) under 415 nm and 530 nm irradiation at (A) pH 5.5, (B) pH 7, and (C) pH 8.5.

**Ion transport activity in POPC‒LUVs⊃lucigenin at pH 5.5 with 1-aminoadamantane hydrochloride:** In clean and dry fluorescence cuvette, 1950 µL of buffer solution (10 mM phosphate buffer, 225 mM NaNO_3_, pH = 7.0), 50 µL POPC‒LUVs**⊃**lucigenin was added. This suspension was slowly stirred by magnetic stirrer in the Fluorometer (at *t* = 0 s). The fluorescence intensity of lucigenin was monitored at λ_em_ = 535 nm (λ_ex_ = 450 nm) over time. Then, the solution of 1-aminoadamantane hydrochloride in DMF was added to make its effective concentration 100 µM at *t* = 0 s, followed by formation of a chloride gradient by the addition of 2.0 M NaCl (33.3 µL) at *t* = 20 s between the intra- and extravesicular compartments. Finally, channel-forming molecule **3** was added (after isomerization in solution to enrich the respective isomer) at *t* = 100 s, and fluorescence intensity was monitored over time. The vesicles were lyzed by adding 10% Triton X‒100 (25 μL) at *t* = 300 s to dissipate the chloride gradient. The collected data was then normalized to the percentage change in fluorescence intensity over time using the equation S2.

**
**

**Figure S41.** (A) Structure of 1-aminoadamantane hydrochloride, and (B) Chloride anion transport of **3**-*Z* (2.5 μM) under 415 nm and 530 nm irradiation at pH 5.5 in absence and (C) presence of 1-aminoadamantane hydrochloride. (D) Chloride anion transport of **3**-*E* (2.5 μM) under 415 nm irradiation at pH 7 and (E) pH 8.5 in presence of 1-aminoadamantane hydrochloride.

**Measuring the initial rates of transport of 3 under different conditions:** ^S4^

Following the procedure described above, fluorescence data from experiments under different conditions were processed to calculate the initial rate (*I* s^-1^) of transport. To calculate the half-life time values (*t*_1/2_), the fluorescence kinetics data (0 – 200 s) from each experiment was plotted as a function of time and fitted with the following single exponential decay equation (ExpDec 1) in the origin 2024:

$$\frac{Fo}{F}=y -ae^{-bt}$$

The half-life time values *t*_1/2_ were calculated from the fitting parameter “𝑏”, using the following formula:

*t*_1/2_ = 0.693/b

To calculate the initial rates (*I)*, the fluorescence data (0 – 200 s) from each experiment was plotted against time, and fitted with the following double exponential decay equation (ExpDec 2) in the origin 2024:

$\frac{Fo}{F}=y-ae^{-bt}-ce^{-dt}$

The initial rates (*I*) were calculated using the fitting parameters a, b, c, and d, using the following equation:

*I* = (a x b) + (c x d)

The calculated values under different conditions are presented in table S1 as below.

**
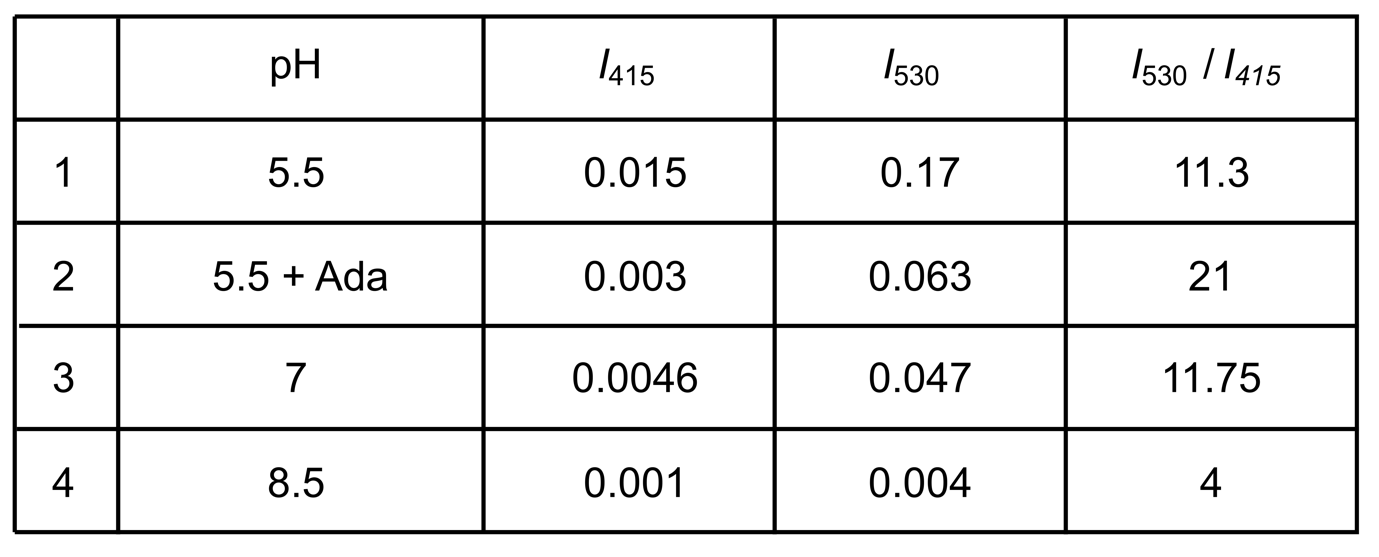
**

**Table S1**. Initial rate of transport by **3** under 415 nm and 530 nm irradiation at different pH values and in the presence or absence of 1-aminoadamantane hydrochloride at pH 5.5, showing the range of accessible rates using different stimuli.

**Figure S42.** Changes in relative fluorescence F_0_/F and single exponential decay fit for the transport of Cl^−^ ions into LUVs by **3**-*E* at pH 7 (Dec 2).

**Figure S43.** Changes in relative fluorescence F_0_/F and single exponential decay fit for the transport of Cl^−^ ions into LUVs by **3**-*E* at pH 7 (Dec 1).

**Figure S44.** Changes in relative fluorescence F_0_/F and single exponential decay fit for the transport of Cl^−^ ions into LUVs by **3**-*Z* at pH 7 (Dec 2).

**Figure S45.** Changes in relative fluorescence F_0_/F and single exponential decay fit for the transport of Cl^−^ ions into LUVs by **3**-*Z* at pH 7 (Dec 1).

**Figure S46.** Changes in relative fluorescence F_0_/F and single exponential decay fit for the transport of Cl^−^ ions into LUVs by **3**-*E* at pH 8.5 (Dec 2).

**Figure S47.** Changes in relative fluorescence F_0_/F and single exponential decay fit for the transport of Cl^−^ ions into LUVs by **3**-*E* at pH 8.5 (Dec 1).

**Figure S48.** Changes in relative fluorescence F_0_/F and single exponential decay fit for the transport of Cl^−^ ions into LUVs by **3**-*Z* at pH 8.5 (Dec 2).

**Figure S49.** Changes in relative fluorescence F_0_/F and single exponential decay fit for the transport of Cl^−^ ions into LUVs by **3**-*Z* at pH 8.5 (Dec 1).

**Figure S50.** Changes in relative fluorescence F_0_/F and single exponential decay fit for the transport of Cl^−^ ions into LUVs by **3**-*E* at pH 5.5 (Dec 2).

**Figure S51.** Changes in relative fluorescence F_0_/F and single exponential decay fit for the transport of Cl^−^ ions into LUVs by **3**-*E* at pH 5.5 (Dec 1).

**Figure S52.** Changes in relative fluorescence F_0_/F and single exponential decay fit for the transport of Cl^−^ ions into LUVs by **3**-*Z* at pH 5.5 (Dec 2).

**Figure S53.** Changes in relative fluorescence F_0_/F and single exponential decay fit for the transport of Cl^−^ ions into LUVs by **3**-*Z* at pH 5.5 (Dec 1).

**Figure S54.** Changes in relative fluorescence F_0_/F and single exponential decay fit for the transport of Cl^−^ ions into LUVs by **3**-*E* at pH 5.5 with 1-aminoadamantane hydrochloride (Dec 2).

**Figure S55.** Changes in relative fluorescence F_0_/F and single exponential decay fit for the transport of Cl^−^ ions into LUVs by **3**-*E* at pH 5.5 with 1-aminoadamantane hydrochloride (Dec 1).

**Figure S56.** Changes in relative fluorescence F_0_/F and single exponential decay fit for the transport of Cl^−^ ions into LUVs by **3**-*Z* at pH 5.5 with 1-aminoadamantane hydrochloride (Dec 2).

**Figure S57.** Changes in relative fluorescence F_0_/F and single exponential decay fit for the transport of Cl^−^ ions into LUVs by **3**-*Z* at pH 5.5 with 1-aminoadamantane hydrochloride (Dec 1).

**Dose-response activity in POPC‒LUVs⊃Lucigenin at pH 5.5:** In order to understand channel formation at acidic pH (5.5), the fluorescence kinetics of **3-*Z*** at different concentrations was studied over time using lucigenin vesicles at pH 5.5. The concentration profile data were evaluated at *t* = 180 s to get Hill coefficient, *n*, were evaluated using the Hill equation. The results suggest that dimer is the active channel structure at pH 5.5.

**Figure S58.** (A) Concentration-dependent ion transport activity of compound **3**-*Z* (0-1.2 µM) with NaNO_3_ salt across POPC‒LUVs**⊃**Lucigenin at pH 5.5. (B) Hill plot of compound **3**-*Z* at *t* = 180 s.

**Cation Selectivity of 3-*Z* in POPC‒LUVs⊃Lucigenin at pH 5.5:** We also tried to explore the cation selectivity (in attempt to understand impact of carboxylate protonation) of **3-*Z*** by varying different metal slats of chloride (NaCl, KCl, RbCl and LiCl) in the extravesicular buffer. If there is a preference for metal ions, we will see difference in the rate of transport for different salts. However, we did not see any significant difference for different metal salts, which signifies that the cation selectyivity is perhaps lost at acidic pH.

**Figure S59.** Cation Selectivity of **3***-Z* (0.8 µM) with NaNO_3_ salt across POPC‒LUVs**⊃**Lucigenin at pH 5.5.

To understand the channel formation at basic pH (8.5), the fluorescence kinetics of **3**-*Z* at different concentrations was studied over time using lucigenin vesicles at pH 8.5. However, the compound started precipitating after 4 µM concentration, and so entire concentration profile could not be completed.

**Figure S60.** Concentration-dependent ion transport activity of compound **3**-*Z* (0 - 4 µM) with NaNO_3_ salt across POPC‒LUVs**⊃**Lucigenin at pH 8.5.

# NMR Titrations - Host-Guest Association.

#


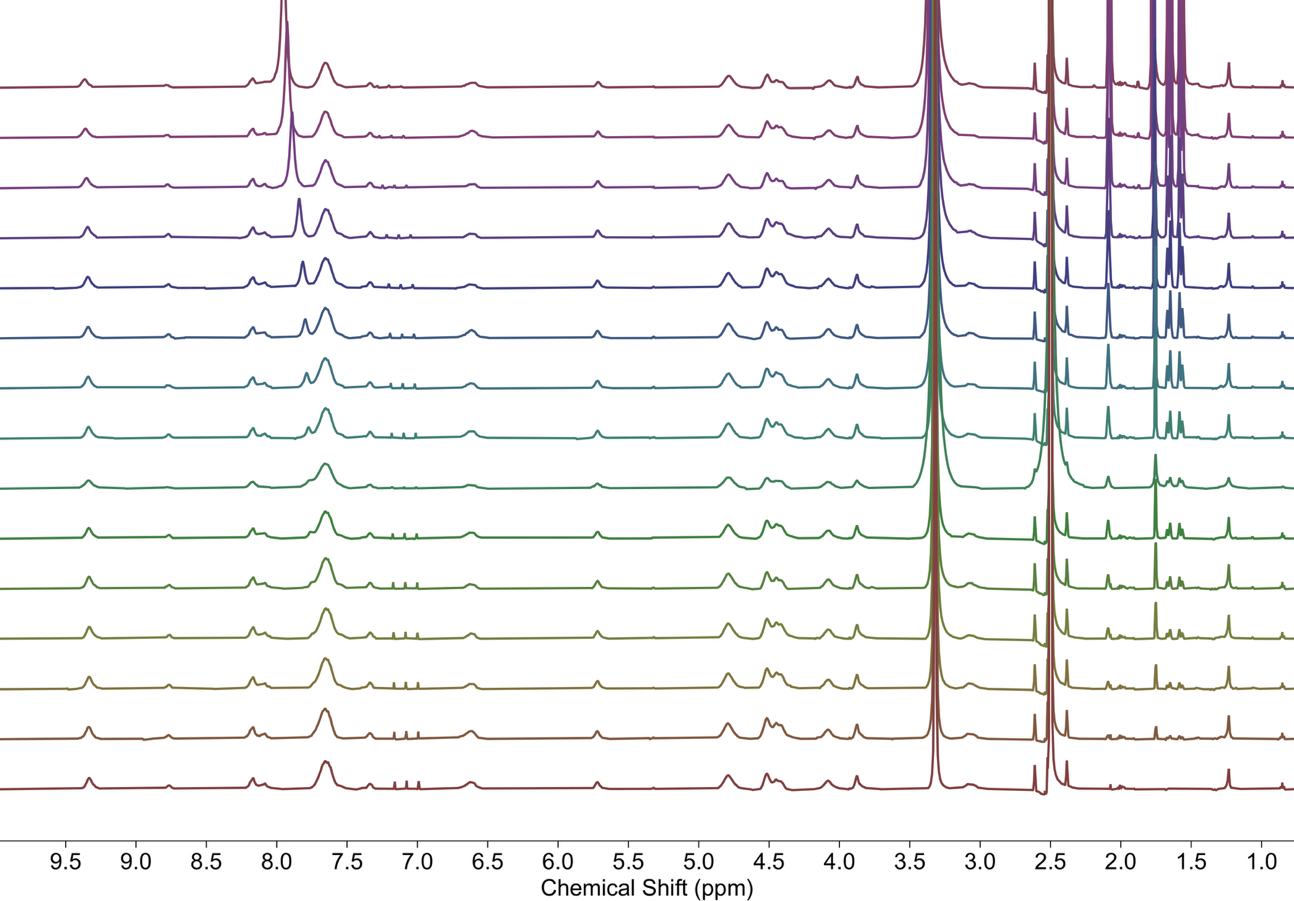
A stock solution of **3**-*E* and **3**-*Z* was prepared in DMSO-*d*_6_, and the concentration stated under the figure. 70 mM stock solution of guest was prepared in separate vials in DMSO-*d*_6_. In each case, 400 μL of host solution (**3**-*E* or **3**-*Z*) in an NMR tube was titrated with aliquots of 0.2 – 14 μL of guest solution to a typical total of 20 eq. of guest. To determine association constants, curve fitting of the experimentally obtained titration isotherms (equivalents of guest vs chemical shift of guest proton) was carried using a nonlinear least squares curve fitting procedure using the online software <https://supramolecular.org/>, with 1:1 global fitting models (Nelder-Mead method) found to provide the best fit.

**Figure S61.** ^1^H-NMR (DMSO-*d*_6_, 600 MHz) of host **3**-*E* (0.8 µM) at 298 K and varying equivalents of 1-aminoadamantane hydrochloride.


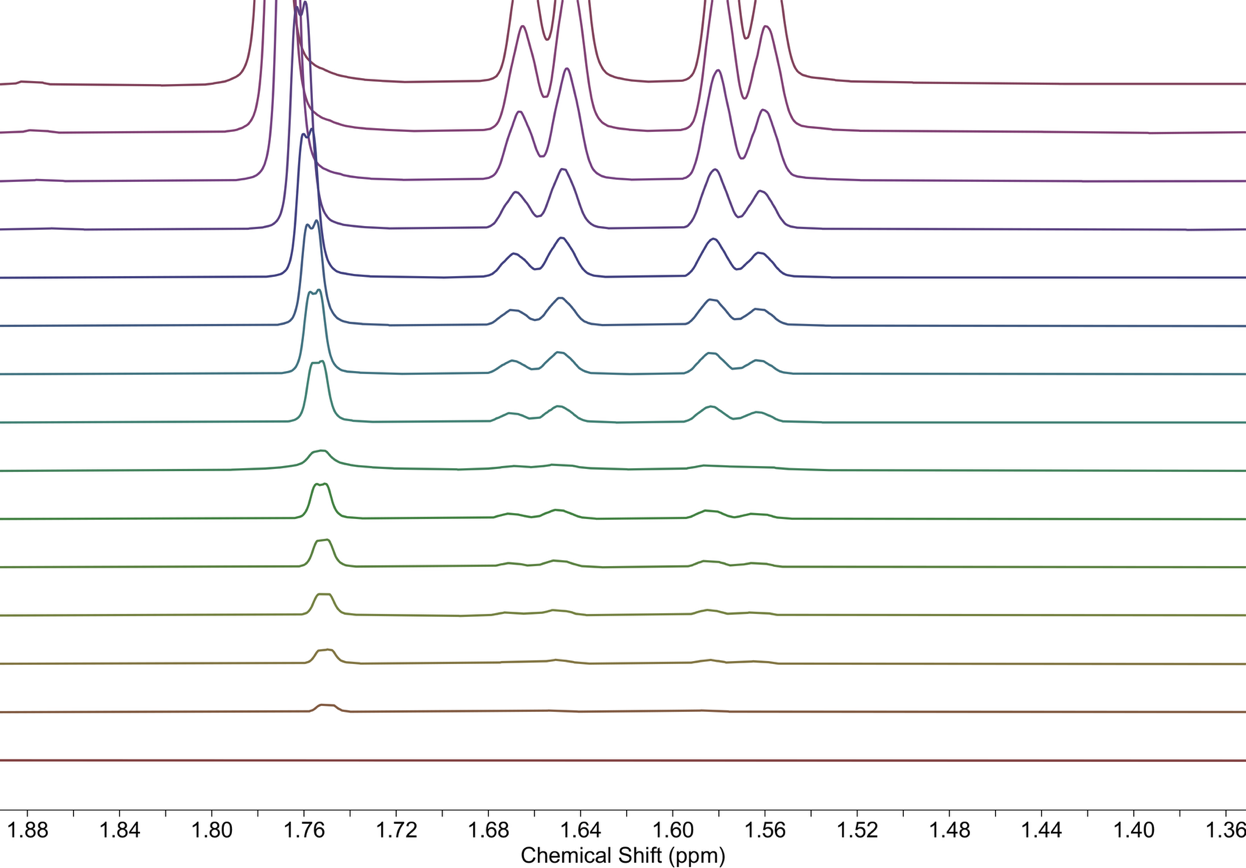


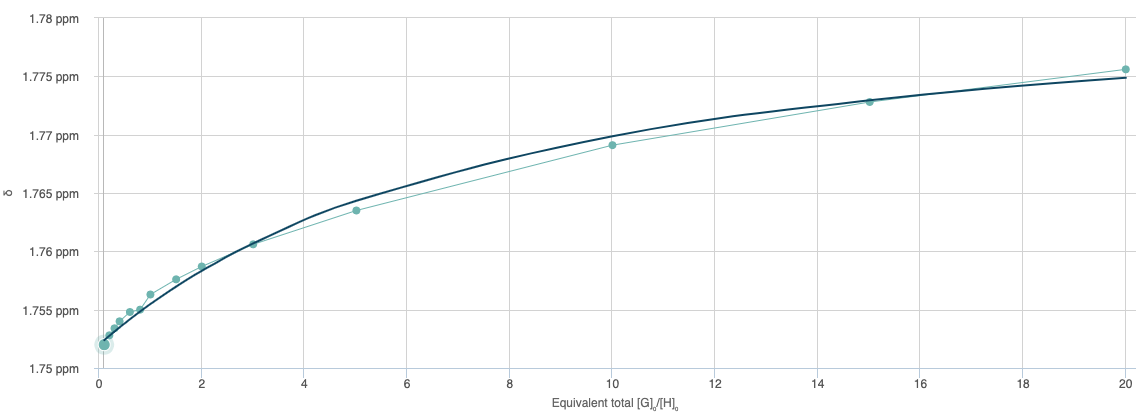
**Figure S62.** Zoomed ^1^H-NMR (DMSO-*d*_6_, 600 MHz) of host **3**-*E* (0.8 mM) at 298 K and varying equivalents of 1-aminoadamantane hydrochloride to highlight shifts at 1.76 ppm peak (corresponding to 1-aminoadamantane hydrochloride).


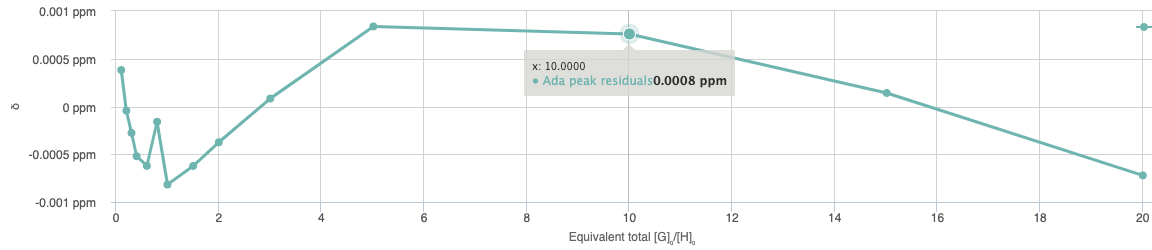


**Figure S63**. Binding data for **3**-*E* and 1-aminoadamantane hydrochloride. K_a_ = 349

M^-1^ ± 7.7%. The Bindfit URL for this experiment is as below:

<http://app.supramolecular.org/bindfit/view/776eeb9d-827c-419e-a688-7cc8147ec6aa>


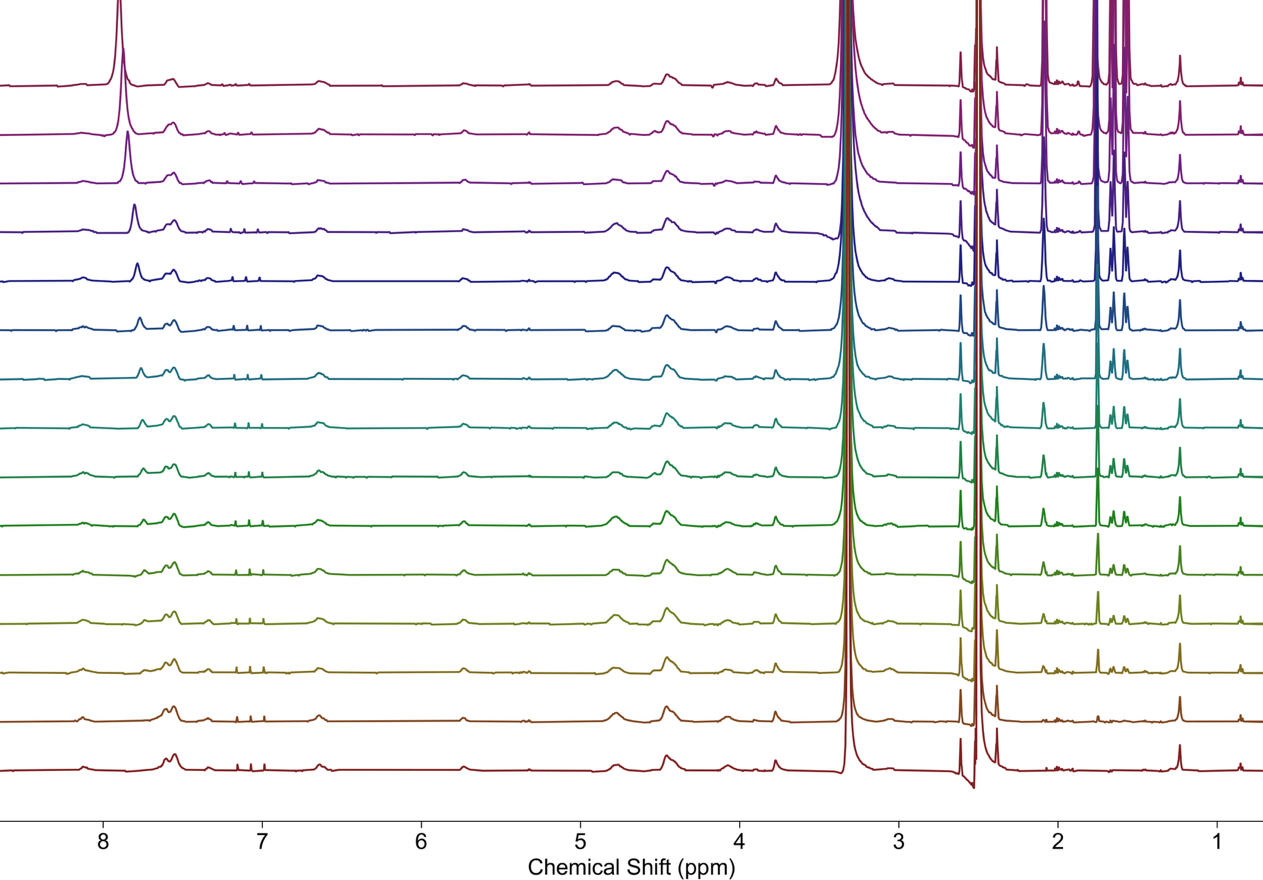


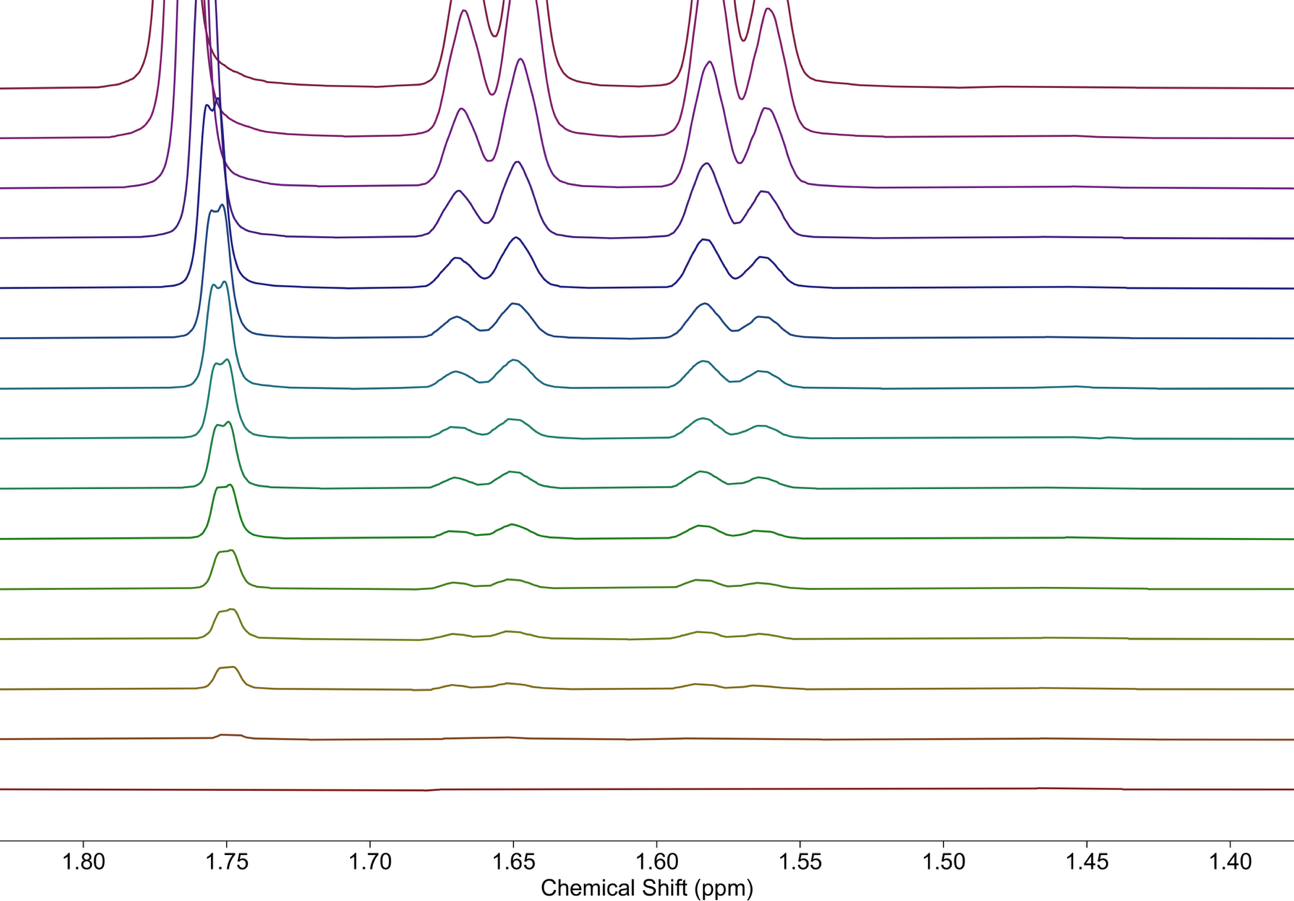
**Figure S64.** ^1^H-NMR (DMSO-*d*_6_, 600 MHz) of host **3**-*Z* (1 mM) at 298 K and varying equivalents of 1-aminoadamantane hydrochloride.

**Figure S65.** Zoomed ^1^H-NMR (DMSO-*d*_6_, 600 MHz) of host **3**-*Z* (1 mM) at 298 K and varying equivalents of 1-aminoadamantane hydrochloride to highlight shifts at 1.76 ppm peak (corresponding to 1-aminoadamantane hydrochloride).


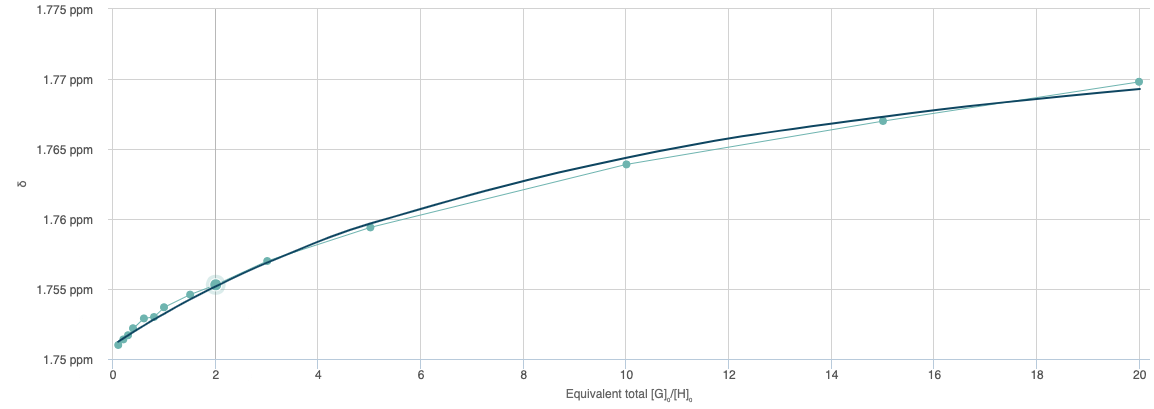


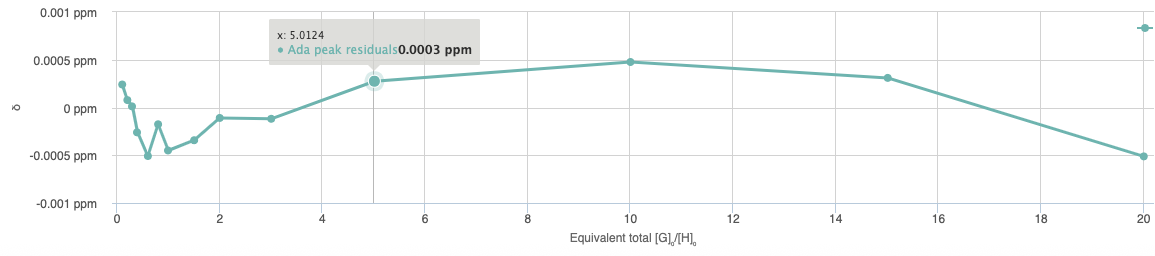


**Figure S66**. Binding data for **3**-*Z* and 1-aminoadamantane hydrochloride. K_a_ = 232

M^‒1^ ± 5.3%. The Bindfit URL for this experiment is as below:

<http://app.supramolecular.org/bindfit/view/2af6e0c4-2011-4abc-99e7-088a5ff78bb1>

# Planar bilayer conductance studies:^S13^

Following previously published methods [Maybe Leptihn Nat Prot 2013?]. A solution of 1,2-diphytanoyl-*sn*-glycero-3-phosphocholine lipid (DPhPC, Avanti Polar Lipids) in chloroform (50 mg/mL, 27 uL) was evaporated under nitrogen gas to create a thin white film. The film was then reconstituted in a 4:1 hexadecane:silicon oil (AR20) mixture to achieve a final concentration of 9 mg/mL. The assembly of a poly(methyl methacrylate) microfluidic device involved the preparation of a 0.75% (wt/vol) ultralow temperature gelling substrate agarose (Sigma-Aldrich) solution in Milli-Q water. After heating to 90°C, 140 uL was spun-coatedto a coverslip on the underside of the device and fixed in position. A 1.3% (wt/vol) rehydration agarose solution, containing 1.5 M KCl buffered with 10 mM HEPES (pH 7) was introduced through the device inlet to allow contact and hydration to the substrate agarose layer. The device featuring 16 wells, 1 mm in diameter, was filled with the lipid-in-oil mixture and incubated for 25 minutes to facilitate monolayer formation on the substrate. Meanwhile, aqueous droplets (∼ 200 nL) containing **3** (1 nM), were illuminated using LED equipment at 405 nm or 532 nm with power densities 11.32 mW/cm^2^ and 9.55 mW/cm^2^, respectively, while incubating in the same lipid-in-oil solution. Following incubation, droplets were dispensed into the wells, where they settled onto the substrate to afford bilayer formation upon contact. Ag/AgCl electrodes were positioned in the hydration agarose, while electrical contact with the droplet surface was established using a micromanipulator. Voltages were applied and bilayer currents were measured using an Axopatch 200B patch-clamp amplifier and headstage (Axon instruments, Molecular Devices, CA, USA). data were recorded using WinEDR V3.8.5 (John Dempster, Strathclyde University, UK) and signal acquisition was filtered with a Bessel filter at 1 kHz with an additional digital filter at 100 Hz implemented through QuB Software. ^S12-14^ The mean current values and their associated standard deviations were obtained by further analysing the data using python scripts.


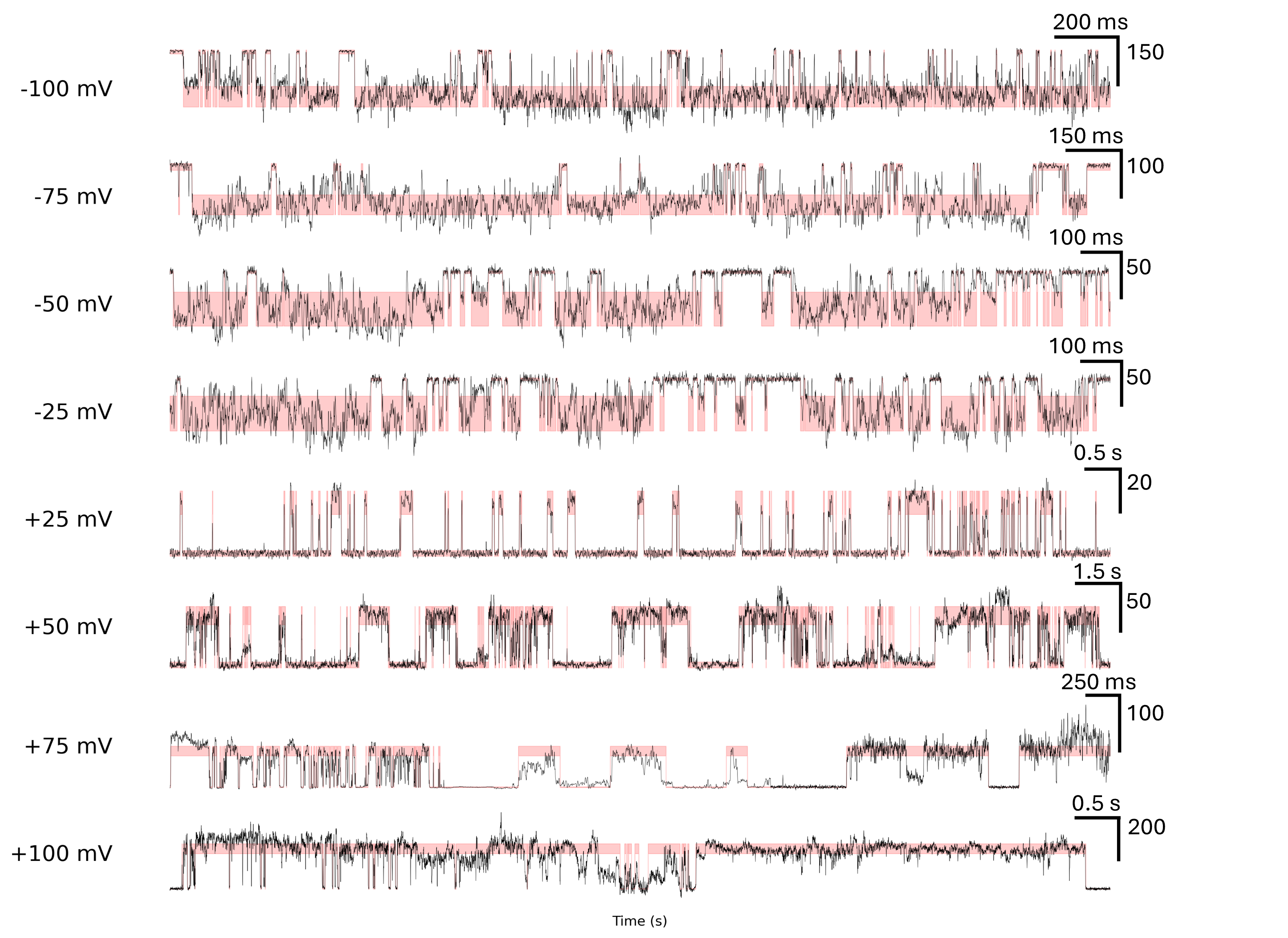


**Figure S67.** Full current traces recorded from DPhPC bilayers containing compound **3** pre-exposed to 532 nm light at various applied potentials. Electrical traces were filtered post-acquisition at 1 kHz low-pass Guassian filter using QuB. Red lines indicate idealisation using the segmental k-means method implemented through QuB which was later used for kinetic analysis of the channel. Representative scale bars are shown.


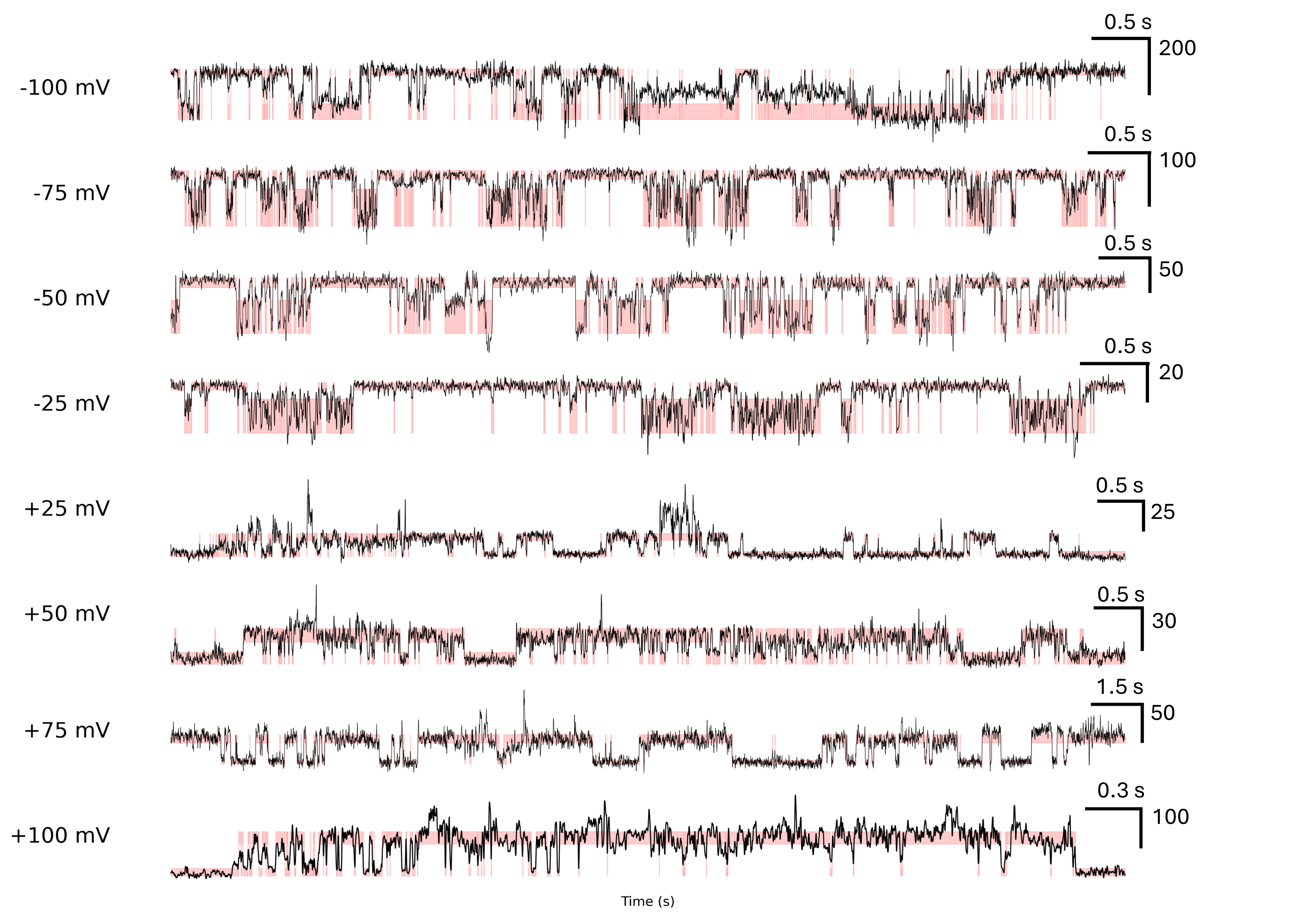


**Figure S68.** Full current traces recorded from DPhPC bilayers containing compound **3** pre-exposed to 405 nm light at various applied potentials. Analysis was performed as mentioned in Figure S67.


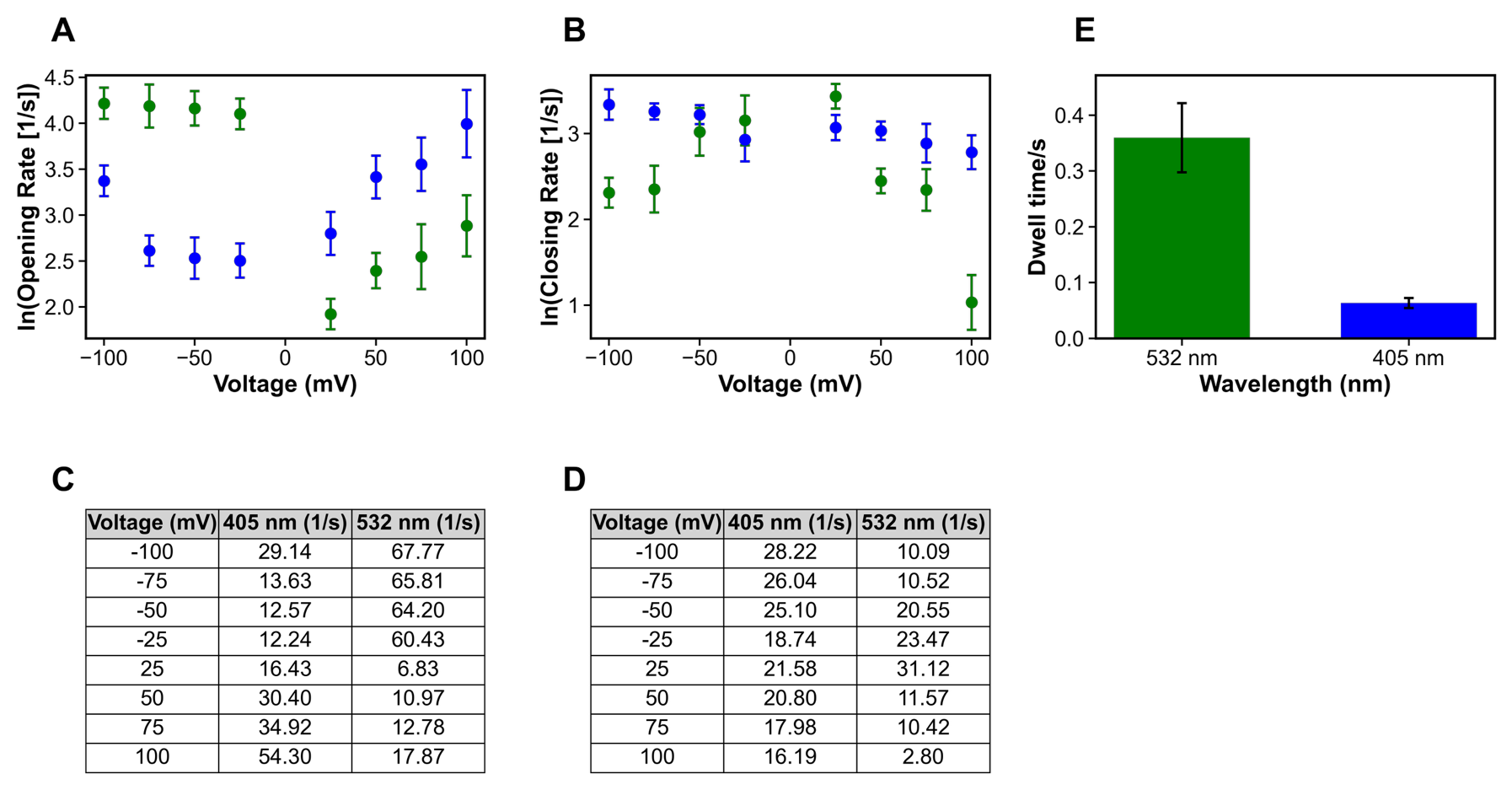


**Figure S69.** (A) and (B) display the kinetic transitions between the conformational states on a logarithmic scale for both opening rates (state 0 to state 1) and closing rates (state 1 to state 0), respectively, with error bars calculated using Standard Error Mean. (C) and (D) offer tabulated values corresponding to the data points in (A) and (B).

# Computational Calculations

Co-ordinates for the molecular models of **3**-*Z* and **3**-*E* were prepared by importing bond connectivities, drawn in *ChemDraw (22.2.0)*, into *ChemDraw3D* *(22.2.0)*. Initial co-ordinates were processed using MM2 forcefield parameters,^S17^ with default settings within ChemDraw3D. Models were imported into *Avogadro2*, and protonation state at pH 7.4 calculated using the *obabel* plugin.^S18^ Subsequent molecular calculations were performed with semi-empirical tight binding GFN2-xTB forcefield within the *xtb* programme developed by Grimme *et al*..^S19^ Geometry optimisation was undertaken to the crude level of energy convergence (E_conv_ = 1.312 kJ mol^-1^) at an electronic temperature of 300 K before being further optimised to ‘normal’ convergence (E_conv_ = 0.003 kJ mol^-1^) or better. The integrated analytical linearised Poisson-Boltzmann (ALPB) model for implicit solvation was invoked in all optimisations using default *xtb* parameters for octanol to simulate a membrane-like environment. ^S20,S21^

**B**

**A**


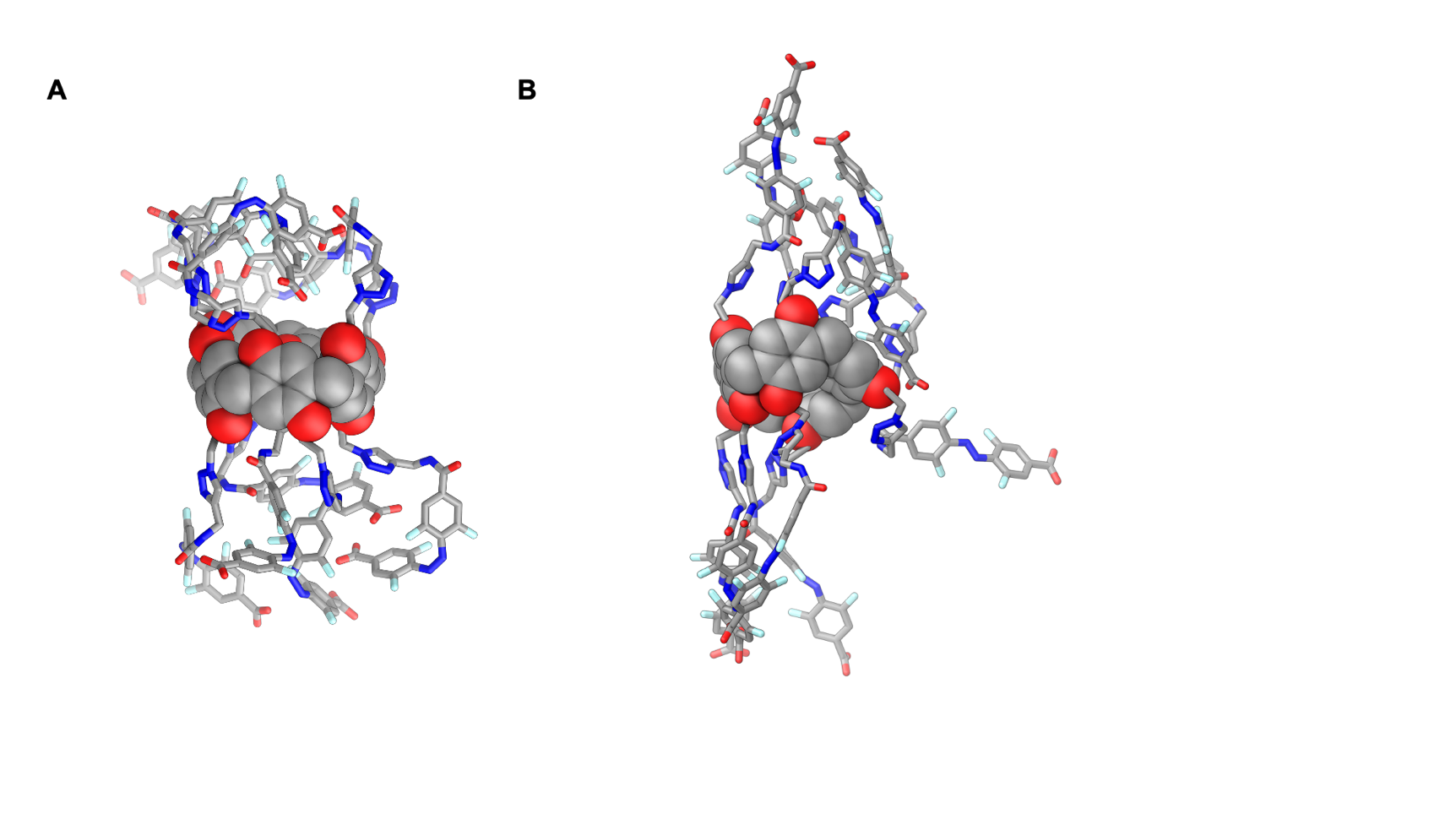


**Figure S70**. Side-view of GFN2-xTB optimised geometries of (A) **3**-*Z*-isomer and (B) **3**-*E* isomer to tight energy convergence and octanol implicit solvation parameters at an electronic temperature of 300 K. Pillar[5]arene core is highlighted with space-filled spheres.


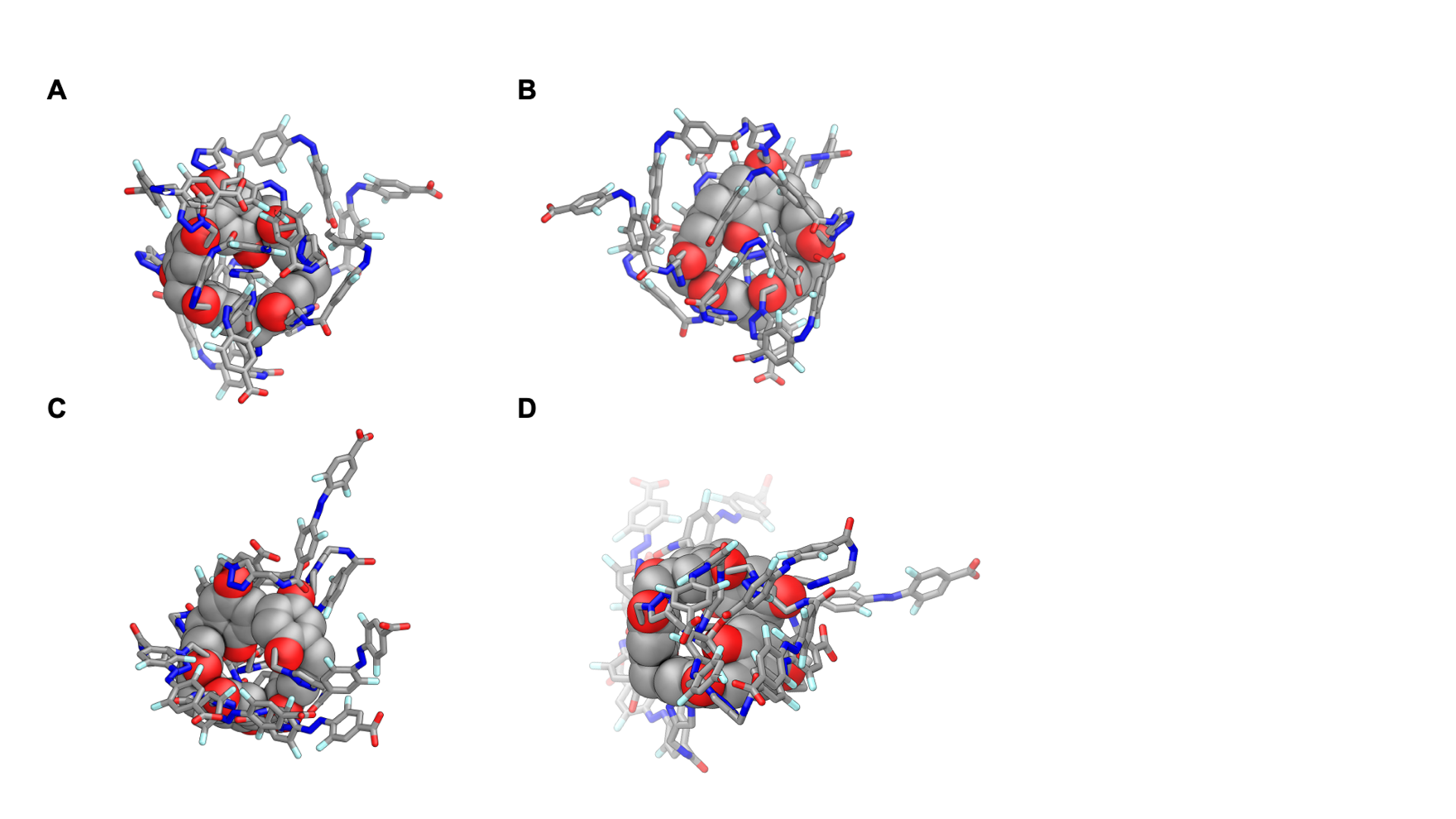

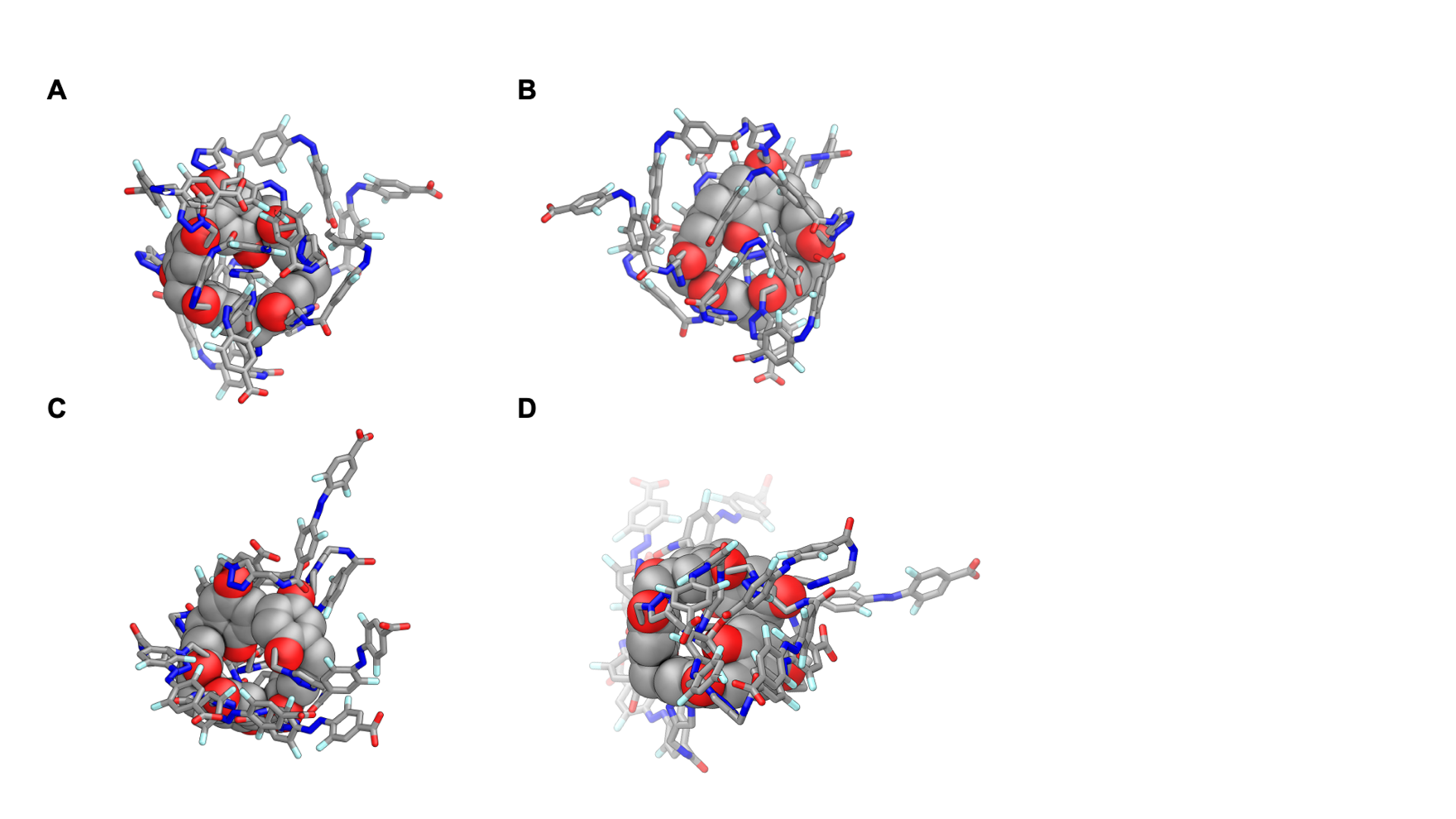

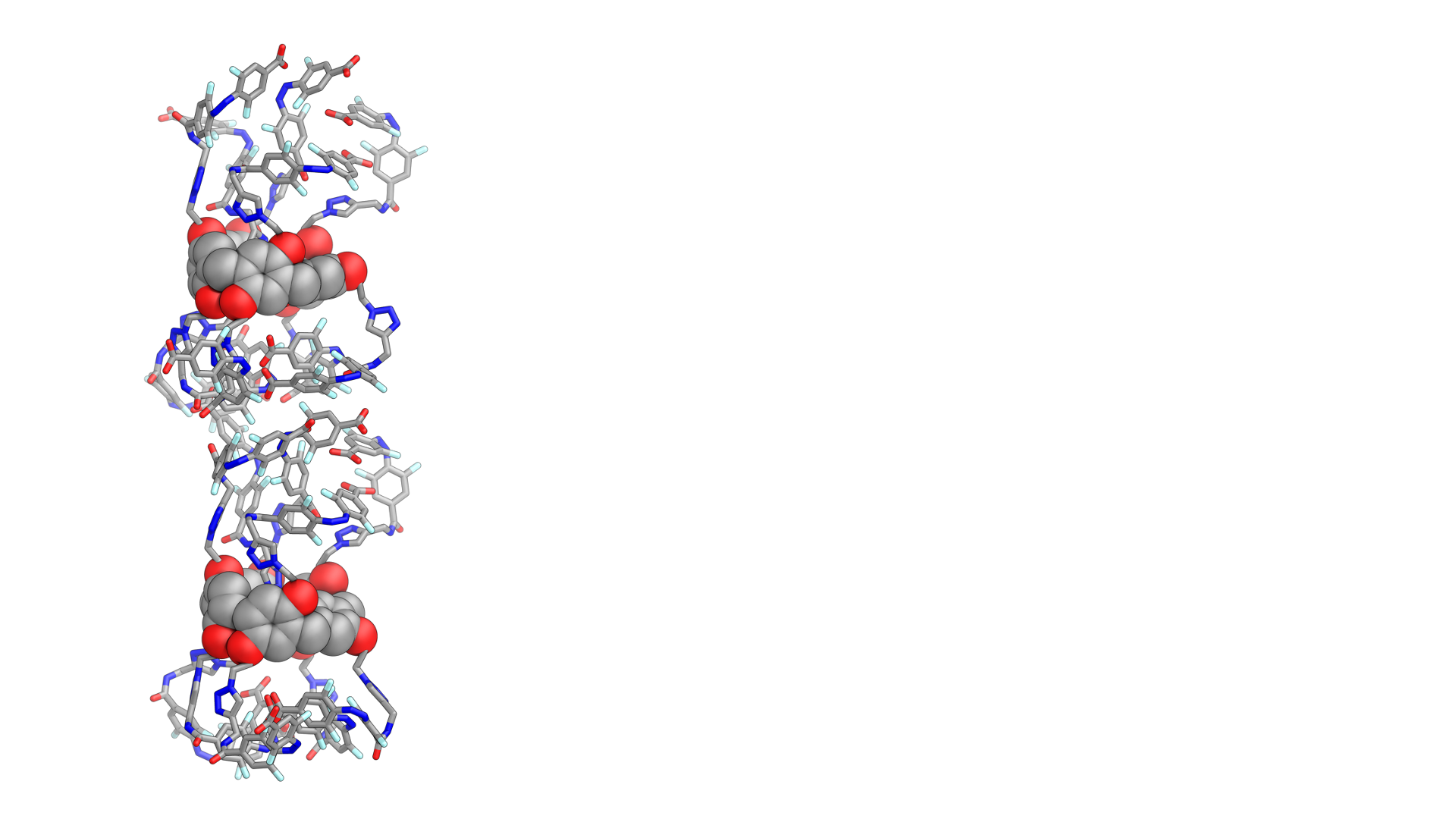
**Figure S71**. Side-view of GFN2-xTB optimised geometry of **3**-*Z* to normal energy convergence and octanol implicit solvation parameters at an electronic temperature of 300 K. Pillar[5]arene core is highlighted with space-filled spheres.

**A**

**D**

**C**

**B**

**Figure S72**. Facial-views of GFN2-xTB optimised geometries of (A) **3**-*E* isomer top face; (B) **3**-*E* isomer bottom face showing complete occlusion of the cavity; (C) **3**-*Z* isomer top face and (D) **3**-*Z* isomer bottom face (arbitrary assignment).

# References:

S1. A. Kerckhoffs, Z. Bo, S. Penty, F. Duarte, M. J. Langton. Red-Shifted Tetra-*ortho*-halo-azobenzenes for Photo-regulated Transmembrane Anion Transport. *Org. Biomol. Chem.* **2021***, 19*, 9058−9067. 10.1039/D1OB01457A

S2. I. Nierengarten, S. Guerra, M. Holler, J. Karmazin-Brelot, R. Deschenaux, RJ. F. Nierengarten. Macrocyclic Effects in the Mesomorphic Properties of Liquid-Crystalline Pillar[5]- and Pillar[6]arenes. *Eur. J. Org. Chem*. **2013**, *18*, 3675−3684. 10.1002/ejoc.201300356

S3. T. Saha, A. Gautam, A. Mukherjee, M. Lahiri, P. Talukdar. Chloride Transport through Supramolecular Barrel-Rosette Ion Channels: Lipophilic Control and Apoptosis-Inducing Activity*.* *J. Am. Chem. Soc*. **2016**, *138*, 16443−16451 10.1021/jacs.6b10379

# S4. E. R. Abdurakhmanova, D. Mondal, H. Jedrzejewska, P. Cmoch, O. Danylyuk, M. J. Chmielewski, A. Szumna. Supramolecular Umpolung: Converting Electron-rich Resorcin[4]arenes into Potent CH−bonding Anion Receptors and Transporters. *Chem*, 2024, *10*, 1910−1924. 10.1016/j.chempr.2024.03.003

S5. H. Li, J. A., Cooper, H. Valkenier, A. G. ThorneC. M. Dias; C. M.; M. Kieffer, P. A. Gale, N. Busschaert, D. A. Sheppard, A. P. Davis. Anion Carriers as Potential Treatments for Cystic Fibrosis: Transport in Cystic Fibrosis Cells, and Additivity to Channel-Targeting Drugs*.* *Chem. Sci.* **2019***, 10*, 9663−9672. 10.1039/c9sc04242c

S6. E. Grählert, M. J. Langton. Transmembrane Delivery of an Aryl Azopyrazole Photoswitchable Ion Transporter Relay*.* *Angew. Chem., Int. Ed.* **202**4, *64*, e202421580. 10.1002/anie.202421580

S7. X. Wu, XP. A. Gale. Measuring Anion Transport Selectivity: A Cautionary Tale. *Chem. Commun*. **2021**, *57*, 3979−3982. 10.1039/d1cc01038g

S8. T. Saha, S. Dasari, D. Tewari, A. Prathap, K.M. Sureshan, A. K. Bera, A. Mukherjee, P. Talukdar. Hopping-Mediated Anion Transport through a Mannitol-Based Rosette Ion Channel. *J. Am. Chem. Soc*. **2014**, *136*, 14128−14135. https://doi.org/10.1021/ja506278z

S10. J. A. Cooper, S. T. G. Street, A. P. Davis. A Flexible Solution to Anion Transport: Powerful Anionophores Based on a Cyclohexane Scaffold*.* *Angew. Chem., Int. Ed*. **2014**, *53*, 5609−5613. 10.1002/anie.201311071.

S11. X. Wu, P. A Gale. Small-Molecule Uncoupling Protein Mimics: Synthetic Anion Receptors as Fatty Acid-Activated Proton Transporters. *J. Am. Chem. Soc*. **2016**, *138*, 16508−16514. <https://doi.org/10.1021/jacs.6b10615>

S12. S. Litvinchuk, H. Tanaka, T. Miyatake, D. Pasini, T. Tanaka, G. Bollot, J. Mareda, S. Matile. Synthetic pores with reactive signal amplifiers as artificial tongues. *Nat. Mater*. **2007**, *6*, 576−580. https://doi.org/10.1038/nmat1933

S13. R. Paul, D. Dutta, M. Wallace, J. Dash. Ion Transport and Membrane Channel Formation using a Peptidomimetic in Droplet Interface Bilayers*.* *Chem. Commun*. **2025**, *61*, 3876−3879. 10.1039/d4cc05926c.

S14. A. G. Hawkes, A. Jalali, D. Colquhoun. The Distributions of the Apparent Open Times and Shut Times in a Single Channel Record when Brief Events Cannot be Detected. *Philosophical Transactions of the Royal Society of London. Series A: Physical and Engineering Sciences,* **1990**, *332*, 511–538. https://doi.org/10.1098/rsta.1990.0129

S15. F. Qin, A. Auerbach, F. Sachs. Maximum Likelihood Estimation of Aggregated Markov Processes. *Proceedings of the Royal Society of London. Series B: Biological Sciences* **1997**, *264*, 375–383, 1997. https://doi.org/10.1098/rspb.1997.0054

S16. S. Leptihn, O. K. Castell, B. Cronin, E. H. Lee, L. C. M. Gross, D. P. Marshall, J. R. Thompson, M. Holden, M. I. Wallace. Constructing Droplet Interface Bilayers from the Contact of Aqueous Droplets in Oil. *Nature Protocols* **2013**, *8*, 1048–1057. 10.1038/nprot.2013.061

S17. N. L. Allinger. Conformational Analysis. 130. MM2. A Hydrocarbon Force Field Utilizing V1 and V2 Torsional Terms. *J. Am. Chem. Soc.* **1977**, *99*, 8127−8134. 10.1021/ja00467a001

S18. N. M. O’Boyle, M. Banck, C. A. James, C. Morley, T Vandermeesch, G. Hutchinson. Open Babel: An Open Chemical Toolbox. *J. Cheminform.* **2011**, *3*, 33. 10.1186/1758-2946-3-33

S19. C. Bannwarth, S. Ehlert, S. Grimme. GFN2-xTB - An Accurate and Broadly Parametrized Self-Consistent Tight-Binding Quantum Chemical Method with Multipole Electrostatics and Density-Dependent Dispersion Contributions. *J. Chem. Theory Comput.* **2019**, *15*, 1652–1671. 10.1021/acs.jctc.8b01176

S20. S. Ehlert, M. Stahn, S. Spicher, S. Grimme. Robust and Efficient Implicit Solvation Model for Fast Semiempirical Methods. *J. Chem. Theory Comput.* **2021**, *17*, 4250–4261. 10.1021/acs.jctc.1c00471

S21. S. Grimme. Exploration of Chemical Compound, Conformer, and Reaction Space with Meta-Dynamics Simulations Based on Tight-Binding Quantum Chemical Calculations. J*. Chem. Theory Comput.* **2019**, *15*, 2847–2862. 10.1021/acs.jctc.9b00143
